# 6-Methoxyflavone targets SLC1A5 to induce ferroptosis in HeLa cells

**DOI:** 10.1371/journal.pone.0339578

**Published:** 2025-12-29

**Authors:** Chaihong Zhang, Lihong Chen

**Affiliations:** Department of Obstetrics and Gynecology, Shaanxi Provincial People’s Hospital, Xi’an, Shaanxi, China; UMass Chan Medical School Department of Medicine: University of Massachusetts Chan Medical School Department of Medicine, UNITED STATES OF AMERICA

## Abstract

**Objective:**

This study aimed to explore the role and molecular mechanism of 6-methoxyflavone in inducing ferroptosis in HeLa cells.

**Methods:**

Transmission electron microscopy (TEM), mitochondrial superoxide, and glutathione content assay were used to detect the effects of 6-methoxyflavone on ferroptosis. Tandem mass tag and parallel reaction monitoring proteomics, non-targeted and targeted metabolomics, polymerase chain reaction (qPCR), western blot, alternative splicing, new transcript, functional domain, molecular docking, non-covalent interaction, loss-of-function genetic manipulation, and mitochondrial superoxide analyses were performed to explore the molecular mechanism of 6-methoxyflavone-induced ferroptosis in HeLa cells.

**Results:**

6-Methoxyflavone induced ferroptosis in HeLa cells. Multi-omics, qPCR, western blot, alternative splicing, new transcript, functional domain, molecular docking, non-covalent interaction, loss-of-function genetic manipulation, and mitochondrial superoxide analyses indicated that 6-methoxyflavone induced ferroptosis in HeLa cells by upregulating the expression levels of SLC1A5 and mitochondrial superoxide. Molecular docking analyses showed that 6-methoxyflavone had the strongest affinity for SLC1A5. Non-covalent interaction analyses suggested that the interaction between 6-methoxyflavone and SLC1A5 was primarily driven by hydrophobic interactions. 6-Methoxyflavone targeted the peptide segment sequence LGPEGELLIR of SLC1A5.

**Conclusion:**

6-Methoxyflavone induced ferroptosis in HeLa cells by markedly altering ferroptosis-related genes, proteins, and metabolites expression, thereby exerting anti-cancer effects. The core gene responsible for the induction of ferroptosis and the upregulation of mitochondrial superoxide in HeLa cells by 6-methoxyflavone is SLC1A5.

## 1 Introduction

In 2022, the incidence and mortality rates of cervical cancer were relatively high worldwide [1]. Cervical cancer is a major health concern in women, and cervical cancer treatment is associated with many challenges. Medicinal plants are essential natural products, and drugs derived from natural products play a crucial role in cancer treatment. 6-Methoxyflavone is a small lipid-soluble molecule extracted from the Chinese herbal medicine *Imperata cylindrica* [2] and has inhibitory effects on various cancer cells, with the strongest effect on HeLa cells [3]. However, the role and mechanism of 6-methoxyflavone in HeLa cell ferroptosis remains unclear.

Ferroptosis is closely related to the growth, metastasis [4], immune cell [5], and treatment [6] of cervical cancer. Induction of cancer cell ferroptosis is a promising cancer treatment strategy [5]. Ferroptosis is a potential target for cancer treatment. Novel carbon quantum dots technology induces ferroptosis in cancer cells via antioxidant inhibition synergistic nanocatalytic activity [7]. Ropivacaine induces ferroptosis to upregulate the cisplatin-sensitivity of human colorectal cancer by the sirtuin 1 (SIRT1)/ nuclear factor erythroid 2-related factor 2 (Nrf2) pathway [8]. More and more small molecule compounds in traditional Chinese medicine have been found to exert anti-cancer effects through the ferroptosis pathway. Red ginseng polysaccharide induces ferroptosis in gastric cancer cells via the phosphatidylinositol 3-kinase (PI3K)/ protein kinase B (AKT) pathway [9]. Baicalein induces ferroptosis in colorectal cancer cells by the janus kinase 2 (JAK2)/ signal transducer and activator of transcription 3 (STAT3)/ glutathione peroxidase 4 (GPX4) pathway [10]. In our study, transmission electron microscopy imaging, mitochondrial superoxide assay, and reduced and oxidized glutathione content assay were used to detect the effects of 6-methoxyflavone on ferroptosis in HeLa cells.

An important characteristic of malignant tumors is that tumor cells undergo metabolic changes in materials and energy to adapt to various stresses [11]. Metabolic reprogramming is closely related to the malignant progression [12], immune microenvironment [13], diagnosis [14], and treatment [15] of tumors. Ferroptosis is closely associated with cancer metabolism [16] and affects cancer occurrence and progression. For example, icariin and curcumol synergistically induce autophagy and ferroptosis in prostate cancer cells, affecting lipid metabolism and exerting anticancer effects [17]. In our study, non-targeted metabolomics and targeted medical metabolomics were used to analyze differentially expressed target metabolites, and enriched pathways in HeLa cells after treatment with 6-methoxyflavone.

This study aimed to elucidate the potential role and molecular mechanism of 6-methoxyflavone in ferroptosis in HeLa cells. Tandem mass tag (TMT) proteomics, parallel reaction monitoring (PRM) proteomics, quantitative polymerase chain reaction (qPCR), western blot, alternative splicing, new transcript, functional domain, molecular docking, and non-covalent interaction analyses were performed to explore the molecular mechanism of 6-methoxyflavone-induced ferroptosis in HeLa cells. TMT and PRM proteomics analyses indicated that 6-methoxyflavone specifically interacted with the peptide sequence LGPEGELLIR of SLC1A5. Molecular docking analysis further confirmed a high binding affinity between 6-methoxyflavone and SLC1A5. Additionally, non-covalent interaction analysis suggested that the interaction between 6-methoxyflavone and SLC1A5 was primarily driven by hydrophobic interactions. Finally, TMT proteomics analysis showed that 6-methoxyflavone exerted effects on alternative splicing types, new transcript, and functional domain of SLC1A5.

## 2 Materials and methods

### 2.1 HeLa cells and 6-methoxyflavone

The human cervical adenocarcinoma HeLa cell line was obtained from Peking Union Medical College (Beijing, China). Fetal bovine serum (10%) was added to Dulbecco’s modified Eagle medium (HyClone, Logan, UT, USA) to culture HeLa cells. 6-Methoxyflavone extraction and purification were performed by Weikeqi Biotech (Chengdu, Sichuan, China). The 6-methoxyflavone powder was dissolved in dimethyl sulfoxide (DMSO) using ultrasound to prepare the stock solution.

### 2.2 Ethics statement

Our study did not involve research pertaining to human participants and did not include any human participants or human participants’ data. Our study did not encompass research related to animals and did not incorporate any data from animal participants.

### 2.3 Cell proliferation and toxicity assay

Single-cell suspensions were seeded in 96-well plates for 24 hours. Subsequently, the HeLa cells were treated with five concentrations of 6-methoxyflavone (0.16% DMSO, 20 μM, 40 μM, 80 μM, and 100 μM) for 24 and 48 hours. Following this, the cell counting kit 8 (CCK8) (MeilunBio, Dalian, Liaoning, China) was used to assess the toxicity of 6-methoxyflavone on HeLa cells. The optical density values were detected at 450 nm using a microplate reader MB580 (Huisong Heales, Shenzhen, Guangdong, China). The half-maximal inhibitory concentration (IC50) values were calculated using GraphPad Prism software (www.graphpad.com).

### 2.4 Mitochondrial superoxide assay

HeLa cells were cultured in six-well plates for 24 hours. Four concentrations (0.16% DMSO, 16.25 μM, 32.5 μM, and 65 μM) of 6-methoxyflavone were added to the complete medium and incubated for 48 hours. According to the manufacturer’s instructions, mSoxUp (Beyotime, Shanghai, China) was a positive control for inducing mitochondrial superoxide production in HeLa cells. 2 mL of mSoxUp working solution was added to each well and incubated for four hours at 37°C. MitoSOX Red (C43H43IN3P) fluorescence probe (Beyotime) was used to detect the percentage of HeLa cells in the different fluorescence intensity. According to the protocol, 500 μL of MitoSOX Red working solution was added to each sample tube and incubated for 30 minutes at 37°C. A flow cytometer Accuri™ C6 (BD Biosciences, San Jose, CA, USA) was used to detect the fluorescence intensity in the FL2 channel. FlowJo 10.8.1 (TreeStar, Ashland, CA, USA) and GraphPad Prism were used to analyze the fluorescence data.

### 2.5 Cell transfection and mitochondrial superoxide assay

HeLa cells were cultured in six-well plates for 24 hours. The culture medium was switched to a reduced serum medium (Gibco, Burlington, Canada). ASNS short hairpin RNA (shRNA) interference plasmid, SLC1A5 shRNA, and negative control shRNA (Genepharma, Shanghai, China) vectors were transfected into HeLa cells using Lipo8000 reagent (Beyotime) according to the instructions. All vectors contained the green fluorescent protein (GFP). After six hours, the medium was switched to a complete medium. After 24 hours, the HeLa cells was digested and collected for quantitative polymerase chain reaction (qPCR) analysis to detect transfection efficiency. The transfected cells were treated with 0.16% DMSO and 32.5 μM 6-methoxyflavone for 24 hours. The treated cells were collected for mitochondrial superoxide assay. The assay included two control groups: MitoSOX Red single fluorescence group of untreated HeLa cells, and GFP single fluorescence group of HeLa cells transfected with negative control shRNA. According to the protocol, 500 μL of MitoSOX Red working solution was added to each sample tube. A flow cytometer Accuri™ C6 was used to detect the fluorescence intensity in the FL1 and FL2 channels. FlowJo 10.8.1 and GraphPad Prism were used to analyze the fluorescence data.

### 2.6 TMT proteomic, non-targeted metabolomic, and targeted medical metabolomic analyses

The cells were collected to obtain samples after 48 hours of treatment with 0.16% DMSO or 65 μM 6-methoxyflavone. The treated cells were subjected to total protein or metabolite extraction, quality control, tandem mass tag (TMT; Thermo Fisher Scientific, Madison, WI, USA) proteomic, and non-targeted and targeted metabolomic analyses at Applied Protein Technology Biological (Shanghai, China). The TMT proteomic analysis was performed on a Q Exactive Orbitrap mass spectrometer (Thermo Fisher Scientific) that was coupled to Easy nLC chromatograph (Thermo Fisher Scientific) for 60 minutes. The raw data generated from mass spectrometry analyses are stored in.RAW files. The Mascot software (https://www.matrixscience.com/) and Proteome Discoverer software (Thermo Fisher Scientific) were employed for the identification of inventory and for conducting quantitative analyses. The TMT proteome detection includes three control group and three treatment group cell samples. A criterion with a fold change > 1.2 or < 0.84 and p < 0.05 was used to screen for significantly differentially expressed proteins. The proteins underwent Gene Ontology (GO) [18] analysis utilizing Blast2Go (https://www.blast2go.com/) software [19]. Differentially expressed GO biological processes were identified with a significance level of p < 0.05. The non-targeted metabolomic analysis was performed on a Q Exactive Orbitrap mass spectrometer (Thermo Fisher Scientific) that was coupled to Vanquish UHPLC chromatograph (Thermo Fisher Scientific) for 12 minutes. The raw data generated from mass spectrometry analyses are stored in.RAW files. The.RAW files were converted into.mzXML files by ProteoWizard software (https://proteowizard.sourceforge.io/). Peak alignment, retention time correction, and peak area extraction were conducted utilizing XCMS software (https://sciex.com/cl/products/software). Collection of Algorithms of MEtabolite pRofile Annotation was used for annotation of isotopes and adducts. The targeted metabolomic analysis was performed on a QTRAP 6500 + mass spectrometer (AB Sciex, Pte. Ltd., USA) that was coupled to 1290 Infinity chromatograph (Agilent Technologies, Inc. Winooski, VT, USA) for 15 or 22 minutes. MultiQuant or Analyst software (https://sciex.com/cl/products/software) was utilized to extract peaks from the raw data files, determine the peak areas of each metabolite, and calculate the metabolite concentrations based on the standard curve. Both non-targeted and targeted metabolomics detection include six control group and six treatment group cell samples. A criterion with variable importance for the projection (VIP) of orthogonal partial least squares discriminant analysis > 1 and p < 0.05 was used to screen for significantly differentially expressed non-targeted metabolites. A criterion with a fold change > 1.6 or < 0.63 and p < 0.05 was used to screen for significantly differentially expressed targeted metabolites. The metabolites were subjected to Kyoto Encyclopedia of Genes and Genomes (KEGG) analysis [20]. Differential KEGG pathways were identified at p < 0.05.

### 2.7 Observation of subcellular ultrastructure using transmission electron microscopy

HeLa cells were treated with 6-methoxyflavone similar to the cells used in the multi-omics analyses. These cells were produced as epoxy resin blocks and ultrathin sections. After staining with uranyl acetate and lead citrate, a transmission electron microscope HT7700 (Hitachi High-Tech, Tokyo, Japan) was used to observe the subcellular ultrastructure of the cells.

### 2.8 Differentially expressed metabolites related to ferroptosis

A list of metabolites related to ferroptosis was downloaded from the hsa04216 ferroptosis metabolic pathway in the KEGG official website (https://www.genome.jp/entry/pathway+hsa04216), and the expression data of ferroptosis-related metabolites was extracted from the non-targeted and targeted metabolomics data.

### 2.9 Reduced and oxidized glutathione content assay

HeLa cells were treated with 6-methoxyflavone similar to the cells used in the mitochondrial superoxide assay. The reduced and oxidized glutathione content assay kits were obtained from Boxbio Science ＆ Technology (Beijing, China). Reduced glutathione (GSH, 10 mg/mL) and oxidized glutathione (GSSG, 10 mg/mL) were utilized as standard substances. GSH: The treated cells (20 μl)were added in a 180 μl mixture containing 140 μl reagent 2 and 40 μl reagent 3. After two minutes of incubation, the optical density values were detected at 405 nm using a microplate reader MB580. GSSG: The treated cells (20 μl)were added in a 182 μl mixture containing 140 μl reagent 3, 20μl reagent 4, 20μl reagent 5, and 2 μl reagent 6. After 150 seconds of incubation, the optical density values were detected at 405 nm using a microplate reader MB580.

### 2.10 Cell apoptosis assay

The HeLa cells were treated with four concentrations of 6-methoxyflavone (0.16% DMSO, 16.25 μM, 32.5 μM, and 65 μM) for 48 hours. An annexin V-allophycocyanin (APC)/ propidium iodide (PI) apoptosis detection kit (BioLegend, San Diego, CA, USA) was used to detect and count the percentage of the apoptotic HeLa cells. The collected cells were suspended in a 315μl buffer containing 5μl of annexin V-APC and 10μl of PI. After 15 minutes of incubation, the cells were detected by a flow cytometer Accuri™ C6 (BD Biosciences, San Jose, CA, USA). FlowJo 10.8.1 (TreeStar, Ashland, CA, USA) (https://www.flowjo.com/) was used to calculate the percentage of the apoptotic HeLa cells.

### 2.11 Quantitative polymerase chain reaction

A list of ferroptosis-related genes was downloaded from the FerrDb database [21]. The expression data of ferroptosis-related proteins was obtained from the TMT proteome, and a list of differentially expressed ferroptosis-related proteins was acquired. Another list of ferroptosis-related genes was downloaded from the KEGG official website (https://www.genome.jp/entry/pathway+hsa04216). The coding genes of differentially expressed proteins in the TMT proteome should be intersected with the two specified gene lists individually. These ferroptosis-related intersection genes were analyzed for their expression levels by quantitative polymerase chain reaction (qPCR). HeLa cells were treated with 0.16% DMSO or 32.5 μM 6-methoxyflavone for 48 hours. Total RNA was extracted from treated cells using centrifugal column RNA extraction kit (Beyotime). The complementary DNA was obtained by Evo M-MLV reverse transcription premixed kit (Agbio, Changsha, Hunan, China). Real-time qPCR was performed using the SYBR Green Pro Taq HS premixed qPCR kit (Agbio) and the real-time qPCR system TL988 (Tianlong, Xi’an, Shaanxi, China). Glyceraldehyde 3 phosphate dehydrogenase (GAPDH) (Sangon Biotech, Shanghai, China) was used as housekeeping gene. The Delta-Delta Ct method was used to analyze the qPCR data. The qPCR primer sequences are listed in **Table 1**.

**Table 1:** The forward and reverse primer sequences for PCR.

Primers (human)	forward	reverse
Acyl-CoA synthetase long chain family member 4 (ACSL4)	CATCCCTGGAGCAGATACTCT	TCACTTAGGATTTCCCTGGTCC
Albumin (ALB)	TGCAACTCTTCGTGAAACCTATG	ACATCAACCTCTGGTCTCACC
Arachidonate 15-lipoxygenase (ALOX15)	GGGCAAGGAGACAGAACTCAA	CAGCGGTAACAAGGGAACCT
Asparagine synthetase (glutamine-hydrolyzing) (ASNS)	GGAAGACAGCCCCGATTTACT	AGCACGAACTGTTGTAATGTCA
Aurora kinase A (AURKA)	GAGGTCCAAAACGTGTTCTCG	ACAGGATGAGGTACACTGGTTG
Capping actin protein, gelsolin like (CAPG)	AGTCAGCATTTCACAAGACCTC	CACCACACCAGGCGAAGAT
Caveolin 1 (CAV1)	GCGACCCTAAACACCTCAAC	ATGCCGTCAAAACTGTGTGTC
Cold inducible RNA binding protein (CIRBP)	AGGGCTGAGTTTTGACACCAA	ACAAACCCAAATCCCCGAGAT
Ceruloplasmin (CP)	GGGCCATCTACCCTGATAACA	TTAAAGGTCCGATGAGTCCTGA
Epidermal growth factor receptor (EGFR)	AGGCACGAGTAACAAGCTCAC	ATGAGGACATAACCAGCCACC
Fatty acid desaturase 1 (FADS1)	CTACCCCGCGCTACTTCAC	CGGTCGATCACTAGCCACC
Ferritin mitochondrial (FTMT)	TGGAGTGTGCTCTACTCTTGG	ACGTGGTCACCTAGTTCTTTGA
GA binding protein transcription factor subunit beta 1 (GABPB1)	TCCACTTCATCTAGCAGCACA	GTAATGGTGTTCGGTCCACTT
Glutamate-cysteine ligase catalytic subunit (GCLC)	GGAGGAAACCAAGCGCCAT	CTTGACGGCGTGGTAGATGT
Glutamate-cysteine ligase modifier subunit (GCLM)	TGTCTTGGAATGCACTGTATCTC	CCCAGTAAGGCTGTAAATGCTC
Growth differentiation factor 15 (GDF15)	GACCCTCAGAGTTGCACTCC	GCCTGGTTAGCAGGTCCTC
Glutamic-oxaloacetic transaminase 1 (GOT1)	ATGGCACCTCCGTCAGTCT	AGTCATCCGTGCGATATGCTC
Glutamic--pyruvic transaminase 2 (GPT2)	GTGATGGCACTATGCACCTAC	TTCACGGATGCAGTTGACACC
Glutathione peroxidase 4 (GPX4)	GAGGCAAGACCGAAGTAAACTAC	CCGAACTGGTTACACGGGAA
Glutathione synthetase (GSS)	GGGAGCCTCTTGCAGGATAAA	GAATGGGGCATAGCTCACCAC
High mobility group box 1 (HMGB1)	GCCTTCTTCCTCTTCTGCTCTGAG	CTTCGCAACATCACCAATGGACAG
Isocitrate dehydrogenase (NADP(+)) 1 (IDH1)	CACCAAATGGCACCATACGAA	CCCCATAAGCATGACGACCTAT
Lysophosphatidylcholine acyltransferase 3 (LPCAT3)	GGAGCTGAGCCTTAACAAGTT	CAAAGCAAAGGGGTAACCCAG
Matrix metallopeptidase 13 (MMP13)	ACTGAGAGGCTCCGAGAAATG	GAACCCCGCATCTTGGCTT
N-myc downstream regulated 1 (NDRG1)	CTCCTGCAAGAGTTTGATGTCC	TCATGCCGATGTCATGGTAGG
Parkinsonism associated deglycase (PARK7)	GTAGCCGTGATGTGGTCATTT	CTGTGCGCCCAGATTACCT
Poly(ADP-ribose) polymerase 1 (PARP1)	TCTGAGCTTCGGTGGGATGA	TTGGCATACTCTGCTGCAAAG
Peroxisomal biogenesis factor 6 (PEX6)	CCCTTTCCGACCGAGACAC	CGCGGCTAACCAGTAGCTG
Pirin (PIR)	GAGCAGTCGGAAGGGGTTG	TTAACTCGGGTCTGCCAATGC
Perilipin 2 (PLIN2)	ATGGCATCCGTTGCAGTTGAT	GGACATGAGGTCATACGTGGAG
Ribonucleotide reductase regulatory subunit M2 (RRM2)	CACGGAGCCGAAAACTAAAGC	TCTGCCTTCTTATACATCTGCCA
Stearoyl-CoA desaturase (SCD)	TCTAGCTCCTATACCACCACCA	TCGTCTCCAACTTATCTCCTCC
Solute carrier family 1 member 5 (SLC1A5)	GAGCTGCTTATCCGCTTCTTC	GGGGCGTACCACATGATCC
Synuclein alpha (SNCA)	AAGAGGGTGTTCTCTATGTAGGC	GCTCCTCCAACATTTGTCACTT
Stathmin 1 (STMN1)	TCAGCCCTCGGTCAAAAGAAT	TTCTCGTGCTCTCGTTTCTCA
Transferrin (TF)	GTGTGCAGTGTCGGAGCAT	CATCGGATGGAATGACGCTTT
Transferrin receptor (TFRC)	ACCATTGTCATATACCCGGTTCA	CAATAGCCCAAGTAGCCAATCAT
Thymosin beta 4 X-linked (TMSB4X)	CTGACAAACCCGATATGGCTGAG	GATTCGCCTGCTTGCTTCTCC
Valosin containing protein (VCP)	CAAACAGAAGAACCGTCCCAA	TCACCTCGGAACAACTGCAAT
Voltage dependent anion channel 2 (VDAC2)	GGCGTGGAATTTTCAACGTCC	AGACCATACTCACACCACTTGTA
Voltage dependent anion channel 3 (VDAC3)	TTGTACCGAACACAGGAAAGAAG	CCCAGCCATAGATGGTTGGTC
Yes1 associated transcriptional regulator (YAP1)	TAGCCCTGCGTAGCCAGTTA	TCATGCTTAGTCCACTGTCTGT
Tyrosine 3-monooxygenase/tryptophan 5-monooxygenase activation protein epsilon (YWHAE)	TGCGGAGAACAGCCTAGTG	CCTAAGCGAATAGGATGCGTT

### 2.12 Western blot analysis

The HeLa cells were treated with three concentrations of 6-methoxyflavone (0.16% DMSO, 32.5 μM, and 65 μM) for 48 hours. The treated cells were lysed for 30 minutes in lysis buffer (Beyotime). A bicinchoninic acid protein assay kit (Beyotime) was used to measure protein concentrations. Protein lysates were separated by 12 wells polyacryl amide gel electrophoresis (GenScript, Nanjing, Jiangsu, China) and transferred onto 0.22 μm polyvinylidene fluoride membranes (PALL, New York, NY, USA).

After blocking with 5% blotting grade non-fat powdered milk solutions for 1 hour at room temperature, the membranes were soaked in the primary antibodies overnight at 4℃. The primary antibodies are listed in **Table 2**. The membranes were soaked in rabbit anti-goat IgG (Sangon Biotech) for 1 hour at room temperature. The prestained marker of western blot was purchased from HaiGene (Harbin, Heilongjiang, China). Finally, the protein bands were visually detected with a NcmECL ultra efficient chemiluminescence kit (New Cell & Molecular Biotech, Suzhou, Jiangsu, China) using the Alpha Innotech FluorChem FC2 imaging system (Bio-Techne, Minneapolis, MN, USA).

**Table 2:** Antibody and dilution for western blot.

Primary antibody	Primary antibody source	Primary antibody dilution	Secondary antibody	Secondary antibody dilution	Company
ASNS	Rabbit	1/1000	HRP-conjugated Goat anti rabbit IgG	1/5000	Sangon Biotech, Shanghai, China
GAPDH	Rabbit	1/5000	HRP-conjugated Goat anti rabbit IgG	1/5000	Sangon Biotech, Shanghai, China
GPT2	Rabbit	1/400	HRP-conjugated Goat anti rabbit IgG	1/5000	Sangon Biotech, Shanghai, China
PEX6	Rabbit	1/1000	HRP-conjugated Goat anti rabbit IgG	1/5000	Immunoway Biotech, SuZhou, China
					
RRM2	Rabbit	1/1000	HRP-conjugated Goat anti rabbit IgG	1/5000	Immunoway Biotech, SuZhou, China
SCD	Rabbit	1/600	HRP-conjugated Goat anti rabbit IgG	1/5000	Sangon Biotech, Shanghai, China
SLC1A5	Rabbit	1/2000	HRP-conjugated Goat anti rabbit IgG	1/5000	Proteintech Biotech, Shanghai, China
TMSB4X	Rabbit	1/1000	HRP-conjugated Goat anti rabbit IgG	1/5000	Immunoway Biotech, SuZhou, China
VCP	Rabbit	1/300	HRP-conjugated Goat anti rabbit IgG	1/5000	Sangon Biotech, Shanghai, China
VDAC3	Rabbit	1/800	HRP-conjugated Goat anti rabbit IgG	1/5000	Sangon Biotech, Shanghai, China

### 2.13 PRM proteomic quantitative analysis of target proteins

The HeLa cells were treated with two concentrations of 6-methoxyflavone (0.16% DMSO and 65 μM) for 48 hours. Parallel reaction monitoring (PRM) protein quantitative detection include three control group and three treatment group cell samples. PRM was used to quantitatively detect the expression levels of differentially expressed ferroptosis-related proteins. Mass spectrometry (MS) was performed at Applied Protein Technology Biotech (Shanghai, China). The PRM proteomic analysis was performed on a Q Exactive Orbitrap mass spectrometer (Thermo Fisher Scientific) that was coupled to Easy nLC system (Thermo Fisher Scientific) for 60 minutes. Skyline 3.5.0 software [22] was used to quantify peptide segment expression levels. Finally, the expression data for these ferroptosis-related proteins was extracted from TMT and PRM proteomics.

### 2.14 Functional domain analyses of ferroptosis-related proteins

After treatment with 65 μM 6-methoxyflavone for 48 hours, InterProScan software [23] was used to perform functional characterization of the sequences detected by MS to obtain domain annotation information of the target sequences in the Pfam database [24].

### 2.15 Structural changes in ferroptosis-related genes

After treatment with 65 μM 6-methoxyflavone for 48 hours, the HeLa cells were subjected to total RNA extraction, quality control, RNA library construction, alternative splicing, and new transcript analyses at Sangon Biological (Shanghai, China). The Illumina HiSeq platform (Illumina, San Diego, California, USA), ASprofier [25], StringTie [26], and GffCompare [27] softwares were used for these analyses.

### 2.16 Molecular docking and interaction analyses

The 6-methoxyflavone structure was downloaded from PubChem [28]. ASNS and SLC1A5 structures were downloaded from AlphaFold [29,30]. AutoDock Vina [31] was used to perform molecular docking of 6-methoxyflavone with the two target proteins. Protein–Ligand Interaction Profiler [32] was used to identify non-covalent interactions between 6-methoxyflavone and the two target proteins. The inhibition constant and dissociation constant were calculated based on the free energy required for molecular docking.

### 2.17 Statistical analysis

All experiments were conducted with three biological replicates. Six replicates were set for each biological repetition in the CCK8 experiment. Three replicates were set for each biological repetition in the qPCR experiments. GraphPad Prism (v.8.4.3) was used for statistical analyses. The testing methods included unpaired t-tests, paired t-tests, and one-way analysis of variance (ANOVA). The GO function and KEGG pathway enrichment analyses were conducted utilizing Fisher’s Exact Test. Statistical significance was set at p < 0.05. What’s more, in this study, the negative control treatment selected was 0.16% DMSO [33].

## 3 Results

### 3.1 6-Methoxyflavone inhibits the proliferation of HeLa cells

**Fig 1:**
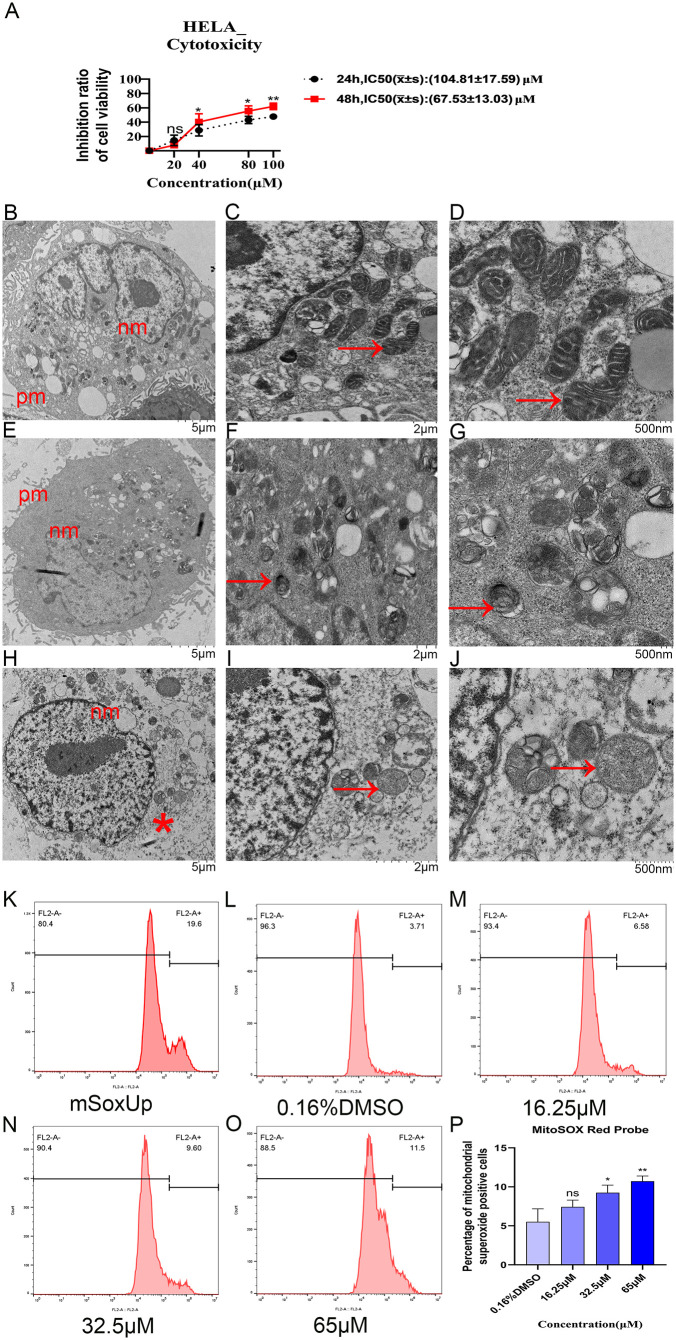
6Methoxyflavone inhibits cell proliferation, induces ferroptosis, and upregulates the expression level of mitochondrial superoxide in HeLa cells. A. 6-Methoxyflavone inhibits cell viability and proliferation in HeLa cells. The cells were treated with five concentrations of 6-methoxyflavone (0.16% DMSO, 20 μM, 40 μM, 80 μM, and 100 μM) for 24 and 48 hours. The cell counting kit 8 (CCK8) was used to assess the cell viability and proliferation of 6-methoxyflavone on HeLa cells. The half-maximal inhibitory concentration (IC50) values were determined utilizing GraphPad Prism. All experiments were carried out with three biological replicates, and each biological replicate was accompanied by six technical replicates. *p < 0.05. **p < 0.01. ns: not significant. DMSO: dimethyl sulfoxide. B-J. 6-Methoxyflavone induces ferroptosis in HeLa cells. Transmission electron microscopy images of HeLa cells after treatment with 6-methoxyflavone. All assessments were repeated at least thrice. B-D: Normal HeLa cell control group treated with 0.16% DMSO. E–J: HeLa cells treated with 65 μM 6-methoxyflavone for 48 hours. E-G: Early stage of ferroptosis: Intact cytoplasmic and nuclear membrane, increased mitochondrial membrane density, reduced mitochondrial volume, and decreased or disappeared mitochondrial cristae. H-J: Late stage of ferroptosis: Cytoplasmic membrane rupture, intact nuclear membrane, mitochondrial wrinkling, increased mitochondrial membrane density, decreased or disappeared mitochondrial cristae. pm: cytoplasmic membrane; nm: nuclear membrane; *: cytoplasmic membrane rupture. Arrows indicate mitochondria. K-P: 6-Methoxyflavone upregulates the expression level of mitochondrial superoxide in HeLa cells. The cells were treated with four concentrations of 6-methoxyflavone (0.16% DMSO, 16.25 μM, 32.5 μM, and 65 μM) for 48 hours. All experiments were performed using three biological replicates. Statistical analysis was performed using a one-way analysis of variance (ANOVA). K-O. 6-Methoxyflavone upregulates the fluorescence intensity of mitochondrial superoxide in HeLa cells. The mSoxUp was a positive control included with the kit. The flow cytometer and mitochondrial superoxide assay kit were used to detect the fluorescence intensity of MitoSOX Red probe. **P.** The histogram shows the percentage of mitochondrial superoxide positive cells in Fig 1K-1O. *p < 0.05. **p < 0.01. ns: not significant. x̄ ± s: mean ± standard deviation.

Following the treatment of HeLa cells with five concentrations of 6-methoxyflavone (0.16% DMSO, 20 μM, 40 μM, 80 μM, and 100 μM) for durations of 24 and 48 hours, we evaluated cell viability using the CCK8 assay. The findings indicated that 6-methoxyflavone inhibited the viability and proliferation of HeLa cells. **Fig 1A** demonstrates the inhibitory effect of 6-methoxyflavone on HeLa cells after 24 and 48 hours of treatment. The IC50 values were 104.81 μM and 67.53 μM, respectively. Consequently, we selected a duration of 48 hours for the subsequent assays and determined an approximate concentration of 67.53 μM, which is roughly equivalent to 65 μM, for the following experiments.

### 3.2 6-Methoxyflavone induces ferroptosis in HeLa cells

After treatment with 65 μM of 6-methoxyflavone, differential expression profiles of 260 metabolites were obtained by non-targeted metabolomic analysis. KEGG pathway enrichment analysis of the 260 metabolites indicated that 6-methoxyflavone was significantly correlated with ferroptosis, glutathione metabolism, and alanine, aspartate, and glutamate metabolic pathways. Differential expression profiles of 82 metabolites were obtained by targeted metabolomic analysis. KEGG analysis of the 82 metabolites indicated that 6-methoxyflavone was significantly correlated with ferroptosis, unsaturated fatty acid biosynthesis, D-glutamine and D-glutamate metabolism, and alanine, aspartate, and glutamate metabolic pathways. Fatty acid, alanine, aspartate, glutamate, glutathione, D-Glutamine, and D-glutamate metabolism are closely related to ferroptosis, and are all ferroptosis-related metabolites. 6-Methoxyflavone was markedly correlated with the ferroptosis pathway (**Table 3**).

**Table 3:** Functional or pathway enrichment analyses of multi-omics.

Analysis	Term ID	Term Name	Test/Ref	p
TMT proteomics Functional enrichment	GO:0006801	Superoxide metabolic process	5/22	0.03
	GO:0090322	Regulation of superoxide metabolic process	4/14	0.02
	GO:0000303	Response to superoxide	4/14	0.02
	GO:0071451	Cellular response to superoxide	4/13	0.02
	GO:0019430	Removal of superoxide radicals	4/11	0.009
	GO:2000121	Regulation of removal of superoxide radicals	2/4	0.03
Non-targeted metabolomics Pathway enrichment	hsa04216	**Ferroptosis**	5/29	0.004
	hsa00250	Alanine, aspartate and glutamate metabolism	6/28	0.0005
	hsa00480	Glutathione metabolism	5/38	0.01
Targeted metabolomics Pathway enrichment	hsa04216	**Ferroptosis**	5/29	0.0001
	hsa01040	Biosynthesis of unsaturated fatty acids	9/74	3.7E-06
	hsa00471	D-Glutamine and D-glutamate metabolism	2/13	0.02
	hsa00250	Alanine, aspartate and glutamate metabolism	4/28	0.001

Abbreviations: Test/Ref, Number of differentially expressed proteins or metabolites/total number of proteins or metabolites in the term.

Transmission electron microscopy (TEM) is a crucial tool for detecting ferroptosis. TEM can directly observe the characteristic morphological features of ferroptosis and the differences in subcellular ultrastructure, making it an important criterion for identifying ferroptosis. TEM can be used to distinguish between apoptosis, necrosis, and ferroptosis [34]. The typical TEM manifestations of apoptosis include the formation of chromatin margination and apoptotic bodies, while mitochondria appear normal or swollen [34,35]. In contrast, cell necrosis is characterized by rupture of the cytoplasmic and nuclear membranes, accompanied by swollen mitochondria [34,36]. Typical TEM features of ferroptosis include mitochondrial shrinkage, reduced mitochondrial volume, and decreased or absent mitochondrial cristae [34,37]. HeLa cells treated with 0.16% DMSO had normal cytoplasmic and nuclear membranes, mitochondria, and mitochondrial cristae (Fig 1B–1D). However, after treatment with 6-methoxyflavone, ferroptosis morphological changes at various stages were observed in HeLa cells, such as cytoplasmic membrane rupture, mitochondrial wrinkling, increased mitochondrial membrane density, reduced mitochondrial volume, and decreased or disappeared mitochondrial cristae (Fig 1E–1J). 6-Methoxyflavone primarily exerts its effects on the mitochondria of HeLa cells. These TEM images indicated that 6-methoxyflavone markedly induced ferroptosis in HeLa cells (Fig 1B-1J).

After treatment with 65 μM of 6-methoxyflavone, differential expression profiles of 526 proteins were obtained by TMT proteomic analysis. GO analysis of the 526 proteins indicated that 6-methoxyflavone was significantly correlated with superoxide (**Table 3**).

Mitochondrion superoxide is important detection indicators for ferroptosis. The results of the mitochondrion superoxide assay showed that both 32.5 μM and 65 μM of 6-methoxyflavone significantly upregulated the expression level of superoxide in the mitochondria of HeLa cells (Fig 1K–1O). 6-Methoxyflavone exhibited a specific impact on mitochondria (Fig 1K–1P).

**Fig 2:**
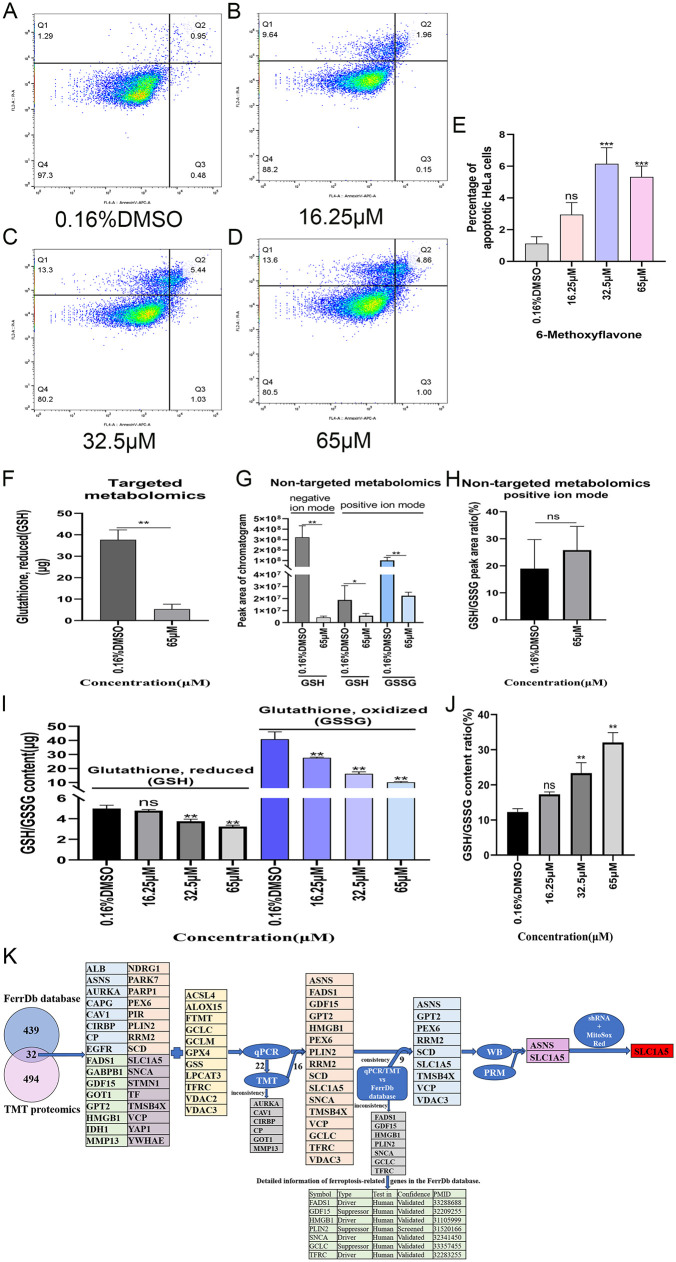
The cell apoptosis assay, the expression level of glutathione, and the flowchart for exploring the molecular mechanism. A-E. The cell apoptosis assay. A-D. After annexin V-allophycocyanin/ propidium iodide staining, the percentage of HeLa cells in different stages was measured by flow cytometer. **E.** The histogram shows the percentage of the apoptotic HeLa cells in A-D. Statistical analysis was performed using a one-way analysis of variance (ANOVA). ns: no significance; ^*^: p < 0.05; ^**^: p < 0.01; ^***^: p < 0.001. Three biological replicates were performed for all experiments. F-J. The expression levels of reduced glutathione (GSH) and oxidized glutathione (GSSG). All assays were repeated at least thrice. *p < 0.05. **p < 0.01. ns: not significant. F-H. The HeLa cells were treated with two concentrations of 6-methoxyflavone (0.16% DMSO and 65 μM) for 48 hours. Statistical analysis was performed using unpaired t-tests. **F.** The expression levels of GSH in targeted metabolomics. **G.** The expression levels of GSH and GSSG in non-targeted metabolomics. **H.** The ratio of GSH/GSSG in positive ion mode of non-targeted metabolomics analysis. I-J. The HeLa cells were treated with four concentrations of 6-methoxyflavone (0.16% DMSO, 16.25 μM, 32.5 μM, and 65 μM) for 48 hours. The GSH and GSSG content assays and a microplate reader were used to detect the content of GSH and GSSG. Statistical analysis was performed using a one-way analysis of variance (ANOVA). **J.** The ratios of GSH/GSSG in the GSH and GSSG content assays. **K.** The flowchart for exploring the molecular mechanism of 6-methoxyflavone induced ferroptosis in HeLa cells.

We measured the percentage of the apoptotic HeLa cells using the Annexin V-APC/PI apoptosis detection kit. The findings from the flow cytometry analysis indicated significant increases in the apoptosis rate of HeLa cells following treatment with 6-methoxyflavone at concentrations of 32.5 μM and 65 μM, in comparison to the 0.16%DMSO group. However, no significant alterations were observed with the 6-methoxyflavone at 16.25 μM (Fig 2A-2E).GSH and GSSG are important detection indicators for ferroptosis. Glutathione consumption is the core indicator of ferroptosis. Glutathione depletion directly leads to the accumulation of peroxides, driving ferroptosis [38]. The targeted metabolomics analysis indicated a significant downregulation in the expression level of GSH after treatment with 65 μM of 6-methoxyflavone (**Fig 2F**). Additionally, the non-targeted metabolomics analysis revealed that the expression levels of GSH and GSSG were significantly downregulated after treatment with 65 μM of 6-methoxyflavone (**Fig 2G**). Furthermore, the non-targeted metabolomics analysis in positive ion mode showed that the GSH/GSSG ratio did not significantly change after treatment with 6-methoxyflavone (**Fig 2H**). The assay for measuring the content of reduced and oxidized glutathione indicated that the levels of GSH were significantly downregulated by 6-methoxyflavone at concentrations of 32.5 μM and 65 μM. Additionally, the levels of GSSG were significantly downregulated by 6-methoxyflavone at concentrations of 16.25 μM, 32.5 μM, and 65 μΜ (**Fig 2I**). Furthermore, the GSH/GSSG ratio was significantly upregulated by 6-methoxyflavone at concentrations of 32.5 μM and 65 μM (**Fig 2J**).

### 3.3 6-Methoxyflavone alters ferroptosis-related metabolites expression

Arachidonic acid, adrenic acid, glutathione (reduced), glutathione (oxidized), glutamate, cysteine, and glutamine are closely related to ferroptosis, and are all ferroptosis-related metabolites. 6-Methoxyflavone significantly upregulated arachidonic acid and adrenic acid expression, whereas it significantly downregulated glutathione (reduced), glutathione (oxidized), glutamate, cysteine, and glutamine expression (**Table 4,** S1–S4 Files). 6-Methoxyflavone markedly altered ferroptosis-related metabolites expression.

**Table 4:** Differential expression metabolites related to ferroptosis.

Mode	KEGG_ID	Name	Fold change	p	VIP
Targeted metabolomics	C00051	Glutathione, reduced	0.14	2.66E-08	—
Non-targeted metabolomics (negative ion mode)	C00051	Glutathione, reduced	0.01	3.31E-05	4.64
Non-targeted metabolomics (positive ion mode)	C00051	Glutathione, reduced	0.30	0.02	0.54
Non-targeted metabolomics (positive ion mode)	C00127	Glutathione, oxidized	0.22	4.27E-05	1.54
Non-targeted metabolomics (positive ion mode)	C00025	Glutamate	0.68	3.34E-06	4.02
Non-targeted metabolomics (negative ion mode)	C00025	Glutamate	0.74	2.42E-05	4.43
Targeted metabolomics	C00025	Glutamate	0.33	1.63E-09	—
Non-targeted metabolomics (negative ion mode)	C00219	Arachidonic acid	2.98	0.0006	4.52
Targeted metabolomics	C00219	Arachidonic acid	2.27	0.001	—
Targeted metabolomics	C16527	Adrenic acid	2.71	0.0002	—
Targeted metabolomics	C00491	Cystine	0.54	0.004	—
Non-targeted metabolomics (positive ion mode)	C00064	D-glutamine	0.12	2.53E-09	6.63
Non-targeted metabolomics (negative ion mode)	C00303	DL-glutamine	0.08	1.34E-08	6.97
Targeted metabolomics	C00064	Glutamine	0.08	8.16E-09	—

### 3.4 6-Methoxyflavone alters expression of ferroptosis-related genes and proteins

Mitochondrion superoxide assay, transmission electron microscopy imaging, GSH and GSSG content assay, non-targeted and targeted metabolomic analyses all indicated 6-methoxyflavone induced ferroptosis in HeLa cells. Therefore, we conducted TMT proteomics to explore the molecular mechanism of 6-methoxyflavone induced ferroptosis in HeLa cells. After treatment with 65 μM of 6-methoxyflavone, differential expression profiles of 526 proteins were obtained by TMT proteomic analysis.

**Fig 3:**
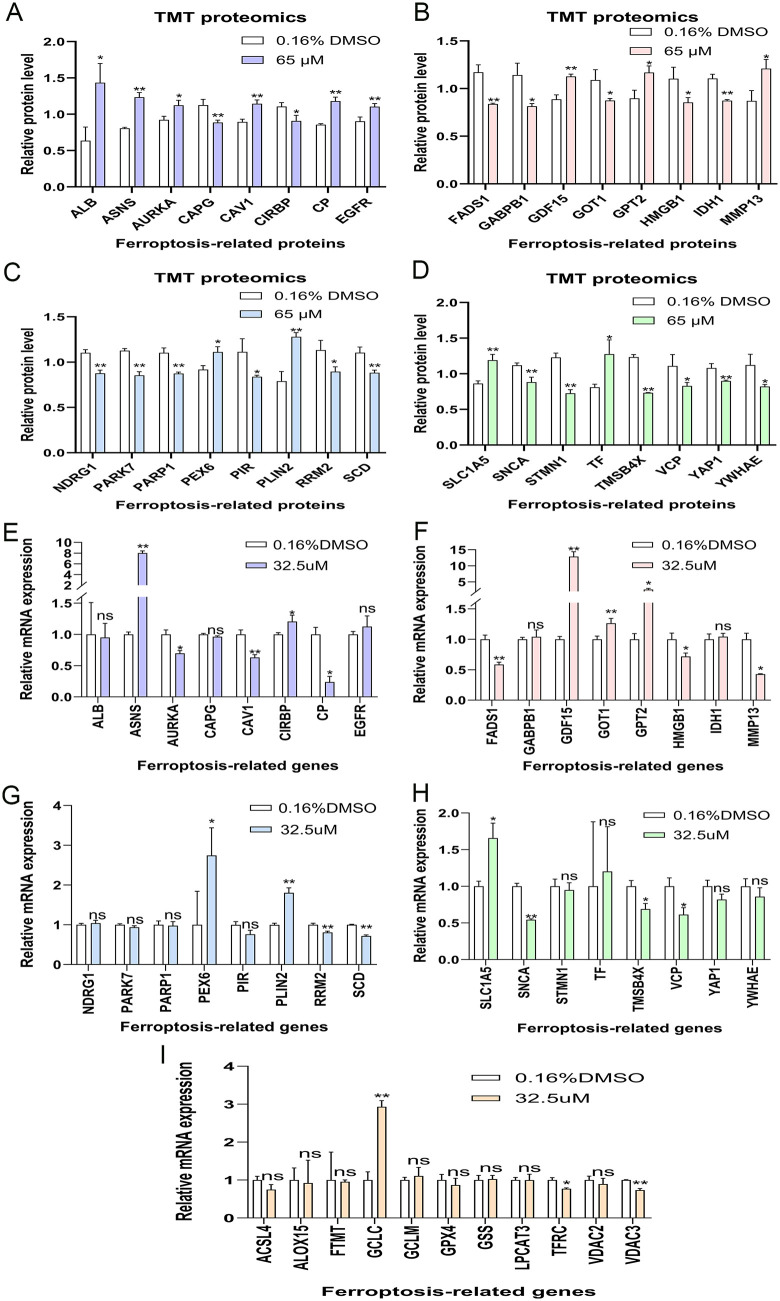
The expression levels of human ferroptosis-related proteins and genes. A-D. The expression levels of 32 significantly differentially expressed human ferroptosis-related proteins in the tandem mass tag (TMT) proteome. The HeLa cells were treated with two concentrations of 6-methoxyflavone (0.16% DMSO and 65 μM) for 48 hours. Statistical analysis was performed using t-tests. E-I. The expression levels of 43 human ferroptosis-related genes in the quantitative polymerase chain reaction (qPCR) assays. The HeLa cells were treated with two concentrations of 6-methoxyflavone (0.16% DMSO and 32.5 μM) for 48 hours. Statistical analysis was performed using paired t-tests. All assays were repeated at least thrice. *p < 0.05. **p < 0.01. ns: not significant.

**Fig 4:**
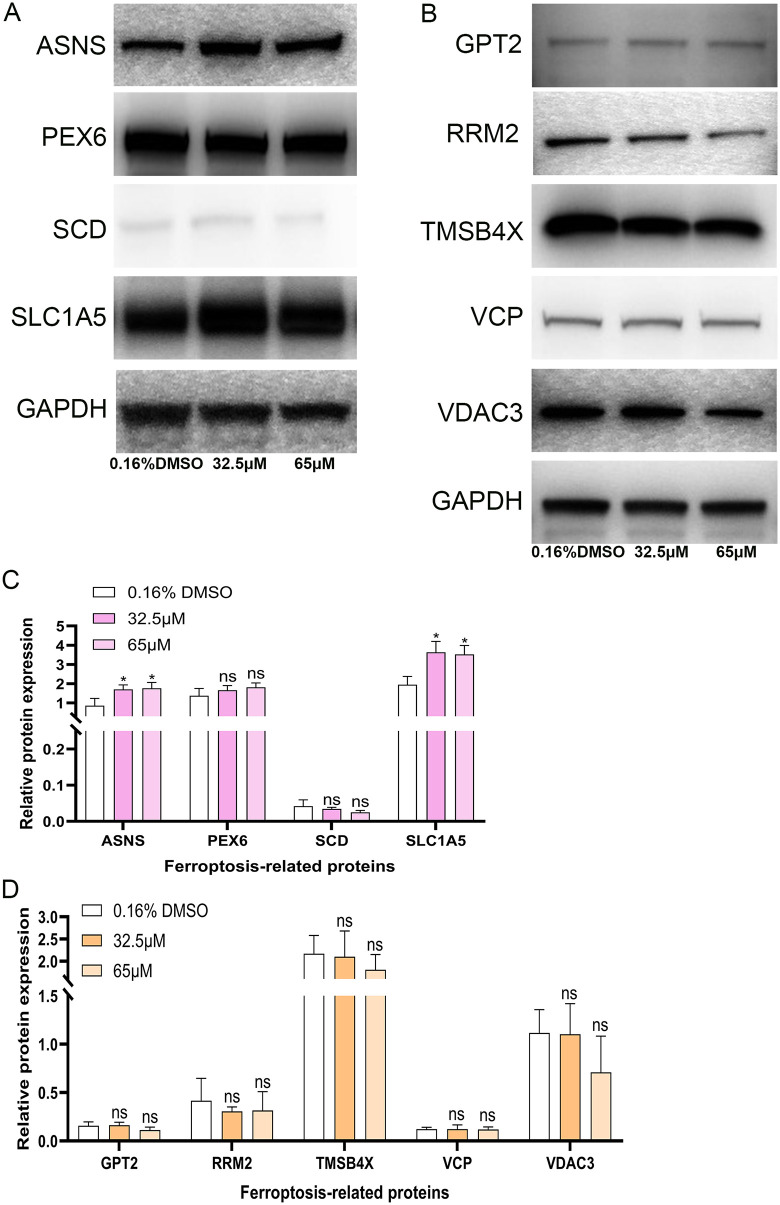
Expression levels of ferroptosis-related proteins in western blot assays. A-B. Western blot images of 9 human ferroptosis-related proteins. The HeLa cells were treated with three concentrations of 6-methoxyflavone (0.16% DMSO, 32.5 μM, and 65 μM) for 48 hours. All assays were repeated at least thrice. C-D. The histogram shows the relative protein expression levels in Fig 4A-B. Statistical analysis was performed using a one-way analysis of variance (ANOVA). *p < 0.05. ns: not significant.

**Fig 5:**
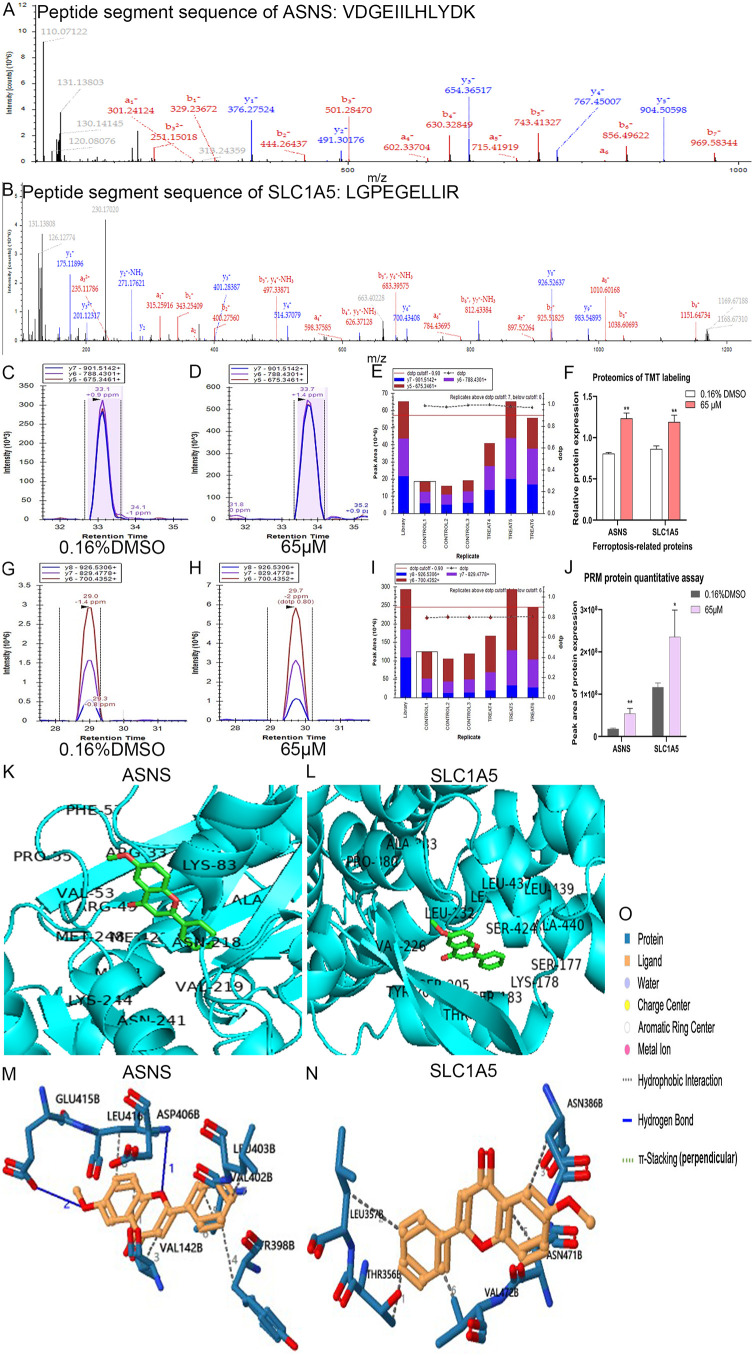
Expression, affinity, and non-covalent interactions analyses of ferroptosis-related proteins. A-J. Expression levels of ferroptosis-related proteins in TMT and PRM proteomics. The HeLa cells were treated with two concentrations of 6-methoxyflavone (0.16% DMSO and 65 μM) for 48 hours. All assays were repeated at least thrice. Statistical analysis was performed using t-tests. *p < 0.05. **p < 0.01. ns: not significant. ASNS: Asparagine synthetase (glutamine-hydrolyzing). SLC1A5: Solute carrier family 1 member 5. A-B. The mass spectrogram of peptide segments of ASNS and SLC1A5 in the TMT proteome. The peptide segment amino acid sequence of ASNS and SLC1A5 were VDGEIILHLYDK and LGPEGELLIR. **F.** The relative protein expression levels of ASNS and SLC1A5 in the TMT proteome. C-E, G-J. Expression levels of ASNS and SLC1A5 in PRM proteomics. C-D, G-H. Peak area chromatograms in Skyline software. E, **I.** Peak area histograms in Skyline software. **J.** The relative protein expression levels of ASNS and SLC1A5 in the PRM proteome. K-O. Analyses of affinity and non-covalent interactions between 6-methoxyflavone and the two target proteins. K–L: Molecular docking results. M–N: Non-covalent interactions. O: Annotations for M–N.

A list of 471 human ferroptosis-related genes was downloaded from FerrDB. We overlapped the 526 significantly differentially expressed proteins with the 471 encoded proteins of human ferroptosis-related genes, resulting in 32 significantly differentially expressed human ferroptosis-related proteins (**Fig 2K**). We extracted the expression profile data of these 32 proteins from the TMT proteome (**Table 5**, Fig 3A-3D). Moreover, another list of 11 ferroptosis-related genes was downloaded from the KEGG official website (**Fig 2K**). After treatment with 32.5 μM of 6-methoxyflavone, we performed qPCR analyses on these 43 genes. The qPCR results indicated that 22 out of 43 genes exhibited significant differential expression at the mRNA level (**Fig 2K**, **Fig 3E-3I**). The expression trends of 16 out of these 22 genes were consistent with their expression in the TMT proteome (**Table 5**). The expression trends of 9 out of the 16 genes, as measured by TMT or qPCR, aligned with the data in the FerrDb database (**Table 5**). After treating with 32.5 μM and 65 μM of 6-methoxyflavone, we conducted western blot analyses on the proteins encoded by these nine genes. These nine genes were ASNS, GPT2, PEX6, RRM2, SCD, SLC1A5, TMSB4X, VCP, and VDAC3 (**Fig 2K**). After treatment with 32.5 μM and 65 μM of 6-methoxyflavone, the western blot results revealed significant differential expression in only two proteins at both concentrations. These two significantly differentially expressed proteins were ASNS and SLC1A5 (**Fig 2K**, **Fig 4**). We extracted mass spectrograms and differentially expressed peptide sequences of ASNS and SLC1A5 from the TMT proteome (Fig 5A-5B). The results of TMT proteome showed that 6-methoxyflavone significantly upregulated ASNS and SLC1A5 expression (**Fig 5F**).

**Table 5:** Expression trends at mRNA and protein levels.

Targets	TMT Fold Change	TMT p value	qPCR Fold Change	qPCR p value	Expression trends at mRNA and protein levels	TMT/qPCR Expression trends vs FerrDb database
ASNS	1.53	＜0.01	8.03	＜0.01	consistency	consistency
FADS1	0.72	＜0.01	0.59	＜0.01	consistency	inconsistency
GDF15	1.27	＜0.01	12.88	＜0.01	consistency	inconsistency
GPT2	1.3	0.01	2.59	0.02	consistency	consistency
HMGB1	0.77	0.03	0.72	0.04	consistency	inconsistency
PEX6	1.21	0.01	2.75	0.02	consistency	consistency
PLIN2	1.62	＜0.01	1.80	＜0.01	consistency	inconsistency
RRM2	0.79	0.03	0.81	＜0.01	consistency	consistency
SCD	0.8	＜0.01	0.72	＜0.01	consistency	consistency
SLC1A5	1.38	＜0.01	1.66	0.02	consistency	consistency
SNCA	0.79	＜0.01	0.54	＜0.01	consistency	inconsistency
TMSB4X	0.59	＜0.01	0.69	0.02	consistency	consistency
VCP	0.75	0.04	0.61	0.03	consistency	consistency
GCLC	——	——	2.93	＜0.01	——	inconsistency
TFRC	——	——	0.77	0.03	——	inconsistency
VDAC3	——	——	0.74	＜0.01	——	consistency
AURKA	1.22	0.02	0.70	0.03	inconsistency	——
CAV1	1.28	＜0.01	0.63	＜0.01	inconsistency	——
CIRBP	0.82	0.02	1.21	0.04	inconsistency	——
CP	1.38	＜0.01	0.24	0.02	inconsistency	——
GOT1	0.80	0.03	1.26	＜0.01	inconsistency	——
MMP13	1.39	0.02	0.43	0.01	inconsistency	——

### 3.5 6-Methoxyflavone upregulates ASNS and SLC1A5 expression

After treatment with 65 μM of 6-methoxyflavone, we conducted PRM proteomic analyses (**S5 File**) on ASNS (**Fig 5C-E**) and SLC1A5 (**Fig 5G-I**) using Skyline software. The results of PRM proteome showed that 6-methoxyflavone significantly upregulated ASNS and SLC1A5 expression (**Fig 5J**).

### 3.6 6-Methoxyflavone alters the functional domains of ASNS and SLC1A5

After treatment with 65 μM of 6-methoxyflavone, 6-methoxyflavone significantly upregulated ASNS and SLC1A5 expression. 6-Methoxyflavone altered the functional domains of ASNS and SLC1A5 (Table 6). The peptide segment amino acid sequence of ASNS and SLC1A5 were VDGEIILHLYDK and LGPEGELLIR in TMT and PRM proteome, respectively (Fig 5A-5J, Table 6).

**Table 6:** Functional domain and peptide segment sequence analyses of ferroptosis-related proteins.

Protein	Gene	Domain	Name	Start_ location	Stop_ location	Amino acid sequence of peptide segment
P08243	ASNS	PF13537	Glutamine amidotransferase domain	49	164	VDGEIILHLYDK
P08243	ASNS	PF00733	Asparagine synthase	235	385	VDGEIILHLYDK
Q15758	SLC1A5	PF00375	Sodium:dicarboxylate symporter family	54	483	LGPEGELLIR

### 3.7 6-Methoxyflavone alters the structure of ferroptosis-related genes

After treatment with 65 μM of 6-methoxyflavone, the alternative splicing types of AE and TSS in SLC1A5 were altered. ASNS exhibited the most significant changes in alternative splicing types and sites (**Table 7, S6 File**). After treatment with 65 μM of 6-methoxyflavone, ASNS and SLC1A5 each produced a new transcript (**Table 8, S7 File**).

**Table 7:** Alternative splicing analyses of ferroptosis-related genes.

Group	AE	MSKIP	SKIP	TSS	TTS	XMSKIP	XSKIP
ASNS_control	10	18	14	14	5	6	4
ASNS_treat	12	28	18	18	3	10	6
SLC1A5_control	2	0	0	18	3	0	12
SLC1A5_treat	0	0	0	17	3	0	12

Abbreviations: AE, Alternative exon ends; TSS, Transcription start site; TTS, transcription terminal site; SKIP, Skipped exon; XSKIP, Approximate SKIP; MSKIP, Multi-exon SKIP; XMSKIP, Approximate MSKIP.

**Table 8:** New transcript analyses of ferroptosis-related genes.

Gene	Isoform ID	Postion	Class Code	Compare Ref
ASNS	MSTRG.11960.3	7 [-]97852131-97872165	j	ENST00000175506
SLC1A5	MSTRG.6946.4	19 [-]46777211-46788594	j	ENST00000542575

### 3.8 6-Methoxyflavone has the strongest affinity for SLC1A5

6-Methoxyflavone had the strongest affinity for SLC1A5 (**Fig 5L**; **Table 9**). The free energy of ASNS and SLC1A5 were −6.9 and −8.4, respectively (Fig 5K-5L; **Table 9**). The primary types of non-covalent interactions between 6-methoxyflavone and the two target proteins were hydrophobic interaction and hydrogen bond (Fig 5M-5N; **Table 9**). These results indicated that 6-methoxyflavone has affinities for two ferroptosis-related proteins, all of which have non-covalent interaction sites (Fig 5K-5O; **Table 9**). The core gene responsible for inducing ferroptosis in HeLa cells by 6-methoxyflavone was SLC1A5.

**Table 9:** Molecular docking and non-covalent interaction.

Targets	Ligand	Free energy (kcal/M)	Ki/Kd (μM)	Interactions
SLC1A5	6MF	−8.40	0.69	Hydrophobic Interaction
ASNS	6MF	−6.90	8.64	Hydrophobic Interaction Hydrogen Bond

Abbreviations: 6MF: 6-Methoxyflavone, Ki: inhibition constant, Kd: dissociation constant.

### 3.9 The functional roles of ASNS and SLC1A5 in 6-methoxyflavone-induced ferroptosis

**Fig 6:**
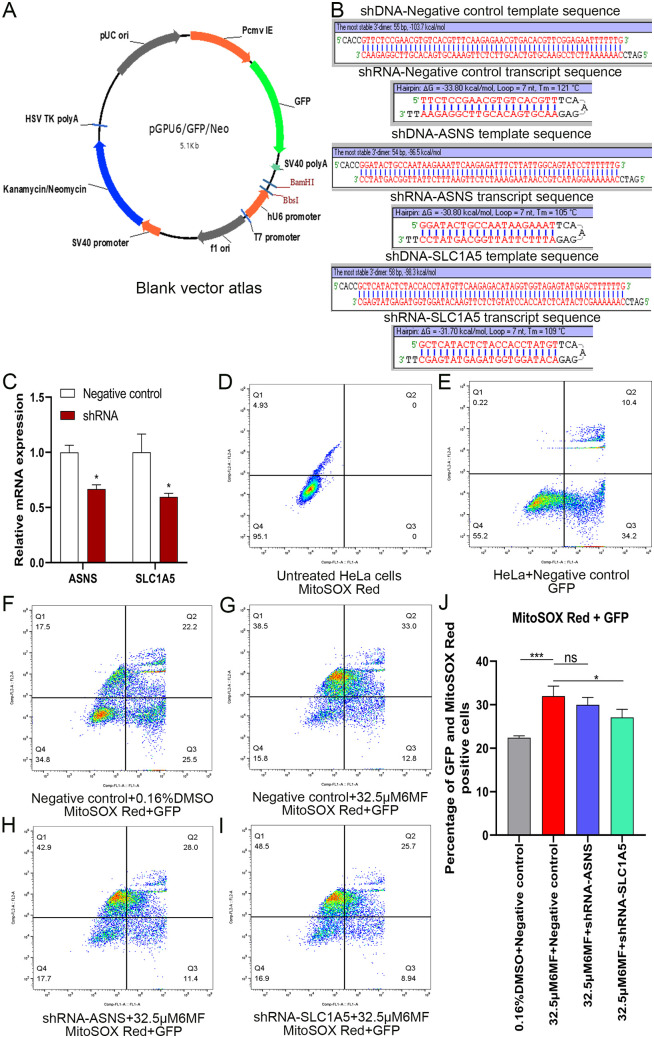
Plasmid construction, cell transfection, and mitochondrial superoxide assay. All assays were repeated at least thrice. *p < 0.05. **p < 0.01. ns: not significant. **A.** The pGPU6/GFP/Neo blank vector atlas. The vector contained the green fluorescent protein (GFP). **B.** The short hairpin RNA (shRNA) interference plasmid sequences. ASNS, SLC1A5, and negative control shRNA interference plasmids were obtained from Genepharma and transfected into HeLa cells using Lipo8000 reagent. **C.** The HeLa cells was digested and collected for quantitative polymerase chain reaction (qPCR) analysis to detect transfection efficiency. Statistical analysis was performed using paired t-tests. D-J. Cell transfection and mitochondrial superoxide assay. D-I. Flow cytometry images. The transfected cells were treated with 0.16% DMSO and 32.5 μM 6-methoxyflavone for 24 hours. A flow cytometer was used to detect the fluorescence intensity in the FL1 and FL2 channels. Q1: GFP(-) and MitoSOX Red(+). Q2: GFP(+) and MitoSOX Red(+). Q3: GFP(+) and MitoSOX Red(-). Q4: GFP(-) and MitoSOX Red(-). D-E. The assay included two control groups: MitoSOX Red single fluorescence group of untreated HeLa cells, and GFP single fluorescence group of HeLa cells transfected with negative control shRNA. **F.** Treatment with Negative control and 0.16%DMSO. **G.** Treatment with Negative control and 32.5 μM 6-methoxyflavone. **H.** Treatment with shRNA-ASNS and 32.5 μM 6-methoxyflavone. **I.** Treatment with shRNA-SLC1A5 and 32.5 μM 6-methoxyflavone. **J.** The histogram shows the percentage of GFP and MitoSOX Red positive cells in Q2 of Fig 6D-6I. Statistical analysis was performed using a one-way analysis of variance (ANOVA). A mitochondrial superoxide assay and a flow cytometer were used to detect the fluorescence intensity.

In summary, we found that ASNS and SLC1A5 was the important gene biomarkers of 6-methoxyflavone-induced ferroptosis in HeLa cells. To determine the specific functional roles of ASNS and SLC1A5, we performed loss-of-function genetic manipulation and mitochondrial superoxide assays. Fig 6A shows the pGPU6/GFP/Neo blank vector atlas. The shRNA sequences are listed in Fig 6B. The results of qPCR showed that the shRNA interference plasmids of ASNS and SLC1A5 were successfully constructed (Fig 6C). The intervention efficiency of ASNS and SLC1A5 met the requirements of genetic manipulation. Fig 6D shows untreated HeLa cells stained with MitoSOX Red. Fig 6E displays HeLa cells with GFP fluorescence after transfection with a negative control plasmid. The results of mitochondrial superoxide assays showed that 6-methoxyflavone significantly upregulated superoxide levels in HeLa cell mitochondria (Fig 6F, 6G, 6J). The ASNS shRNA interference plasmid downregulated the mitochondrial superoxide levels upregulated by 6-methoxyflavone, but the difference was not significant (Fig 6H, 6J). The SLC1A5 shRNA interference plasmid significantly downregulated the mitochondrial superoxide levels upregulated by 6-methoxyflavone (Fig 6I, 6J). The core gene responsible for the induction of ferroptosis and the upregulation of mitochondrial superoxide in HeLa cells by 6-methoxyflavone was identified as SLC1A5.

## 4 Discussion

Induction of cancer cell ferroptosis is a promising cancer treatment strategy [5]. Ferroptosis is a potential target for cervical cancer treatment. This study aimed to elucidate the role and molecular mechanism of 6-methoxyflavone in inducing ferroptosis in HeLa cells, offering novel insights for potential therapeutic strategies in the management of cervical cancer. After treatment with 6-methoxyflavone, ferroptosis morphological changes at various stages were observed in HeLa cells, such as cytoplasmic membrane rupture, mitochondrial wrinkling, increased mitochondrial membrane density, reduced mitochondrial volume, and decreased or disappeared mitochondrial cristae. These TEM images indicated that 6-methoxyflavone markedly induced ferroptosis in HeLa cells. This effect was further validated through mitochondrial superoxide assay, and assessment of reduced and oxidized glutathione content.

Ferroptosis is closely associated with cancer metabolism [16] and affects cancer occurrence and progression. In our study, non-targeted metabolomics and targeted medical metabolomics indicated that 6-methoxyflavone was significantly correlated with ferroptosis. 6-Methoxyflavone significantly upregulated arachidonic acid and adrenic acid expression, whereas it significantly downregulated glutathione (reduced), glutathione (oxidized), glutamate, cysteine, and glutamine expression. The above metabolites are closely related to ferroptosis.

Transmission electron microscopy imaging, mitochondrion superoxide assay, GSH and GSSG content assay, non-targeted and targeted metabolomic analyses all indicated 6-Methoxyflavone induced ferroptosis in HeLa cells. Therefore, we further to explore the molecular mechanism of 6-methoxyflavone induced ferroptosis in HeLa cells. TMT proteomics, qPCR, western blot, PRM proteomics, alternative splicing, new transcript, functional domain, molecular docking, and non-covalent interaction analyses indicated that ASNS and SLC1A5 was the important gene biomarkers of 6-methoxyflavone-induced ferroptosis in HeLa cells. ASNS and SLC1A5 are associated with ferroptosis [21]. ASNS catalyzes asparagine synthesis from aspartate, and ASNS downregulation reprograms amino acid metabolism [39]. Under normal conditions, glutamine is essential for cell survival and growth. However, growth and survival factors induce ferroptosis during amino acid starvation. Supplementing glutamine during amino acid starvation increases intracellular reactive oxygen species production and induces ferroptosis in PC3 cells [40]. SLC1A5 overexpression induces glutamine uptake and decomposition, regulates alpha-ketoglutarate expression, leads to reactive oxygen species accumulation in the mitochondria, and induces ferroptosis [41].

To determine the specific functional roles of ASNS and SLC1A5, we performed loss-of-function genetic manipulation and mitochondrial superoxide assays. The SLC1A5 shRNA interference plasmid significantly downregulated the mitochondrial superoxide levels upregulated by 6-methoxyflavone. The core gene responsible for the induction of ferroptosis and the upregulation of mitochondrial superoxide in HeLa cells by 6-methoxyflavone was identified as SLC1A5. TMT and PRM proteomics analyses indicated that 6-methoxyflavone specifically interacted with the peptide sequence LGPEGELLIR of SLC1A5. Molecular docking analysis further confirmed a high binding affinity between 6-methoxyflavone and SLC1A5. Additionally, non-covalent interaction analysis suggested that the interaction between 6-methoxyflavone and SLC1A5 was primarily driven by hydrophobic interactions.

Ferroptosis is closely associated with tumor progression; therefore, inhibiting ferroptosis can promote tumor progression [42]. In this study, 6-methoxyflavone induced ferroptosis in HeLa cells by markedlyaltering the expression of ferroptosis-related genes, proteins, and metabolites, thereby exerting anticancer effects. The core gene responsible for inducing ferroptosis was SLC1A5. However, this study was limited to cancer cells and did not include in vivo validation using animal models or patient samples. Additionally, this study assessed ferroptosis at 24 and 48 hours post-treatment. It would be valuable to evaluate the kinetics of ferroptosis over a broader time range, such as at 6 or 12 hours. This approach would provide a better understanding of the process dynamics and the rate at which ferroptosis is induced. Furthermore, the study does not include ferroptosis inhibitors to verify the specificity of 6-methoxyflavone’s effect on ferroptosis. Incorporating these inhibitors would provide stronger evidence that 6-methoxyflavone induces ferroptosis rather than other forms of cell death. Future studies will incorporate additional animal models, a larger number of patient tissue samples, a wider range of time points, and assays involving ferroptosis inhibitors.

## 5 Conclusion

6-Methoxyflavone induces ferroptosis in HeLa cells by markedly altering ferroptosis-related genes, proteins, and metabolites expression, thus exerting anticancer effects. Molecular docking analyses showed that 6-methoxyflavone had the strongest affinity for SLC1A5. 6-Methoxyflavone induced ferroptosis in HeLa cells through the upregulation of SLC1A5 expression. The core gene responsible for the induction of ferroptosis and the upregulation of mitochondrial superoxide in HeLa cells by 6-methoxyflavone was identified as SLC1A5. TMT and PRM proteomics revealed that 6-methoxyflavone targeted the peptide segment sequence LGPEGELLIR of SLC1A5.

## Supporting information

S1 File Expression levels of reduced glutathione, glutamate, arachidonic acid, and DL-glutamine in non-targeted metabolomics (negative ion mode). **Page 1.** The chromatogram and mass spectrogram of glutathione (reduced) in non-targeted metabolomics (negative ion mode) in the control group (0.16% DMSO). **Page 2.** The chromatogram and mass spectrogram of glutathione (reduced) in non-targeted metabolomics (negative ion mode) in the treat group (65 μM). **Page 3.** The chromatogram and mass spectrogram of glutamate in non-targeted metabolomics (negative ion mode) in the control group (0.16% DMSO). **Page 4.** The chromatogram and mass spectrogram of glutamate in non-targeted metabolomics (negative ion mode) in the treat group (65 μM). **Page 5.** The chromatogram and mass spectrogram of arachidonic acid in non-targeted metabolomics (negative ion mode) in the control group (0.16% DMSO). **Page 6.** The chromatogram and mass spectrogram of arachidonic acid in non-targeted metabolomics (negative ion mode) in the treat group (65 μM). **Page 7.** The chromatogram and mass spectrogram of DL-glutamine in non-targeted metabolomics (negative ion mode) in the control group (0.16% DMSO). **Page 8.** The chromatogram and mass spectrogram of DL-glutamine in non-targeted metabolomics (negative ion mode) in the treat group (65 μM).
(PDF)

S2 File Expression levels of reduced glutathione, oxidized glutathione, glutamate, and D-glutamine in non-targeted metabolomics (positive ion mode). **Page 1.** The chromatogram and mass spectrogram of glutathione (reduced) in non-targeted metabolomics (positive ion mode) in the control group (0.16% DMSO). **Page 2.** The chromatogram and mass spectrogram of glutathione (reduced) in non-targeted metabolomics (positive ion mode) in the treat group (65 μM). **Page 3.** The chromatogram and mass spectrogram of glutathione (oxidized) in non-targeted metabolomics (positive ion mode) in the control group (0.16% DMSO). **Page 4.** The chromatogram and mass spectrogram of glutathione (oxidized) in non-targeted metabolomics (positive ion mode) in the treat group (65 μM). **Page 5.** The chromatogram and mass spectrogram of glutamate in non-targeted metabolomics (positive ion mode) in the control group (0.16% DMSO). **Page 6.** The chromatogram and mass spectrogram of glutamate in non-targeted metabolomics (positive ion mode) in the treat group (65 μM). **Page 7.** The chromatogram and mass spectrogram of D-glutamine in non-targeted metabolomics (positive ion mode) in the control group (0.16% DMSO). **Page 8.** The chromatogram and mass spectrogram of D-glutamine in non-targeted metabolomics (positive ion mode) in the treat group (65 μM).
(PDF)

S3 File Expression levels of reduced glutathione in targeted metabolomics. **Page 1.** The chromatogram and mass spectrogram of glutathione (reduced) in targeted metabolomics (amide column) in the first control group (0.16% DMSO). **Page 2.** The chromatogram and mass spectrogram of glutathione (reduced) in targeted metabolomics (amide column) in the second control group (0.16% DMSO). **Page 3.** The chromatogram and mass spectrogram of glutathione (reduced) in targeted metabolomics (amide column) in the third control group (0.16% DMSO). **Page 4.** The chromatogram and mass spectrogram of glutathione (reduced) in targeted metabolomics (amide column) in the fourth control group (0.16% DMSO). **Page 5.** The chromatogram and mass spectrogram of glutathione (reduced) in targeted metabolomics (amide column) in the fifth control group (0.16% DMSO). **Page 6.** The chromatogram and mass spectrogram of glutathione (reduced) in targeted metabolomics (amide column) in the sixth control group (0.16% DMSO). **Page 7.** The chromatogram and mass spectrogram of glutathione (reduced) in targeted metabolomics (amide column) in the first treat group (65 μM). **Page 8.** The chromatogram and mass spectrogram of glutathione (reduced) in targeted metabolomics (amide column) in the second treat group (65 μM). **Page 9.** The chromatogram and mass spectrogram of glutathione (reduced) in targeted metabolomics (amide column) in the third treat group (65 μM). **Page 10.** The chromatogram and mass spectrogram of glutathione (reduced) in targeted metabolomics (amide column) in the fourth treat group (65 μM). **Page 11.** The chromatogram and mass spectrogram of glutathione (reduced) in targeted metabolomics (amide column) in the fifth treat group (65 μM). **Page 12.** The chromatogram and mass spectrogram of glutathione (reduced) in targeted metabolomics (amide column) in the sixth treat group (65 μM).
(PDF)

S4 File Expression levels of glutamate, arachidonic acid, adrenic acid, cystine, and glutamine in targeted metabolomics. **Page 1.** The extracted ion chromatogram and mass spectrogram of glutamate in targeted metabolomics (amide column) in the control group (0.16% DMSO). **Page 2.** The extracted ion chromatogram and mass spectrogram of glutamate in targeted metabolomics (amide column) in the treat group (65 μM). **Page 3.** The extracted ion chromatogram and mass spectrogram of arachidonic acid in targeted metabolomics (C18 column) in the control group (0.16% DMSO). **Page 4.** The extracted ion chromatogram and mass spectrogram of arachidonic acid in targeted metabolomics (C18 column) in the treat group (65 μM). **Page 5.** The extracted ion chromatogram and mass spectrogram of adrenic acid in targeted metabolomics (C18 column) in the control group (0.16% DMSO). **Page 6.** The extracted ion chromatogram and mass spectrogram of adrenic acid in targeted metabolomics (C18 column) in the treat group (65 μM). **Page 7.** The extracted ion chromatogram and mass spectrogram of cystine in targeted metabolomics (amide column) in the control group (0.16% DMSO). **Page 8.** The extracted ion chromatogram and mass spectrogram of cystine in targeted metabolomics (amide column) in the treat group (65 μM). **Page 9.** The extracted ion chromatogram and mass spectrogram of glutamine in targeted metabolomics (amide column) in the control group (0.16% DMSO). **Page 10.** The extracted ion chromatogram and mass spectrogram of glutamine in targeted metabolomics (amide column) in the treat group (65 μM).
(PDF)

S5 File Expression levels of ASNS and SLC1A5 in PRM quantitative detection. **Page 1.** The chromatogram and mass spectrogram of ASNS(VDGEIILHLYDK) in PRM quantitative detection in the control group (0.16% DMSO). **Page 2.** The chromatogram and mass spectrogram of ASNS(VDGEIILHLYDK) in PRM quantitative detection in the treat group (65 μM). **Page 3.** The chromatogram and mass spectrogram of SLC1A5(EVLDSFLDLAR) in PRM quantitative detection in the control group (0.16% DMSO). **Page 4.** The chromatogram and mass spectrogram of SLC1A5(EVLDSFLDLAR) in PRM quantitative detection in the treat group (65 μM).
(PDF)

S6 File Alternative splicing analysis results of ASNS and SLC1A5 for each sample. (XLSX)

S7 File New transcript sequence file. **Page 1.** New transcript sequence file of SLC1A5. **Page 2.** New transcript sequence file of ASNS.
(PDF)

S8 File Supporting Information file. The original, uncropped, and unadjusted images underlying all blot results. **Page 1.** The original, uncropped, and unadjusted blot image of GAPDH in the first batch of samples. **Page 2.** The original, uncropped, and unadjusted blot image of ASNS in the first batch of samples. **Page 3.** The original, uncropped, and unadjusted blot image of PEX6 in the first batch of samples. **Page 4.** The original, uncropped, and unadjusted blot image of SCD in the first batch of samples. **Page 5.** The original, uncropped, and unadjusted blot image of SLC1A5 in the first batch of samples. **Page 6.** The original, uncropped, and unadjusted blot image of GAPDH in the second batch of samples. **Page 7.** The original, uncropped, and unadjusted blot image of GPT2 in the second batch of samples. **Page 8.** The original, uncropped, and unadjusted blot image of RRM2 in the second batch of samples. **Page 9.** The original, uncropped, and unadjusted blot image of TMSB4X in the second batch of samples. **Page 10.** The original, uncropped, and unadjusted blot image of VCP in the second batch of samples. **Page 11.** The original, uncropped, and unadjusted blot image of VDAC3 in the second batch of samples. **Page 12.** Prestained & Western Blot Marker.
(PDF)
