# Supplementary material for: 6-Methoxyflavone targets SLC1A5 to induce ferroptosis in HeLa cells
Source: PLoS One. 2025 Dec 29;20(12):e0339578. doi: 10.1371/journal.pone.0339578 (PMC12747331; doi:10.1371/journal.pone.0339578)

D:\1\control1 02/08/23 09:32:38

1.Glutathione, reduced:Chromatogram and mass spectrogram of non-targeted metabolomics(negative ion mode):control group(0.16%DMSO)

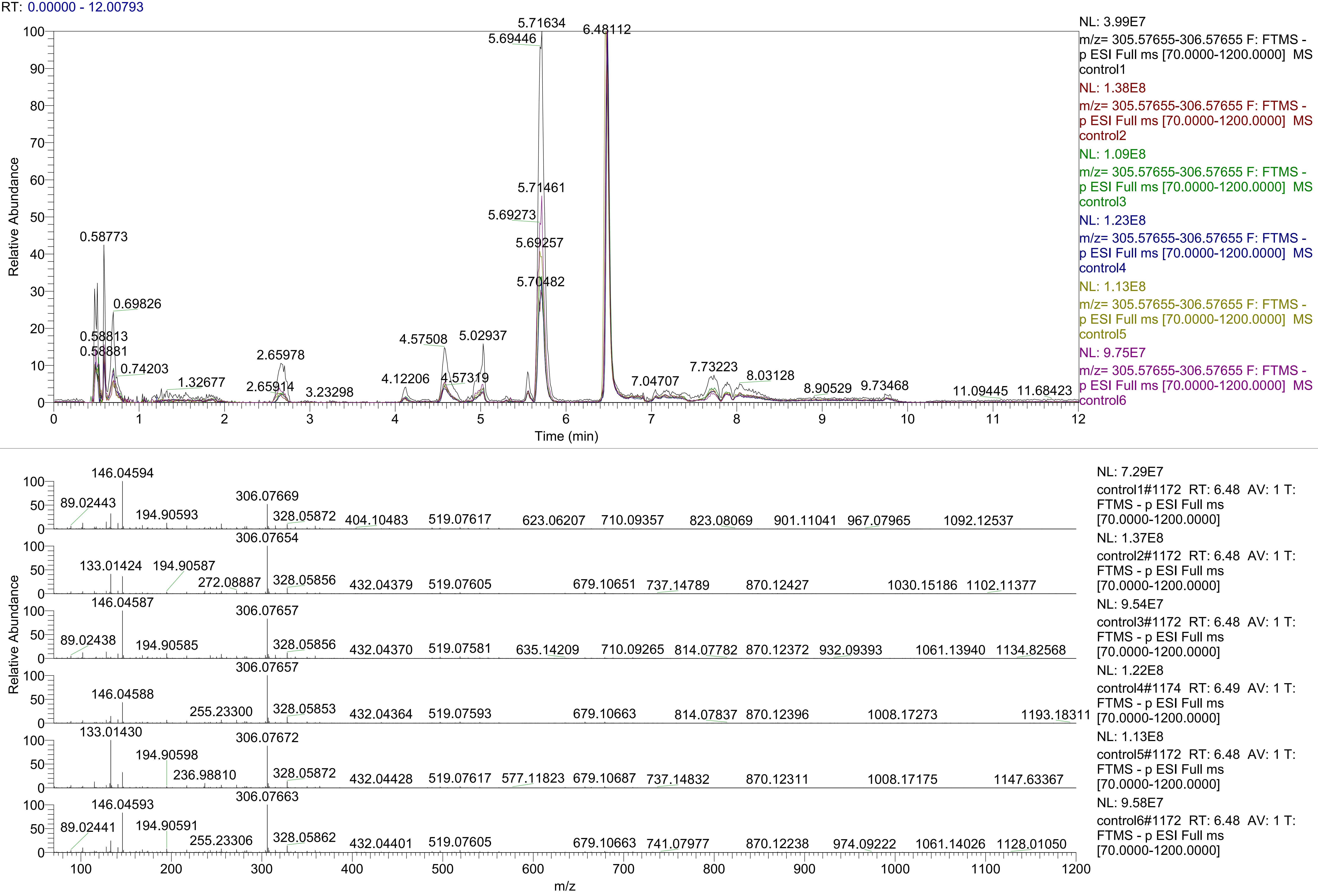

D:\2\Treat7 02/08/23 08:23:07

2.Glutathione, reduced:Chromatogram and mass spectrogram of non-targeted metabolomics(negative ion mode):treat group(65μM)

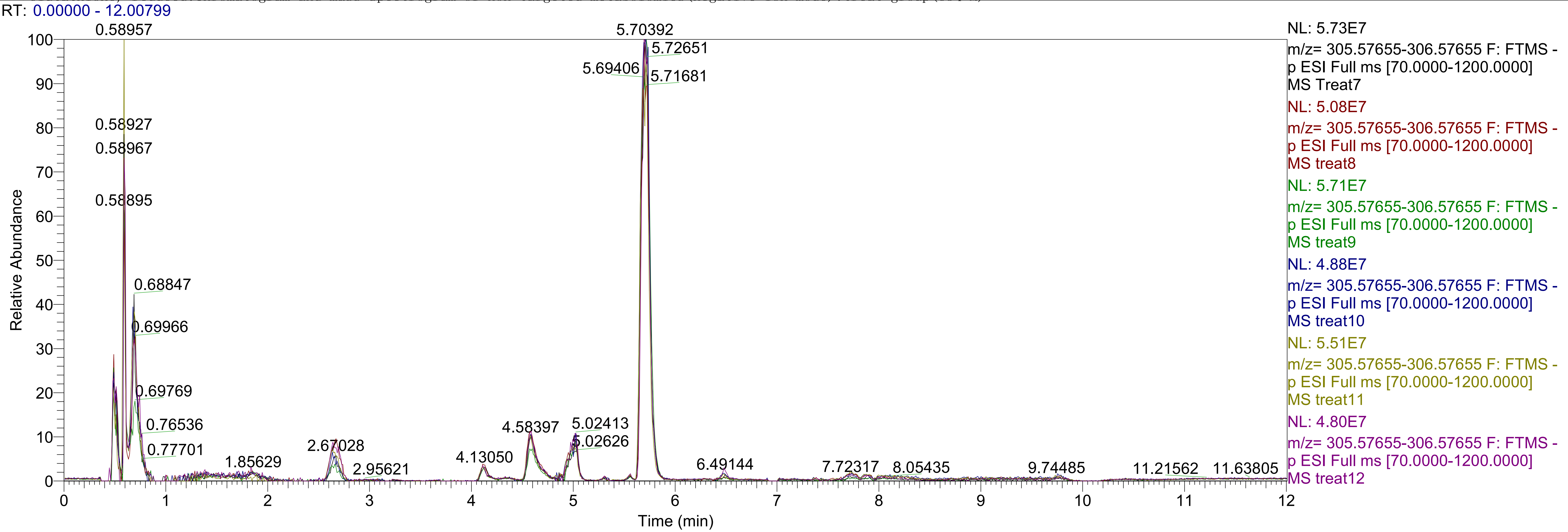

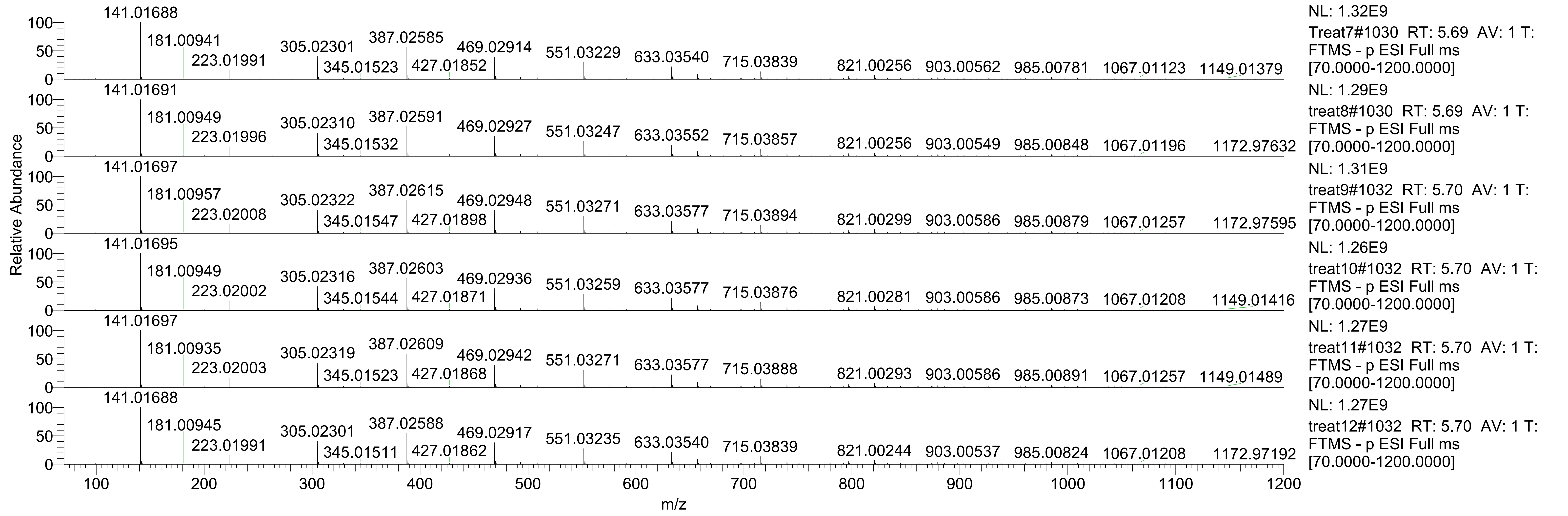

D:\1\control1 02/08/23 09:32:38

3.Glutamate:Chromatogram and mass spectrogram of non-targeted metabolomics(negative ion mode):control group(0.16%DMSO)

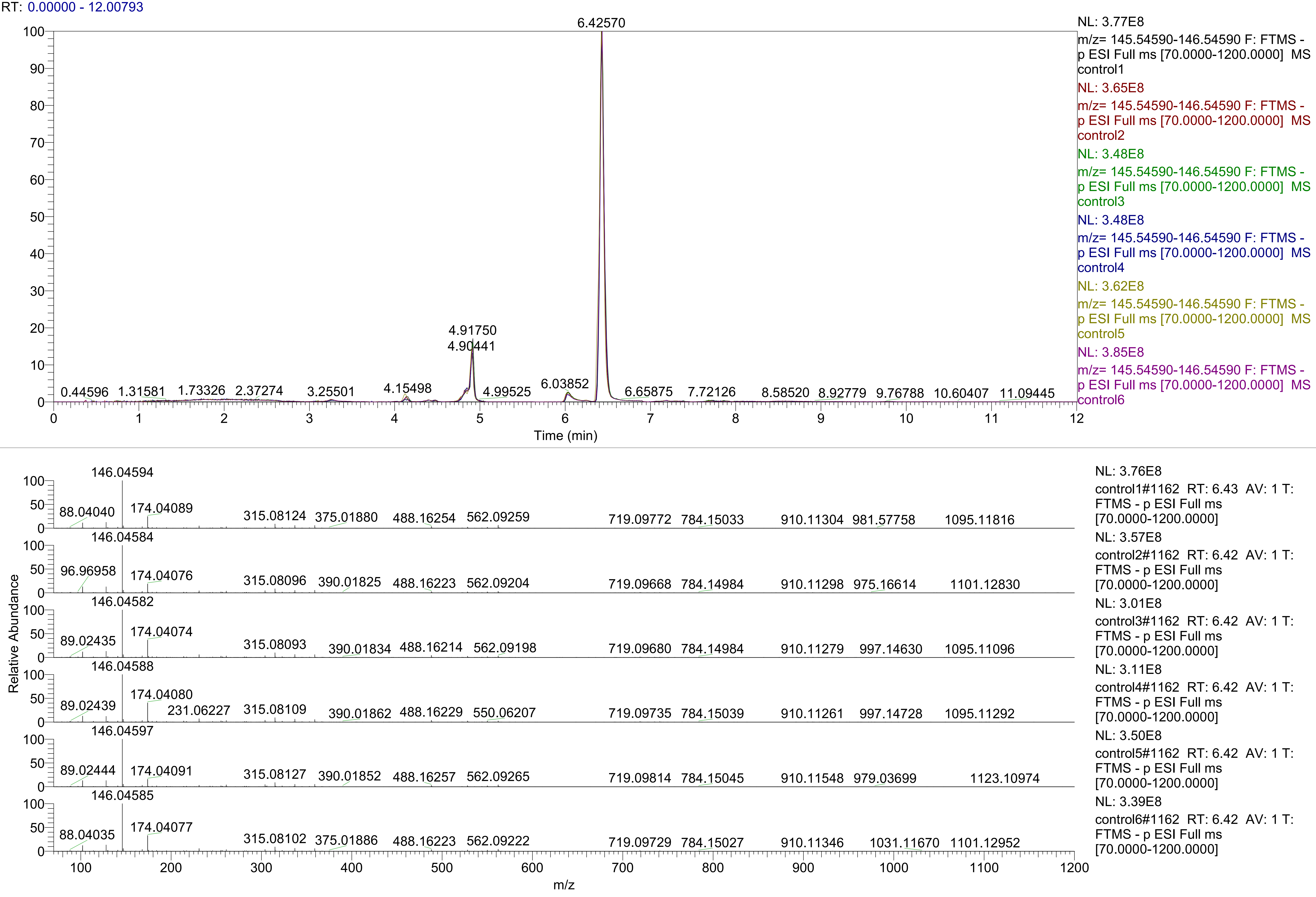

D:\2\Treat7 02/08/23 08:23:07

4.Glutamate:Chromatogram and mass spectrogram of non-targeted metabolomics(negative ion mode):treat group(65μM)

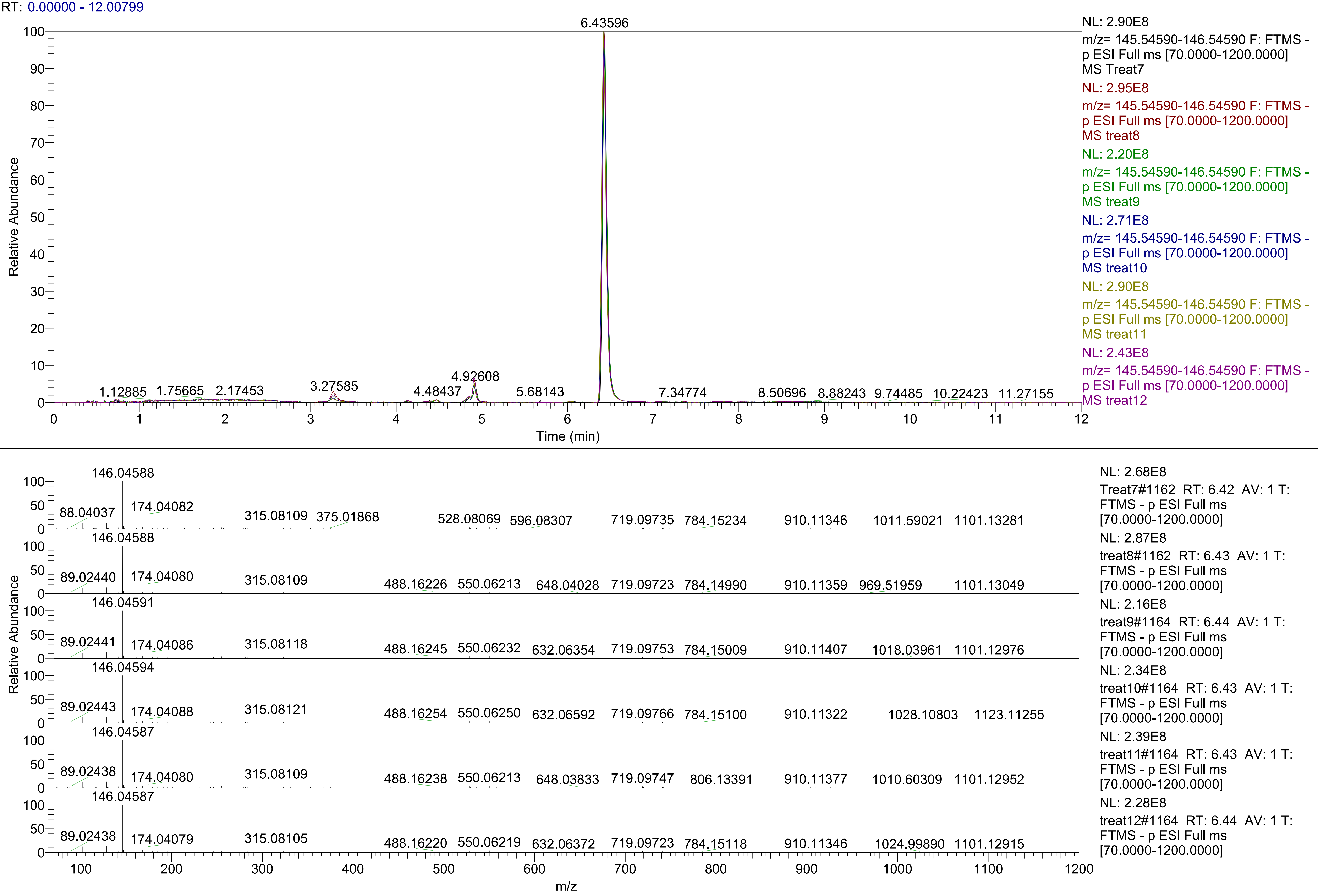

d:\1\control6 02/08/23 10:28:18

5.Arachidonic acid:Chromatogram and mass spectrogram of non-targeted metabolomics(negative ion mode):control group(0.16%DMSO)

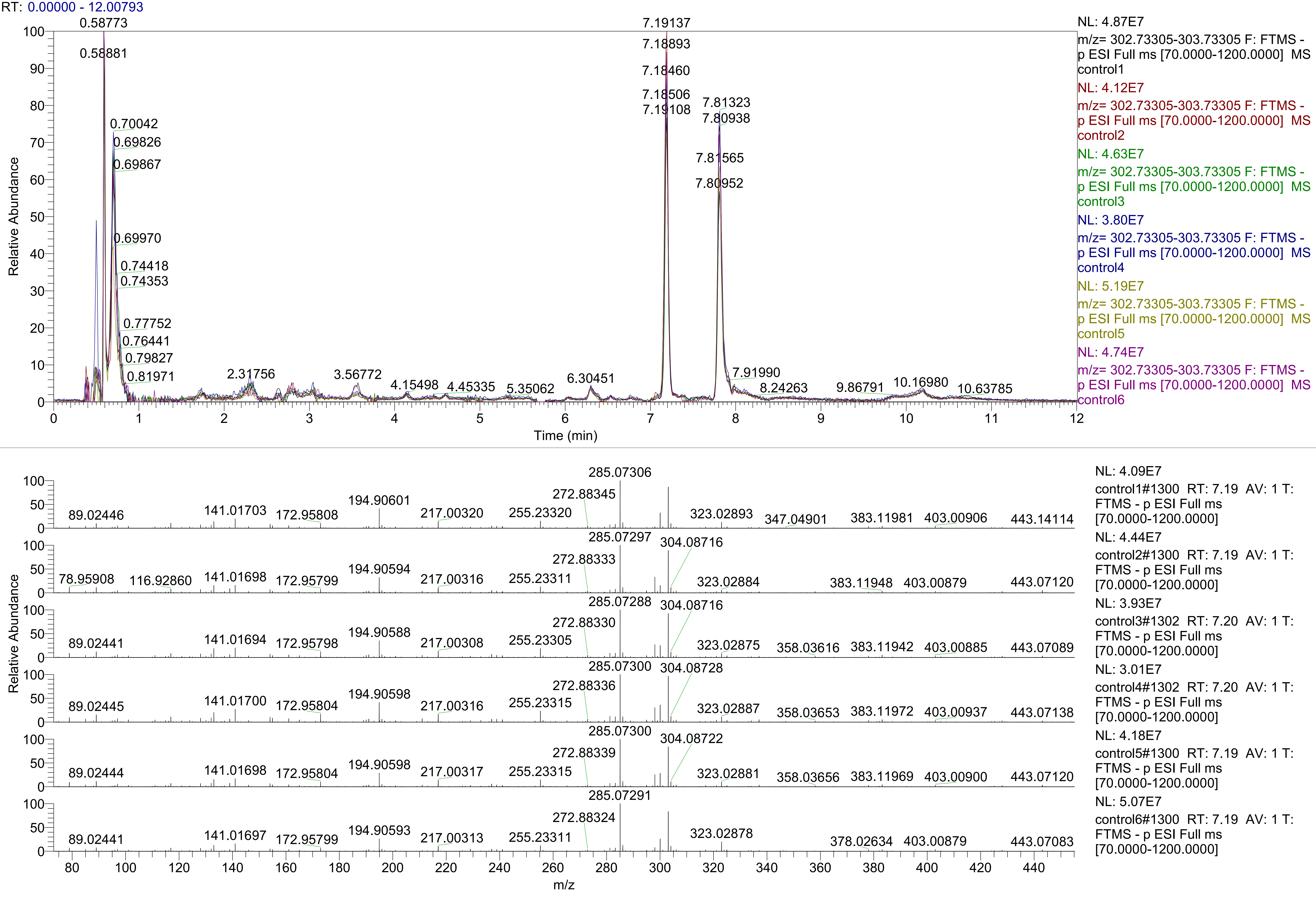

d:\2\treat12 02/08/23 10:14:16

6.Arachidonic acid:Chromatogram and mass spectrogram of non-targeted metabolomics(negative ion mode):treat group(65μM)

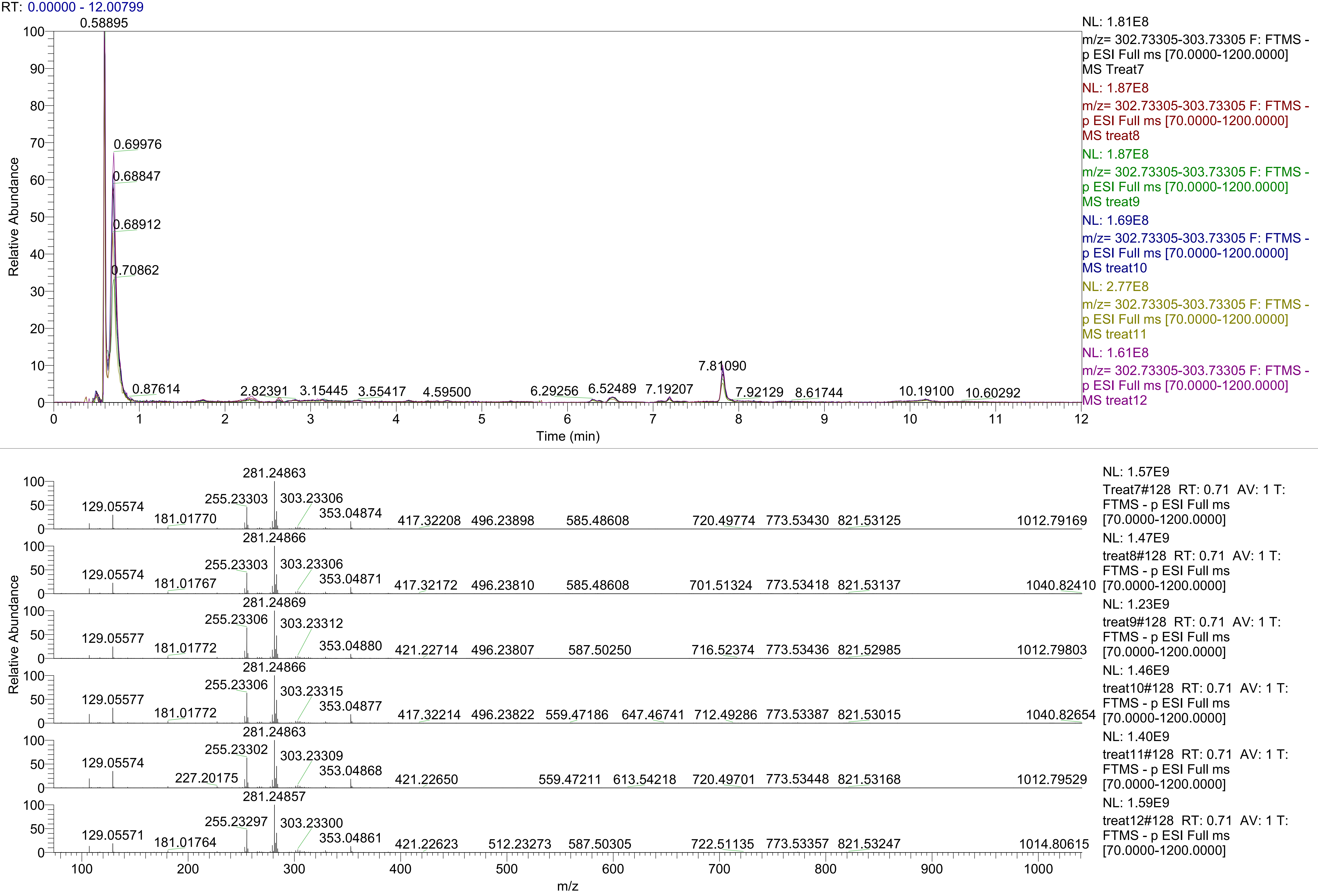

D:\1\control1 02/08/23 09:32:38

7.DL-glutamine:Chromatogram and mass spectrogram of non-targeted metabolomics(negative ion mode):control group(0.16%DMSO)

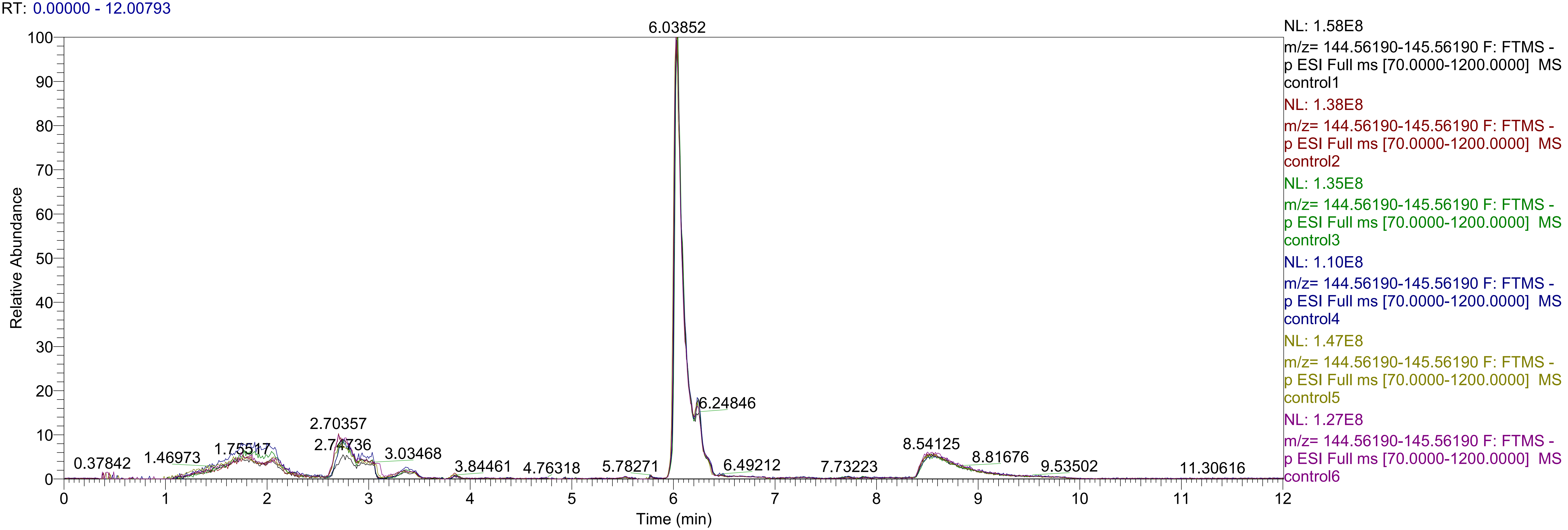

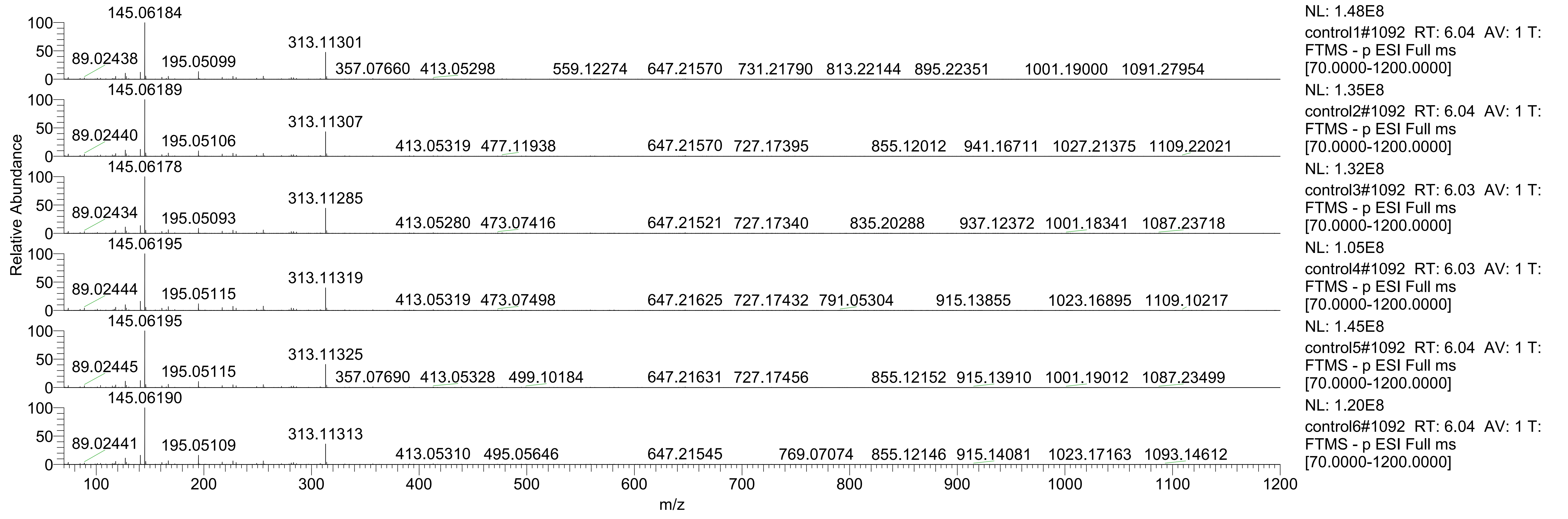

d:\2\treat12 02/08/23 10:14:16

8.DL-glutamine:Chromatogram and mass spectrogram of non-targeted metabolomics(negative ion mode):treat group(65μM)

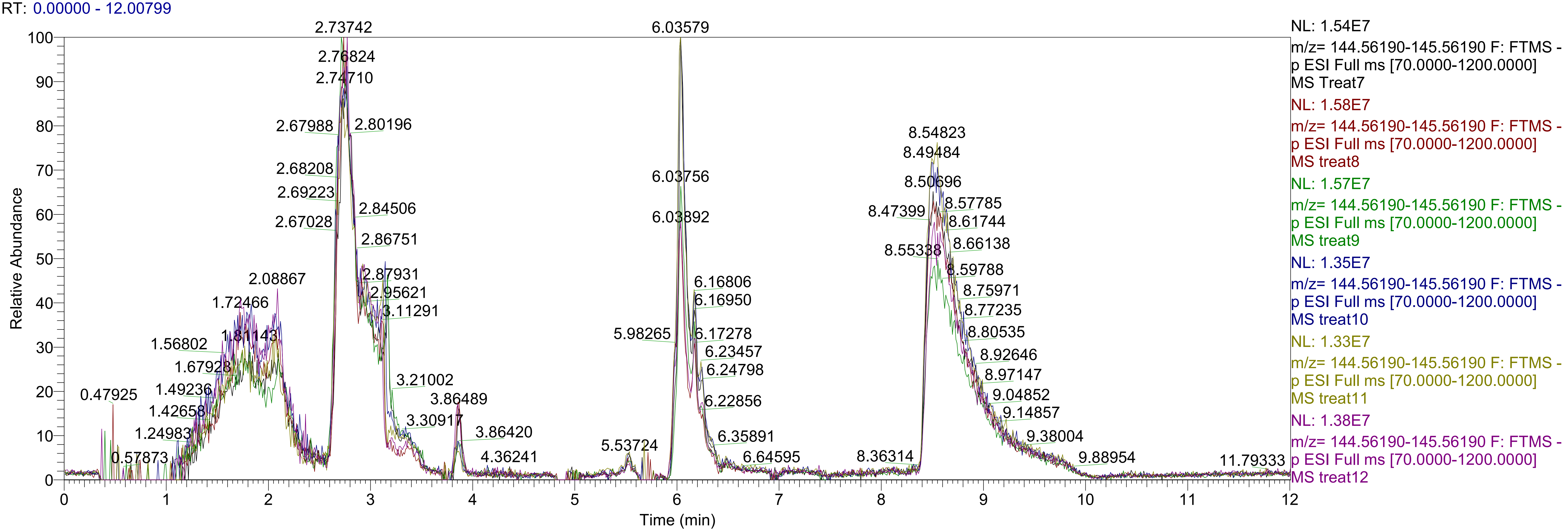

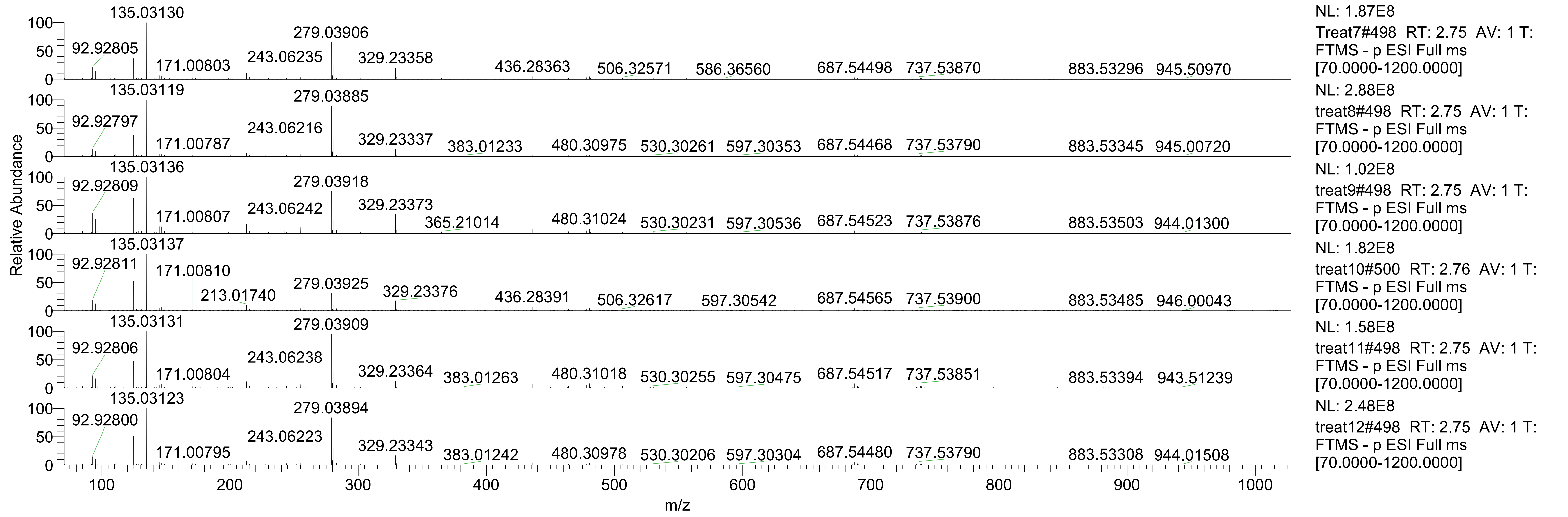

Supplement: S1 File — Page 1. The chromatogram and mass spectrogram of glutathione (reduced) in non-targeted metabolomics (negative ion mode) in the control group (0.16% DMSO). Page 2. The chromatogram and mass spectrogram of glutathione (reduced) in non-targeted metabolomics (negative ion mode) in the treat group (65 μM). Page 3. The chromatogram and mass spectrogram of glutamate in non-targeted metabolomics (negative ion mode) in the control group (0.16% DMSO). Page 4. The chromatogram and mass spectrogram of glutamate in non-targeted metabolomics (negative ion mode) in the treat group (65 μM). Page 5. The chromatogram and mass spectrogram of arachidonic acid in non-targeted metabolomics (negative ion mode) in the control group (0.16% DMSO). Page 6. The chromatogram and mass spectrogram of arachidonic acid in non-targeted metabolomics (negative ion mode) in the treat group (65 μM). Page 7. The chromatogram and mass spectrogram of DL-glutamine in non-targeted metabolomics (negative ion mode) in the control group (0.16% DMSO). Page 8. The chromatogram and mass spectrogram of DL-glutamine in non-targeted metabolomics (negative ion mode) in the treat group (65 μM). (PDF) [file pone.0339578.s001.pdf]
