# Supplementary material for: 6-Methoxyflavone targets SLC1A5 to induce ferroptosis in HeLa cells
Source: PLoS One. 2025 Dec 29;20(12):e0339578. doi: 10.1371/journal.pone.0339578 (PMC12747331; doi:10.1371/journal.pone.0339578)
Supplement: S3 File — Page 1. The chromatogram and mass spectrogram of glutathione (reduced) in targeted metabolomics (amide column) in the first control group (0.16% DMSO). Page 2. The chromatogram and mass spectrogram of glutathione (reduced) in targeted metabolomics (amide column) in the second control group (0.16% DMSO). Page 3. The chromatogram and mass spectrogram of glutathione (reduced) in targeted metabolomics (amide column) in the third control group (0.16% DMSO). Page 4. The chromatogram and mass spectrogram of glutathione (reduced) in targeted metabolomics (amide column) in the fourth control group (0.16% DMSO). Page 5. The chromatogram and mass spectrogram of glutathione (reduced) in targeted metabolomics (amide column) in the fifth control group (0.16% DMSO). Page 6. The chromatogram and mass spectrogram of glutathione (reduced) in targeted metabolomics (amide column) in the sixth control group (0.16% DMSO). Page 7. The chromatogram and mass spectrogram of glutathione (reduced) in targeted metabolomics (amide column) in the first treat group (65 μM). Page 8. The chromatogram and mass spectrogram of glutathione (reduced) in targeted metabolomics (amide column) in the second treat group (65 μM). Page 9. The chromatogram and mass spectrogram of glutathione (reduced) in targeted metabolomics (amide column) in the third treat group (65 μM). Page 10. The chromatogram and mass spectrogram of glutathione (reduced) in targeted metabolomics (amide column) in the fourth treat group (65 μM). Page 11. The chromatogram and mass spectrogram of glutathione (reduced) in targeted metabolomics (amide column) in the fifth treat group (65 μM). Page 12. The chromatogram and mass spectrogram of glutathione (reduced) in targeted metabolomics (amide column) in the sixth treat group (65 μM). (PDF) [file pone.0339578.s003.pdf]

1.Glutathione, reduced: Targeted metabolomics: Chromatogram and mass spectrogram (Amide column) (control1-0.16%DMSO)

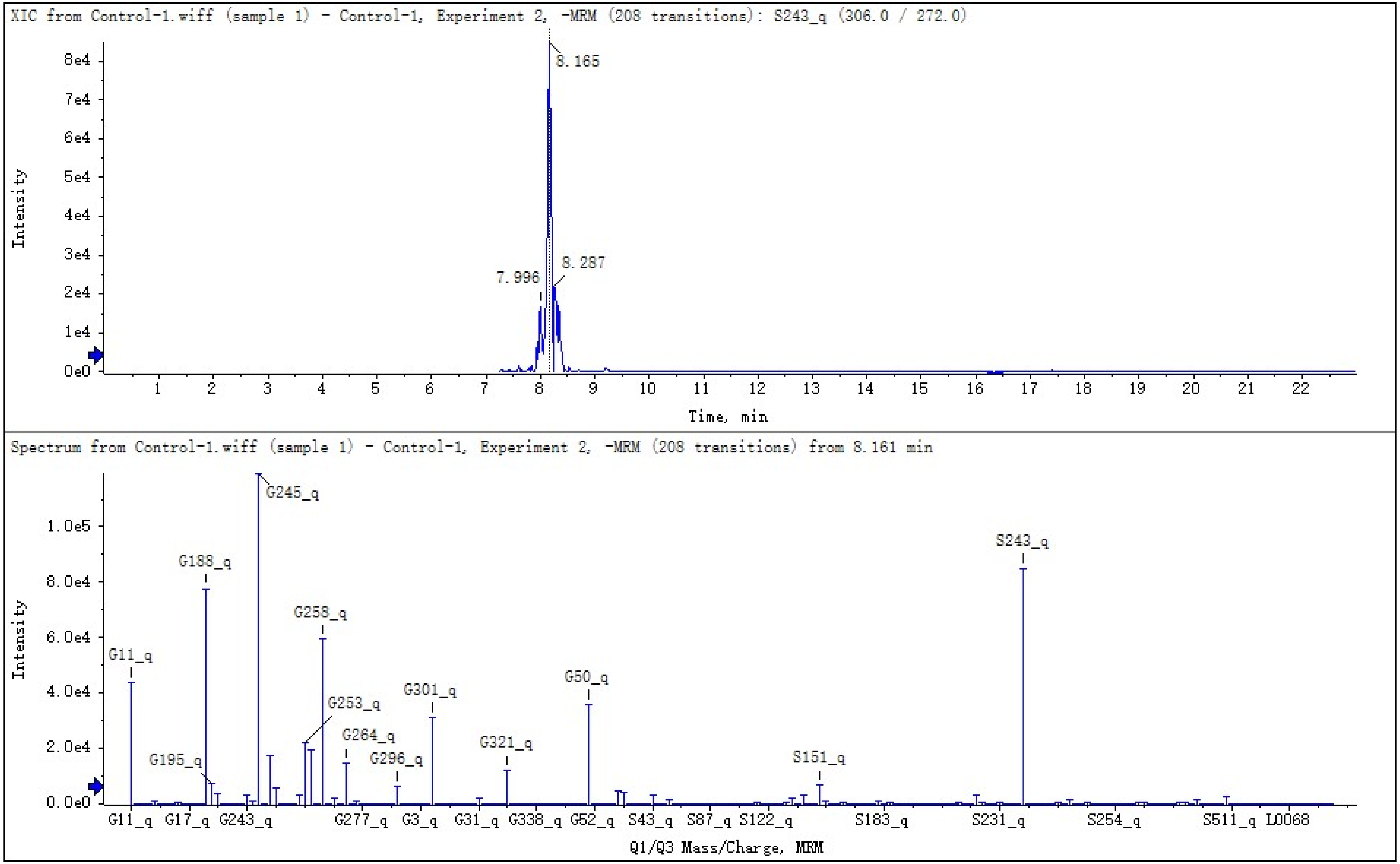

2024/7/18 22:52:30

2.Glutathione, reduced: Targeted metabolomics: Chromatogram and mass spectrogram (Amide column) (control2-0.16%DMSO)

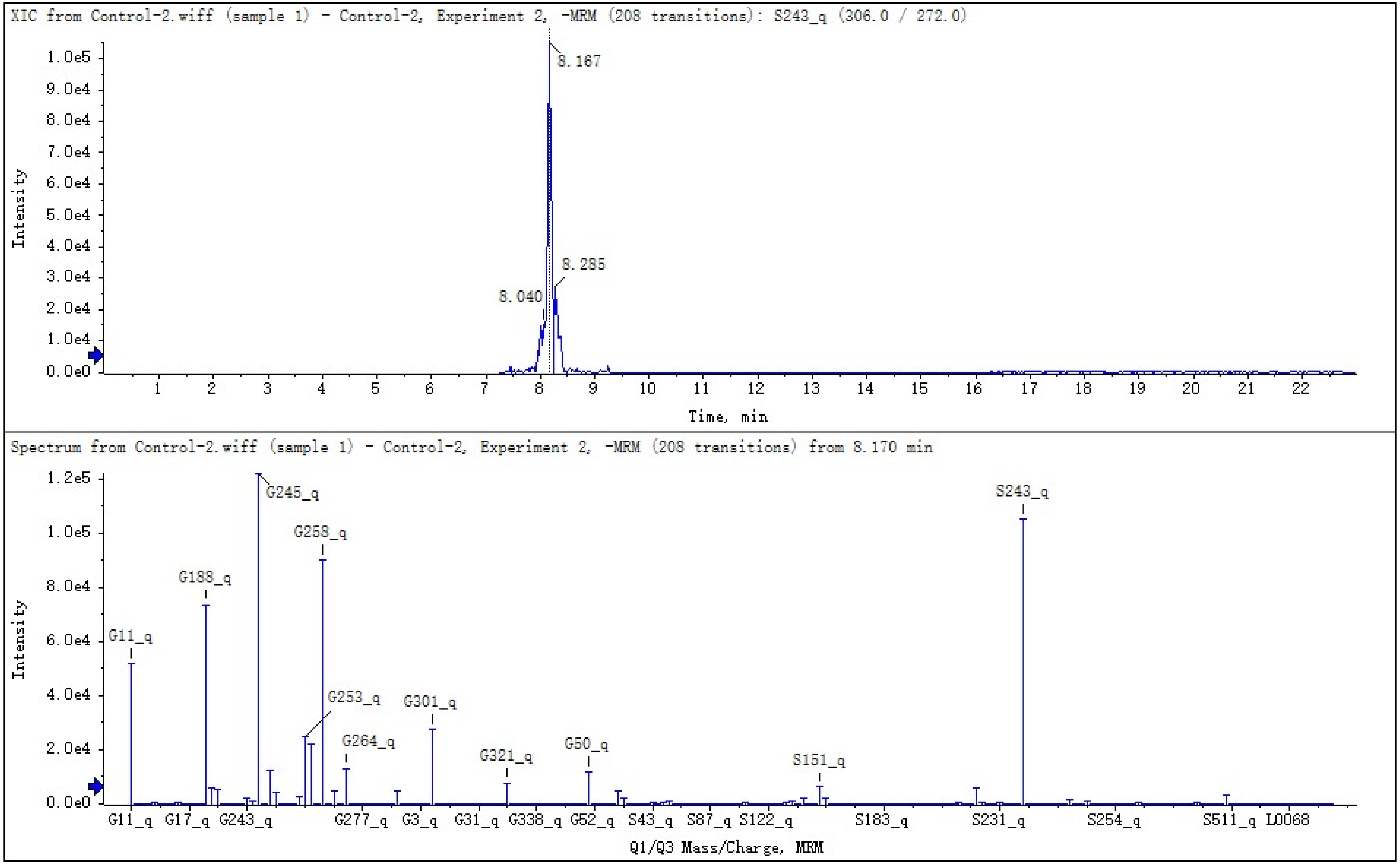

2024/7/18 22:58:22

3.Glutathione, reduced: Targeted metabolomics: Chromatogram and mass spectrogram (Amide column) (control3-0.16%DMSO)

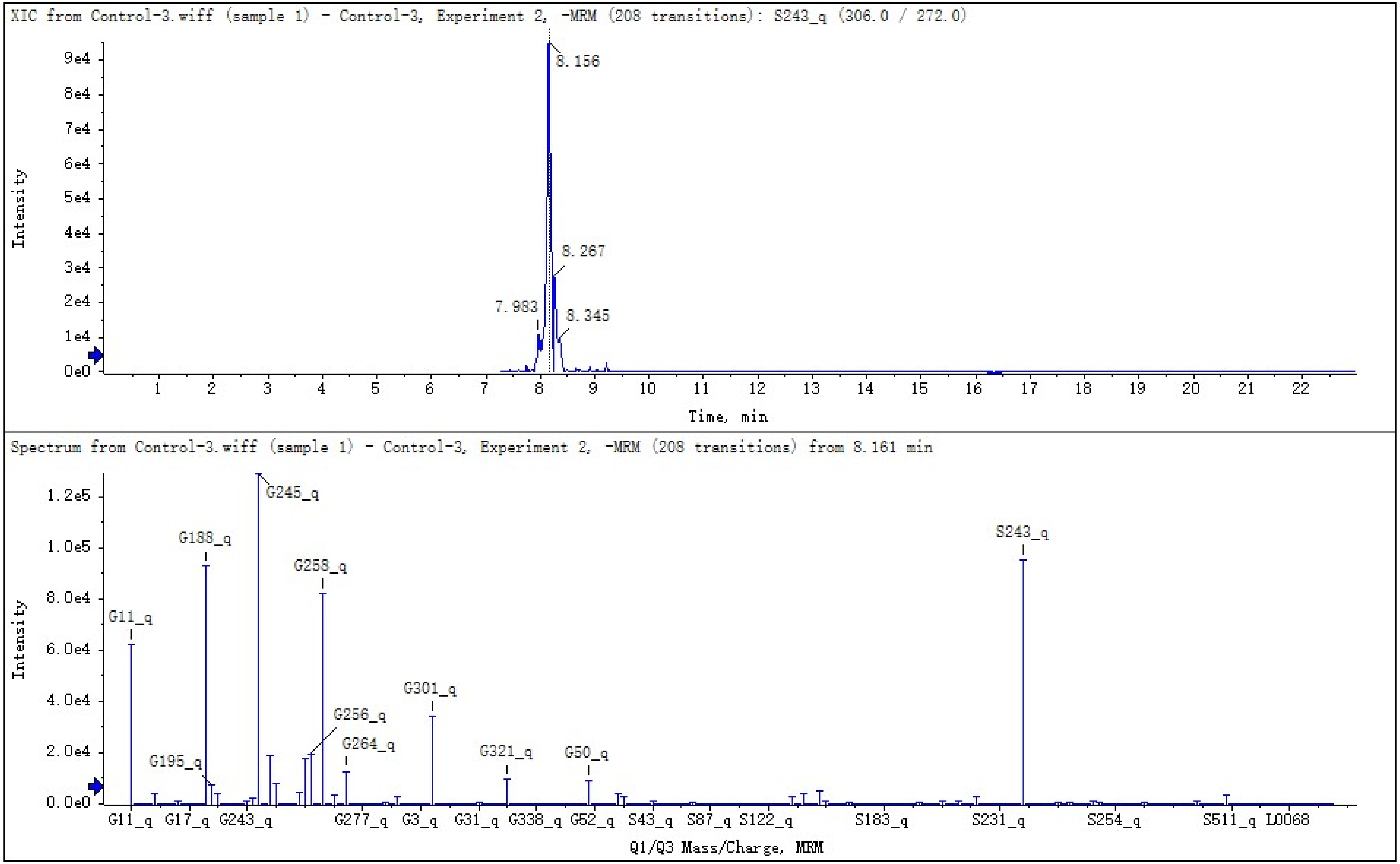

2024/7/18 22:59:14

4.Glutathione, reduced: Targeted metabolomics: Chromatogram and mass spectrogram (Amide column) (control4-0.16%DMSO)

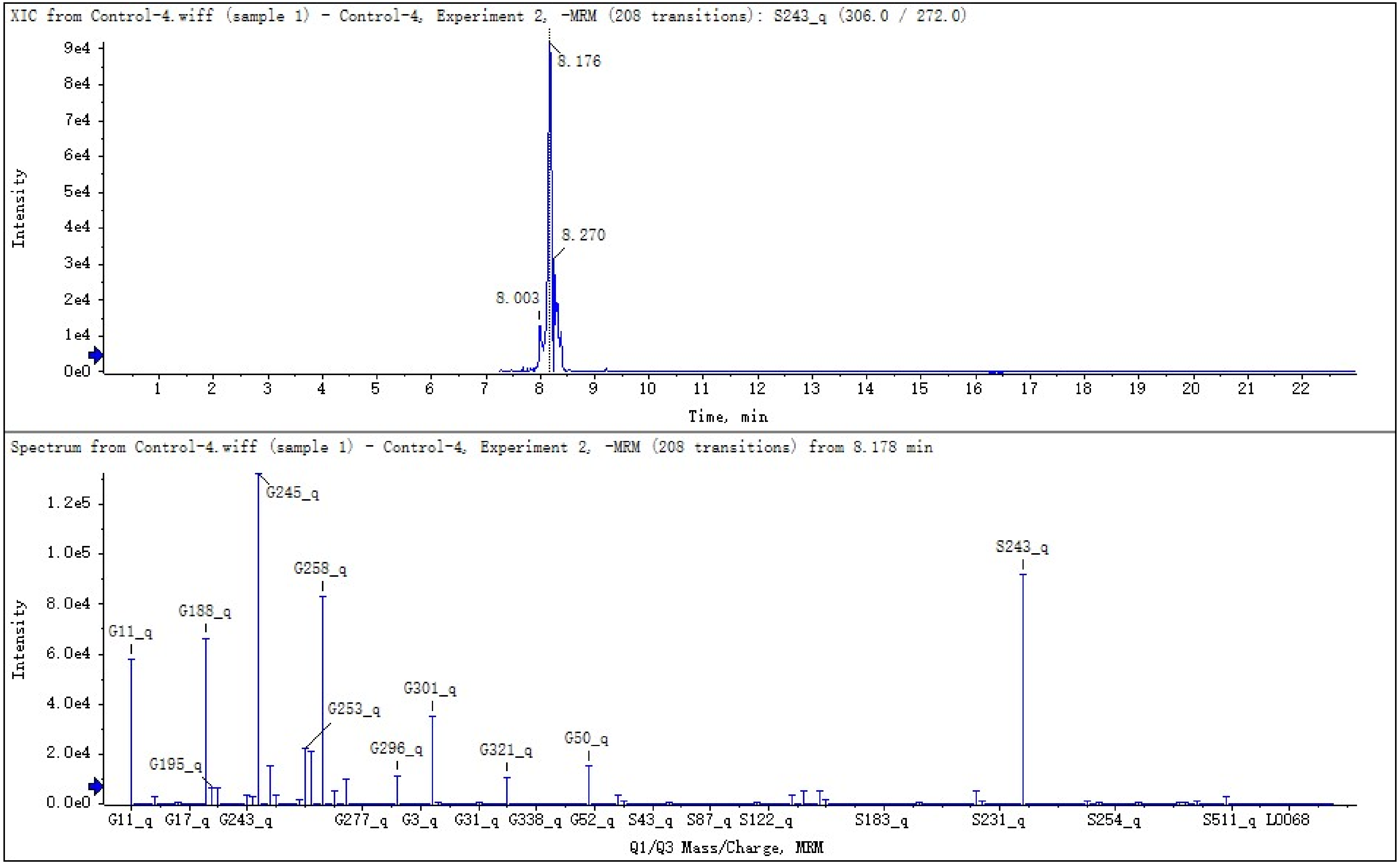

2024/7/18 22:59:56

5.Glutathione, reduced: Targeted metabolomics: Chromatogram and mass spectrogram (Amide column) (control5-0.16%DMSO)

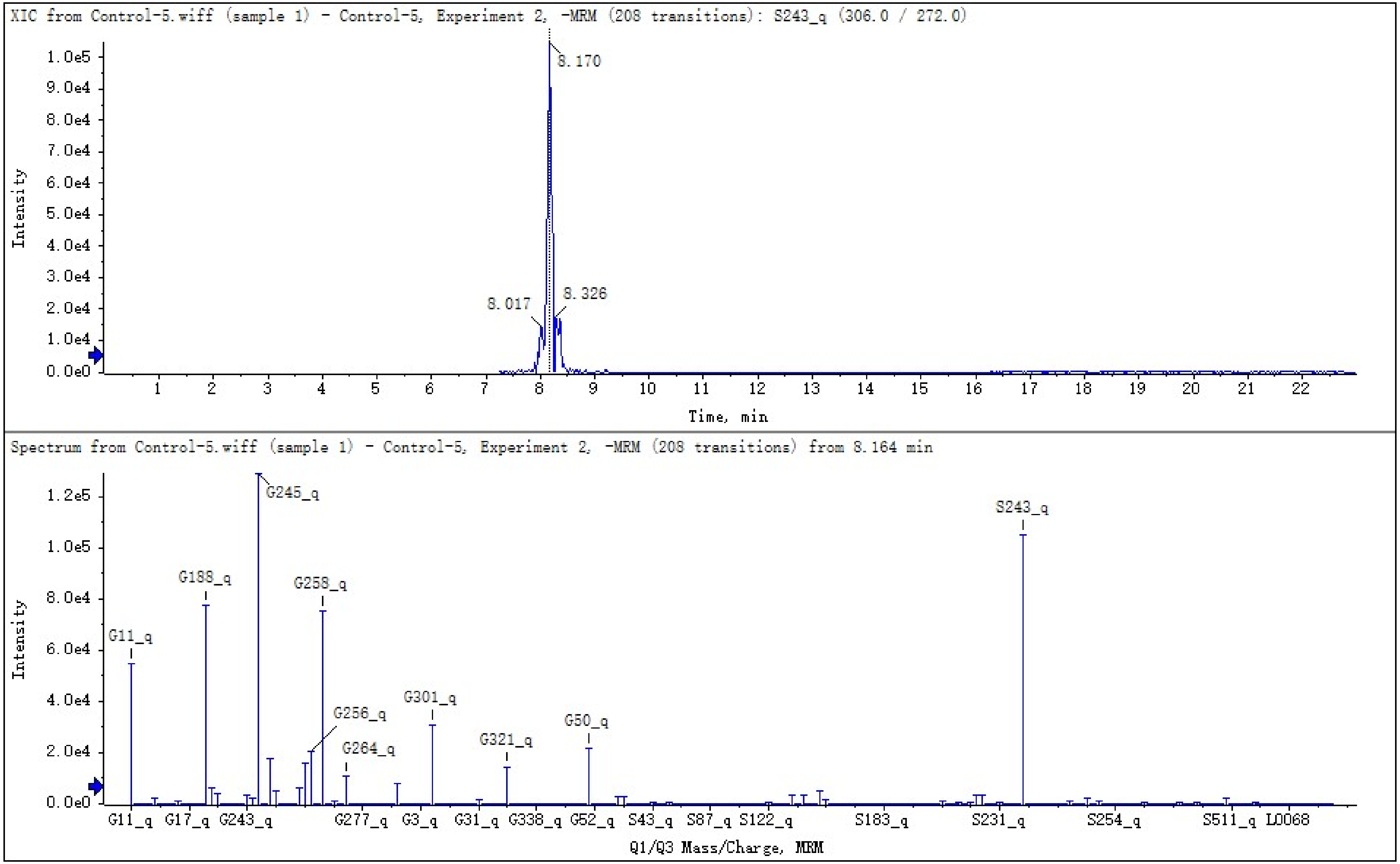

2024/7/18 23:00:43

6.Glutathione, reduced: Targeted metabolomics: Chromatogram and mass spectrogram (Amide column) (control6-0.16%DMSO)

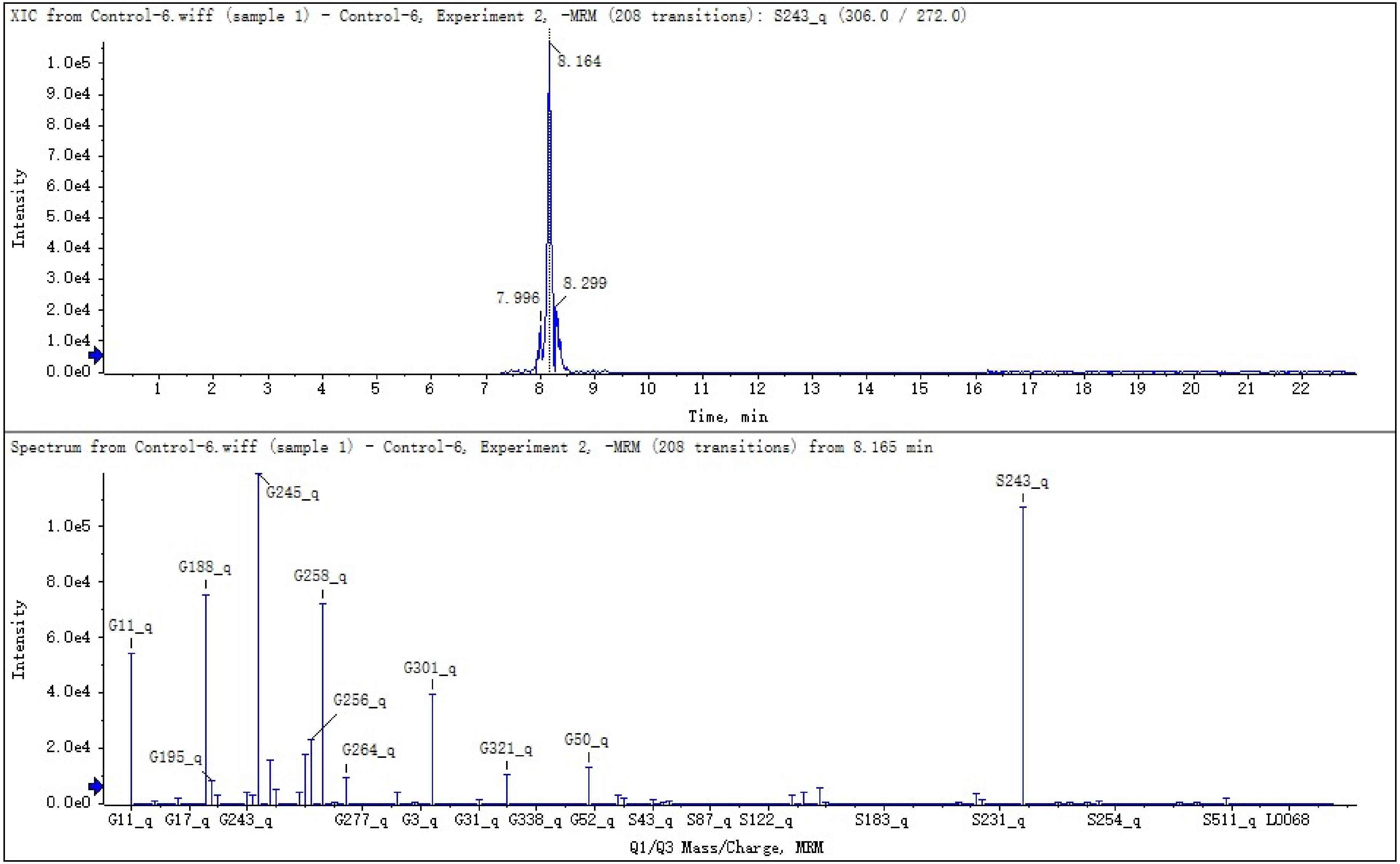

2024/7/18 23:01:40

7.Glutathione, reduced: Targeted metabolomics: Chromatogram and mass spectrogram (Amide column) (treat7-65μM)

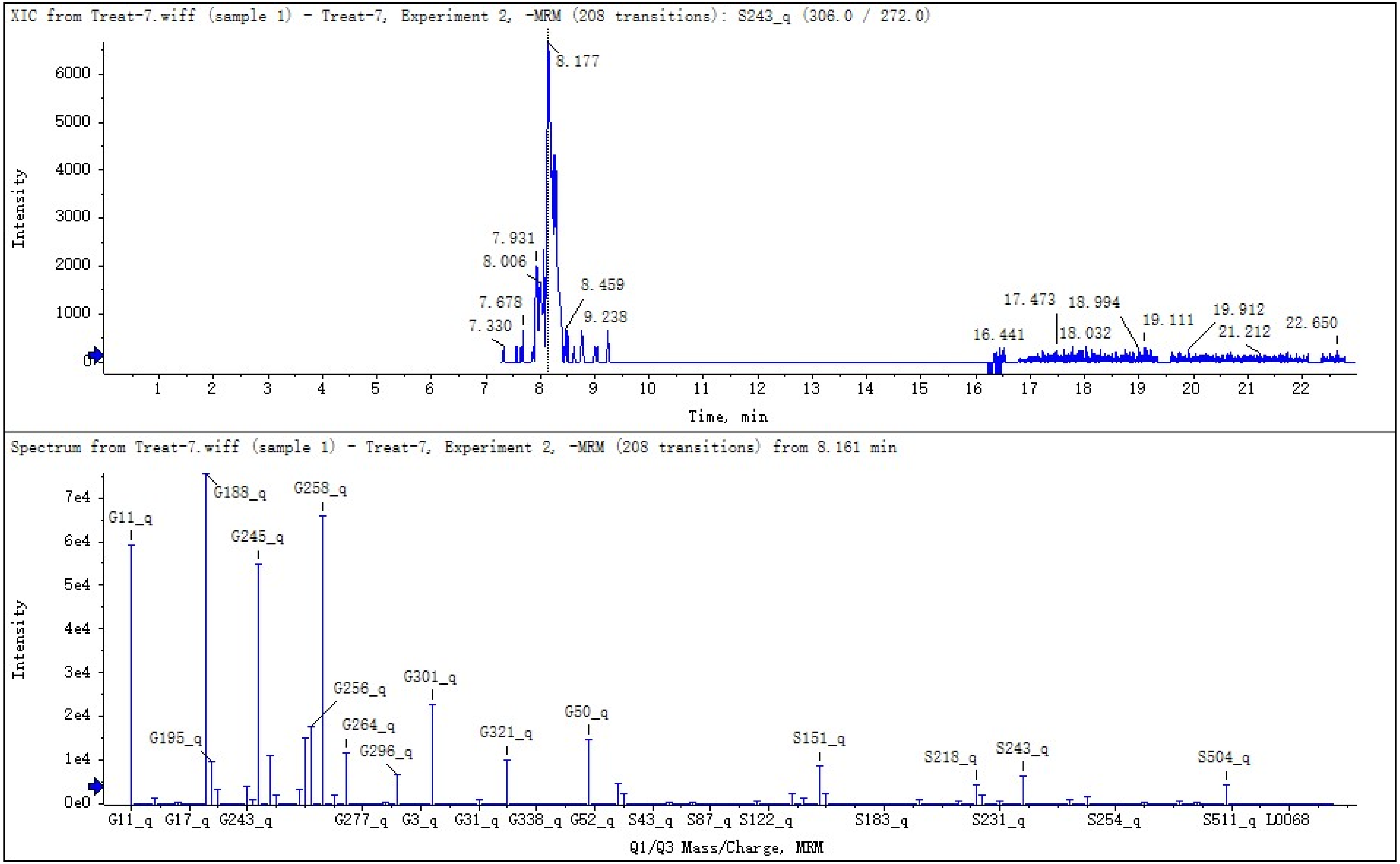

2024/7/18 22:15:53

8.Glutathione, reduced: Targeted metabolomics：Chromatogram and mass spectrogram (Amide column) (treat8-65μM)

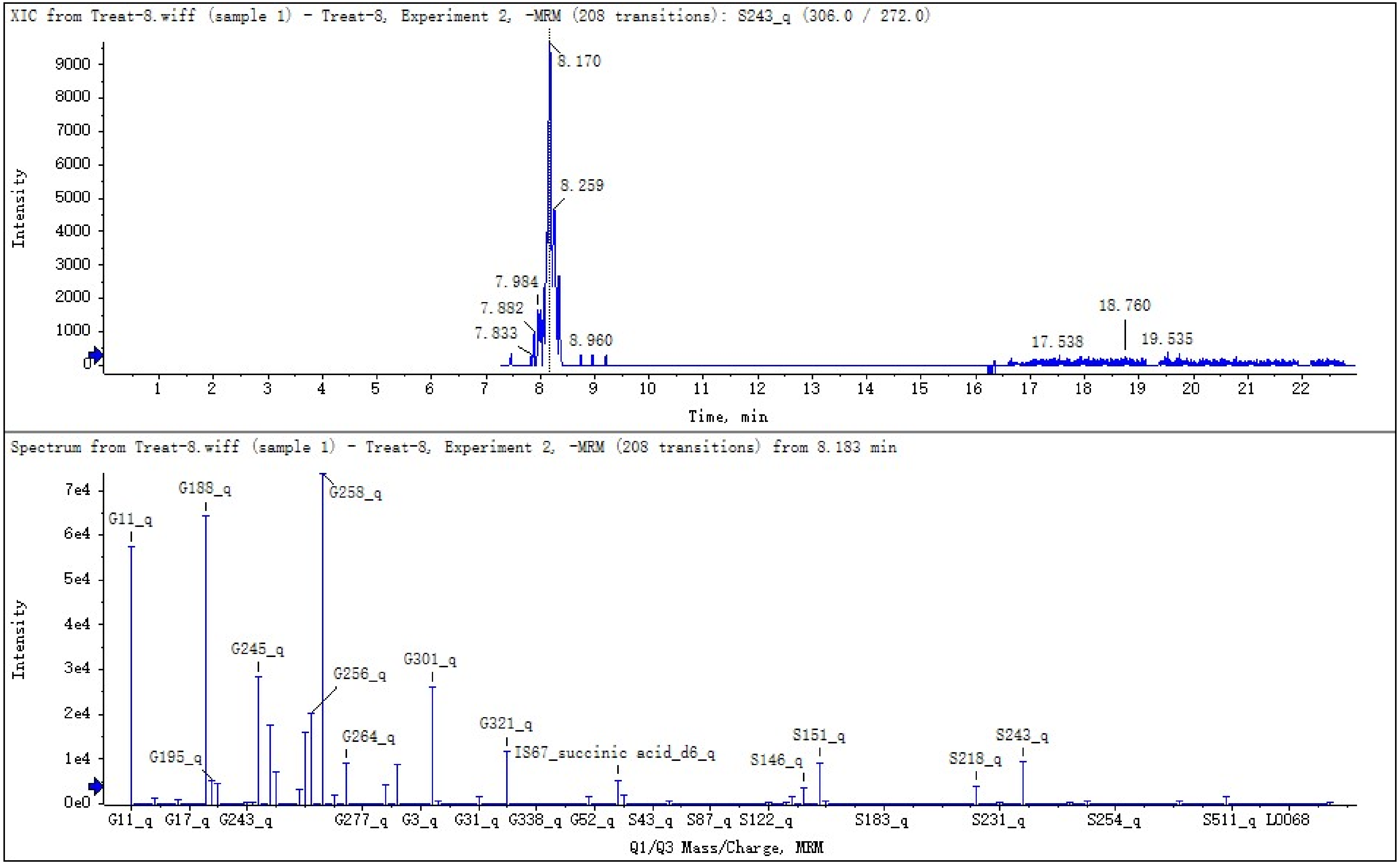

2024/7/18 22:32:10

9.Glutathione, reduced: Targeted metabolomics: Chromatogram and mass spectrogram (Amide column) (treat9-65μM)

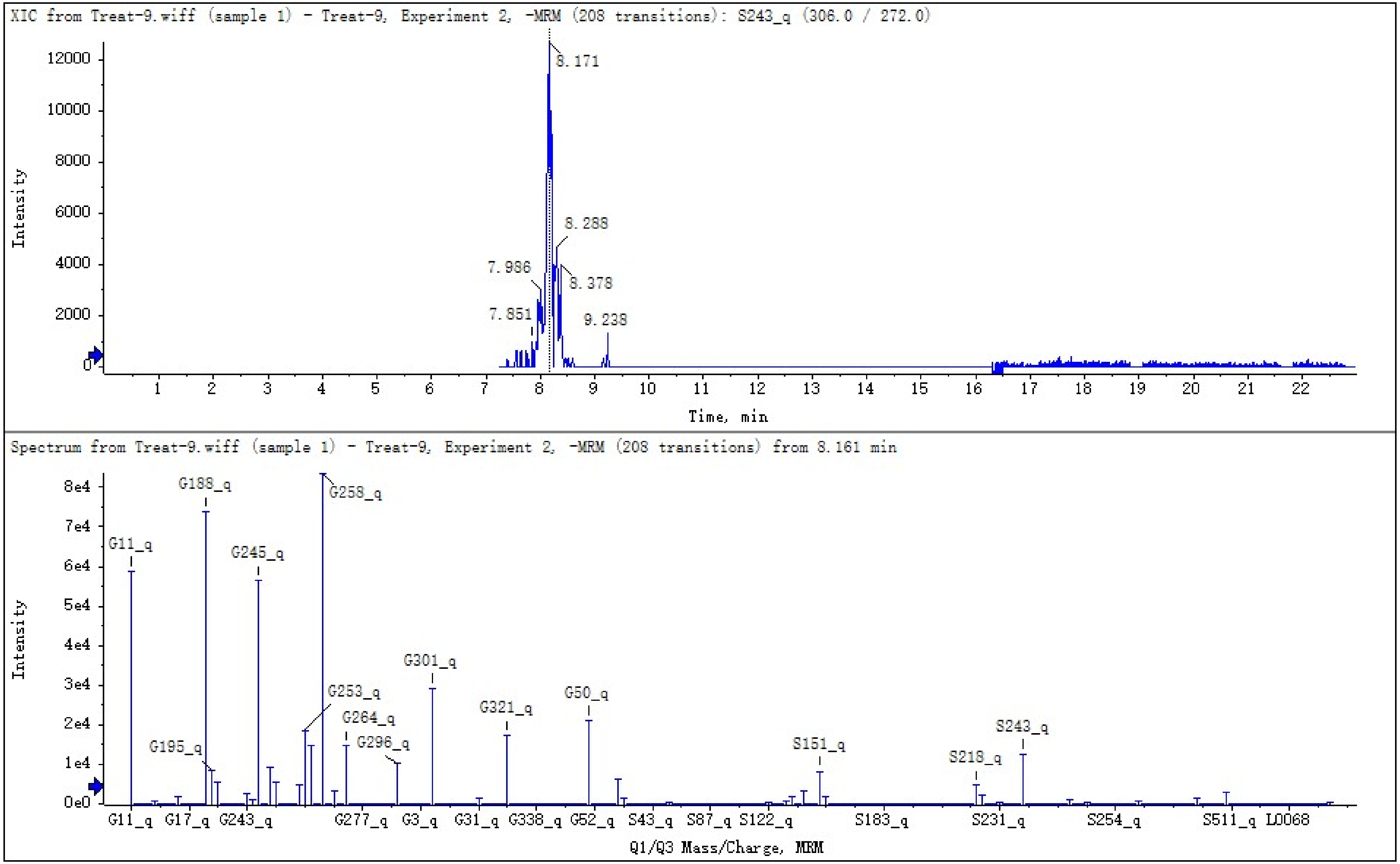

2024/7/18 22:12:54

10.Glutathione, reduced: Targeted metabolomics: Chromatogram and mass spectrogram (Amide column) (treat10-65μM)

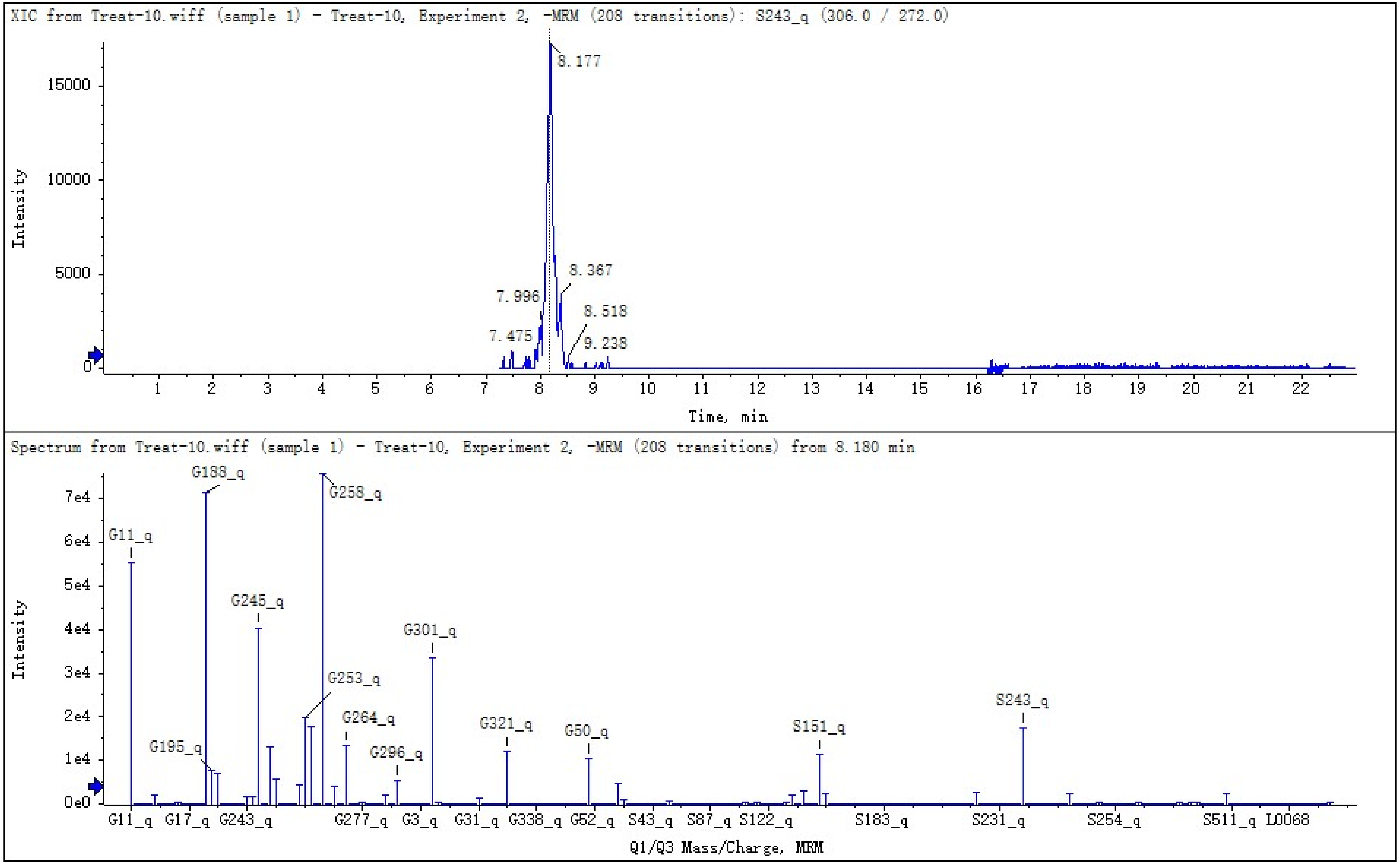

2024/7/18 22:34:45

11.Glutathione, reduced: Targeted metabolomics: Chromatogram and mass spectrogram (Amide column) (treat11-65μM)

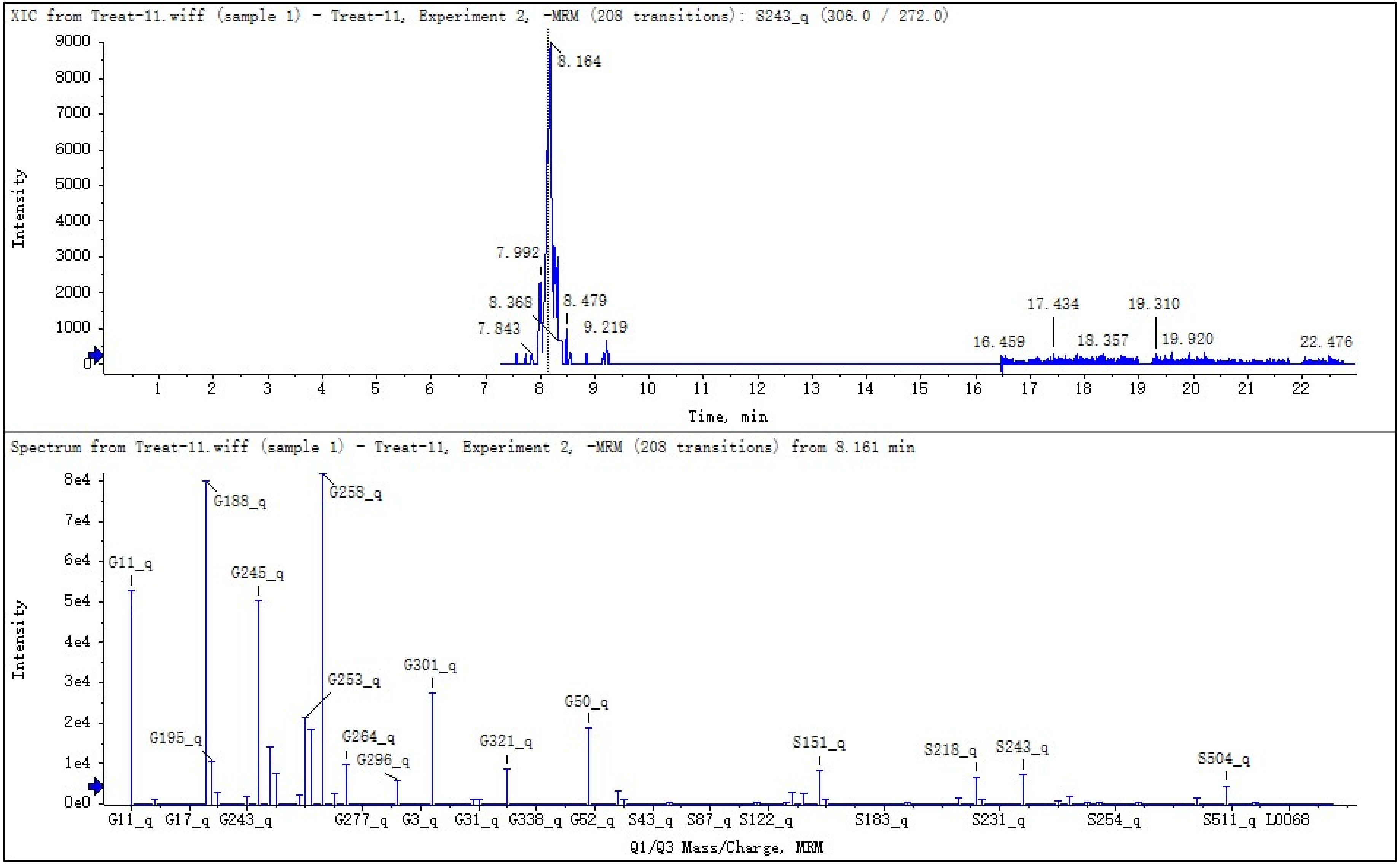

2024/7/18 22:05:13

12.Glutathione, reduced: Targeted metabolomics: Chromatogram and mass spectrogram (Amide column) (treat12-65μM)

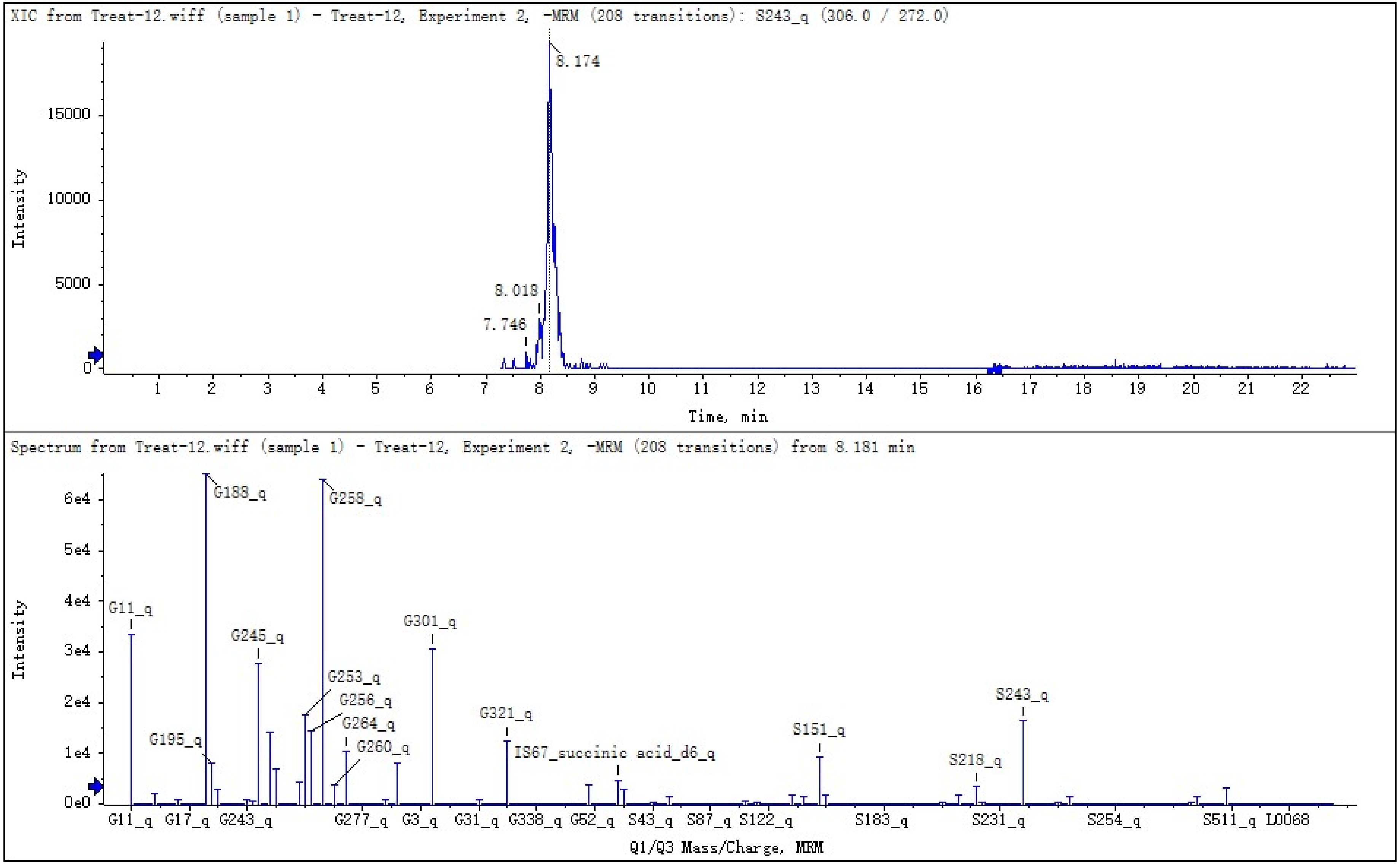

2024/7/18 22:38:39
