# Supplementary material for: 6-Methoxyflavone targets SLC1A5 to induce ferroptosis in HeLa cells
Source: PLoS One. 2025 Dec 29;20(12):e0339578. doi: 10.1371/journal.pone.0339578 (PMC12747331; doi:10.1371/journal.pone.0339578)
Supplement: S4 File — Page 1. The extracted ion chromatogram and mass spectrogram of glutamate in targeted metabolomics (amide column) in the control group (0.16% DMSO). Page 2. The extracted ion chromatogram and mass spectrogram of glutamate in targeted metabolomics (amide column) in the treat group (65 μM). Page 3. The extracted ion chromatogram and mass spectrogram of arachidonic acid in targeted metabolomics (C18 column) in the control group (0.16% DMSO). Page 4. The extracted ion chromatogram and mass spectrogram of arachidonic acid in targeted metabolomics (C18 column) in the treat group (65 μM). Page 5. The extracted ion chromatogram and mass spectrogram of adrenic acid in targeted metabolomics (C18 column) in the control group (0.16% DMSO). Page 6. The extracted ion chromatogram and mass spectrogram of adrenic acid in targeted metabolomics (C18 column) in the treat group (65 μM). Page 7. The extracted ion chromatogram and mass spectrogram of cystine in targeted metabolomics (amide column) in the control group (0.16% DMSO). Page 8. The extracted ion chromatogram and mass spectrogram of cystine in targeted metabolomics (amide column) in the treat group (65 μM). Page 9. The extracted ion chromatogram and mass spectrogram of glutamine in targeted metabolomics (amide column) in the control group (0.16% DMSO). Page 10. The extracted ion chromatogram and mass spectrogram of glutamine in targeted metabolomics (amide column) in the treat group (65 μM). (PDF) [file pone.0339578.s004.pdf]

1.Glutamate: Targeted metabolomics: Extracted ion chromatogram and mass spectrogram (control group-0.16%DMSO) (Amide column)

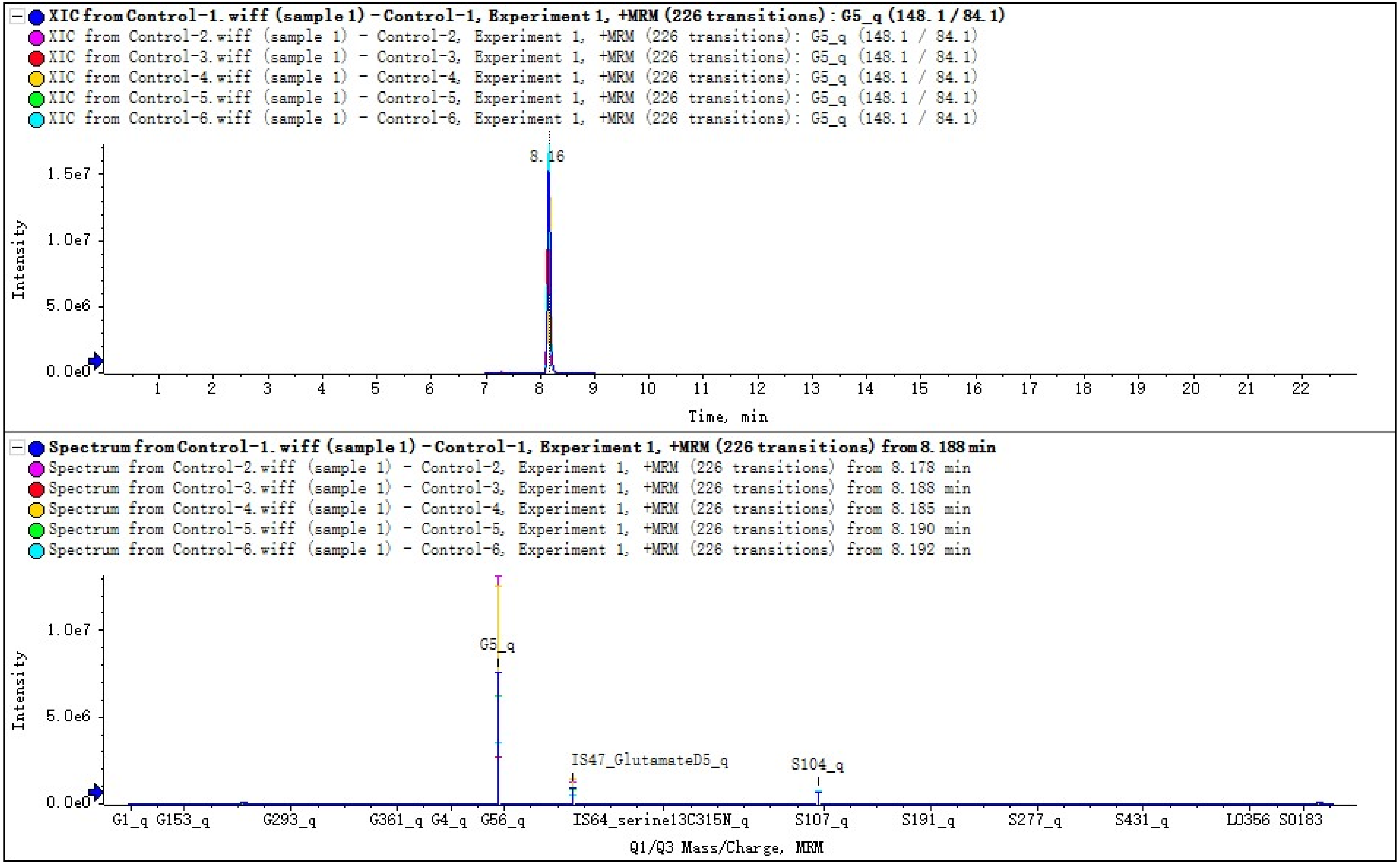

2024/7/18 23:47:17

2.Glutamate: Targeted metabolomics：Extracted ion chromatogram and mass spectrogram (treat group-65μM) (Amide column)

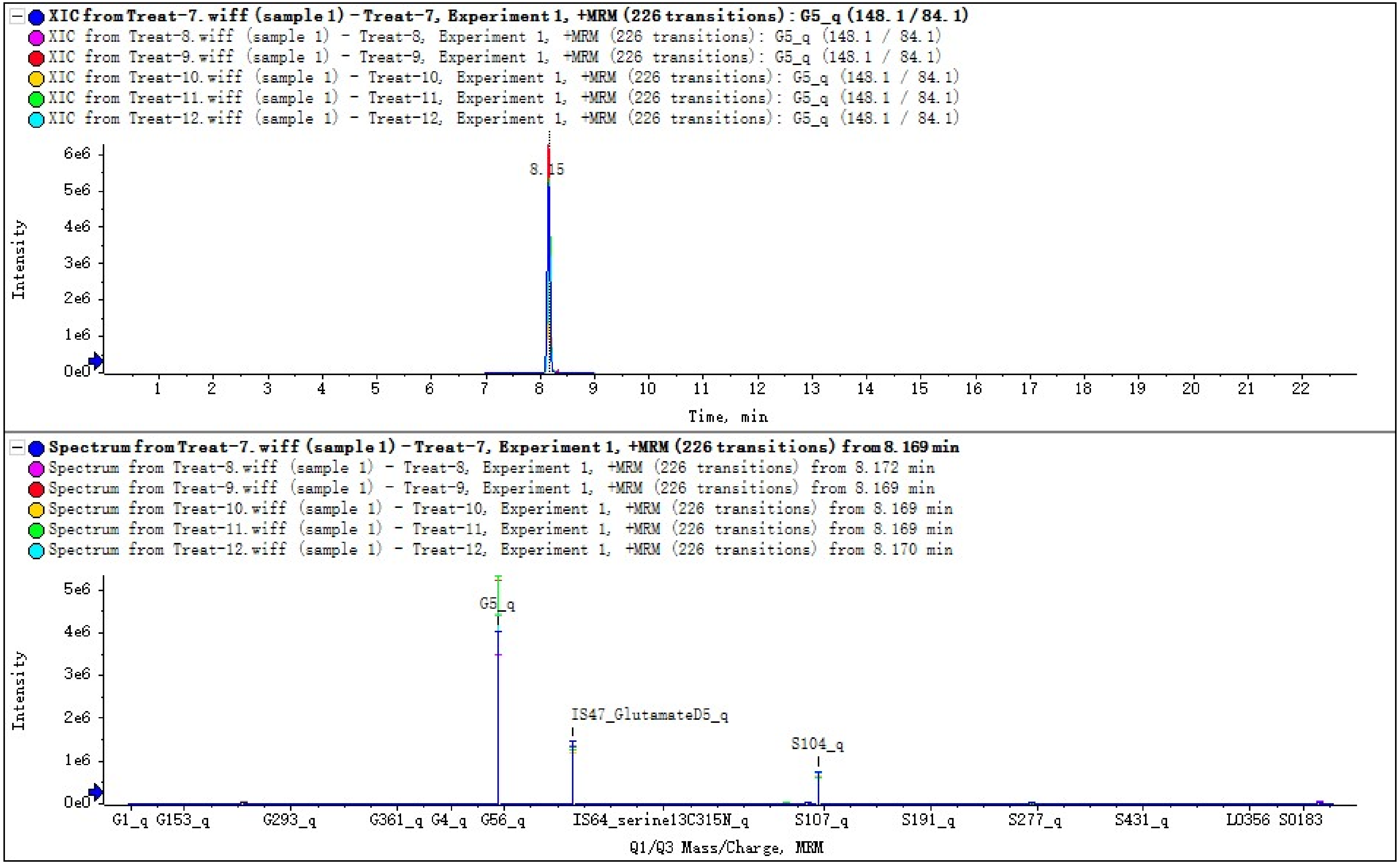

2024/7/18 23:49:32

3.Arachidonic acid: Targeted metabolomics: Extracted ion chromatogram and mass spectrogram (control group-0.16%DMSO) (C18 column)

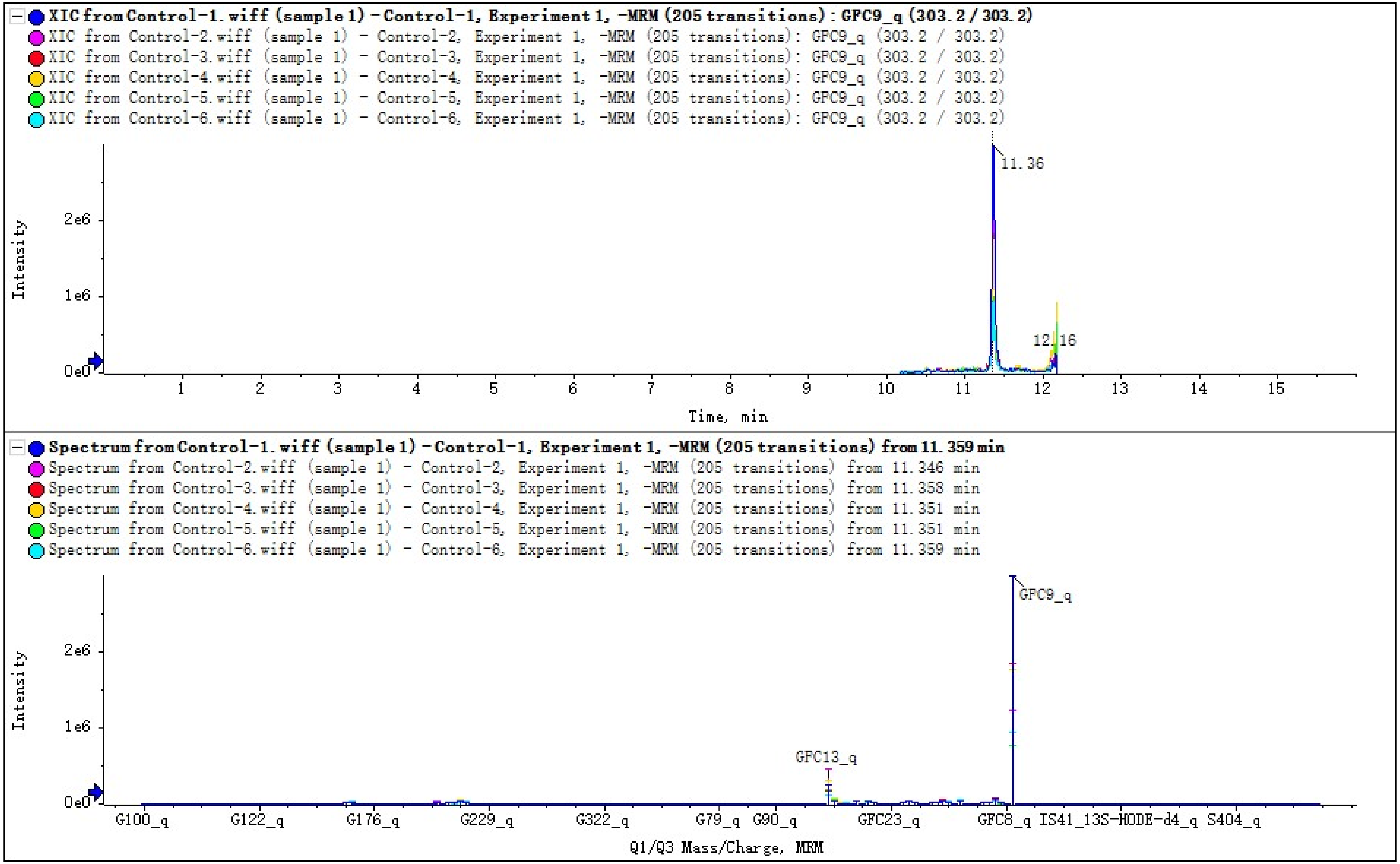

2024/7/24 15:22:26

4.Arachidonic acid: Targeted metabolomics: Extracted ion chromatogram and mass spectrogram (treat group-65μM) (C18 column)

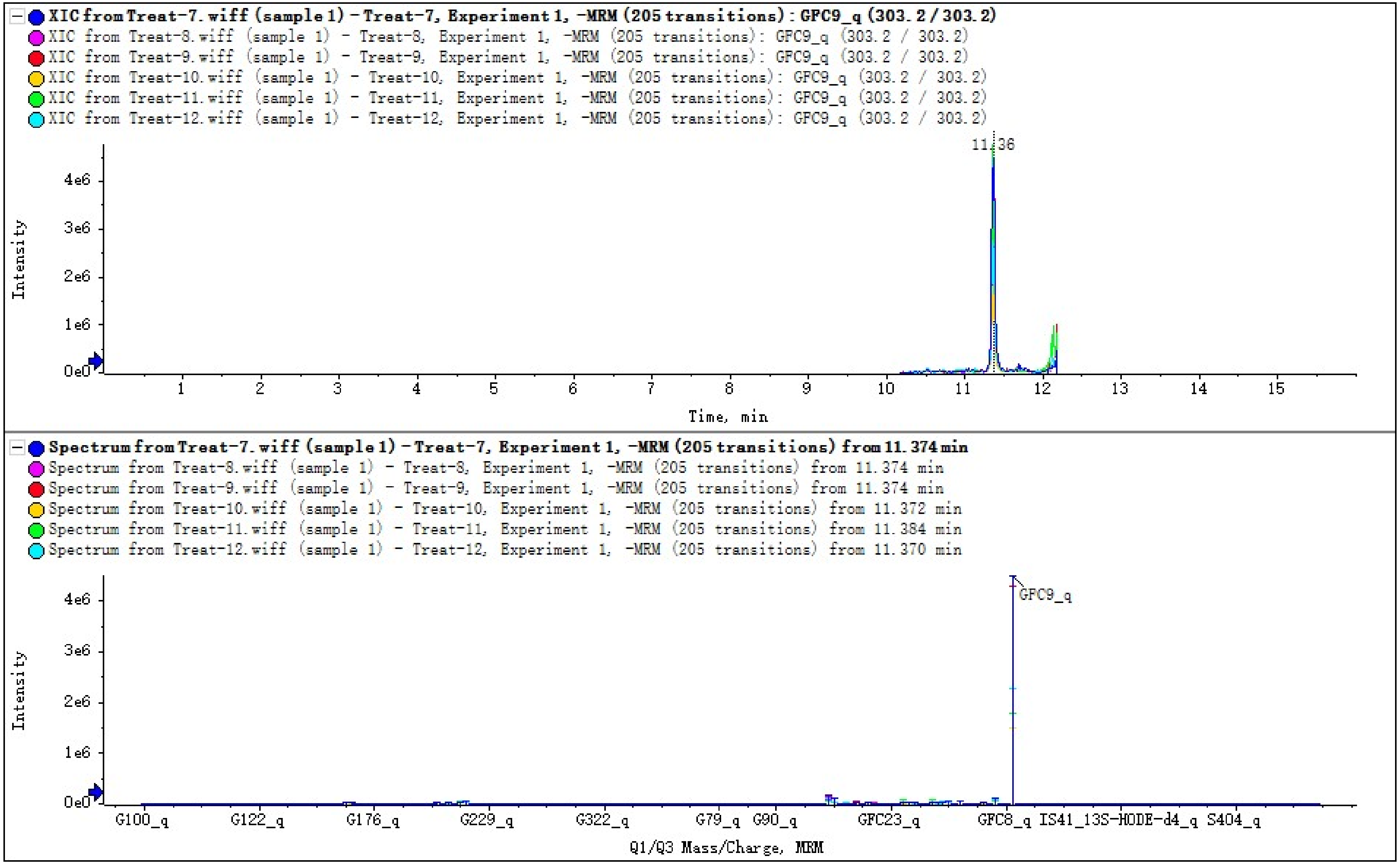

2024/7/24 15:26:31

5.Adrenic acid: Targeted metabolomics: Extracted ion chromatogram and mass spectrogram (control group-0.16%DMSO) (C18 column)

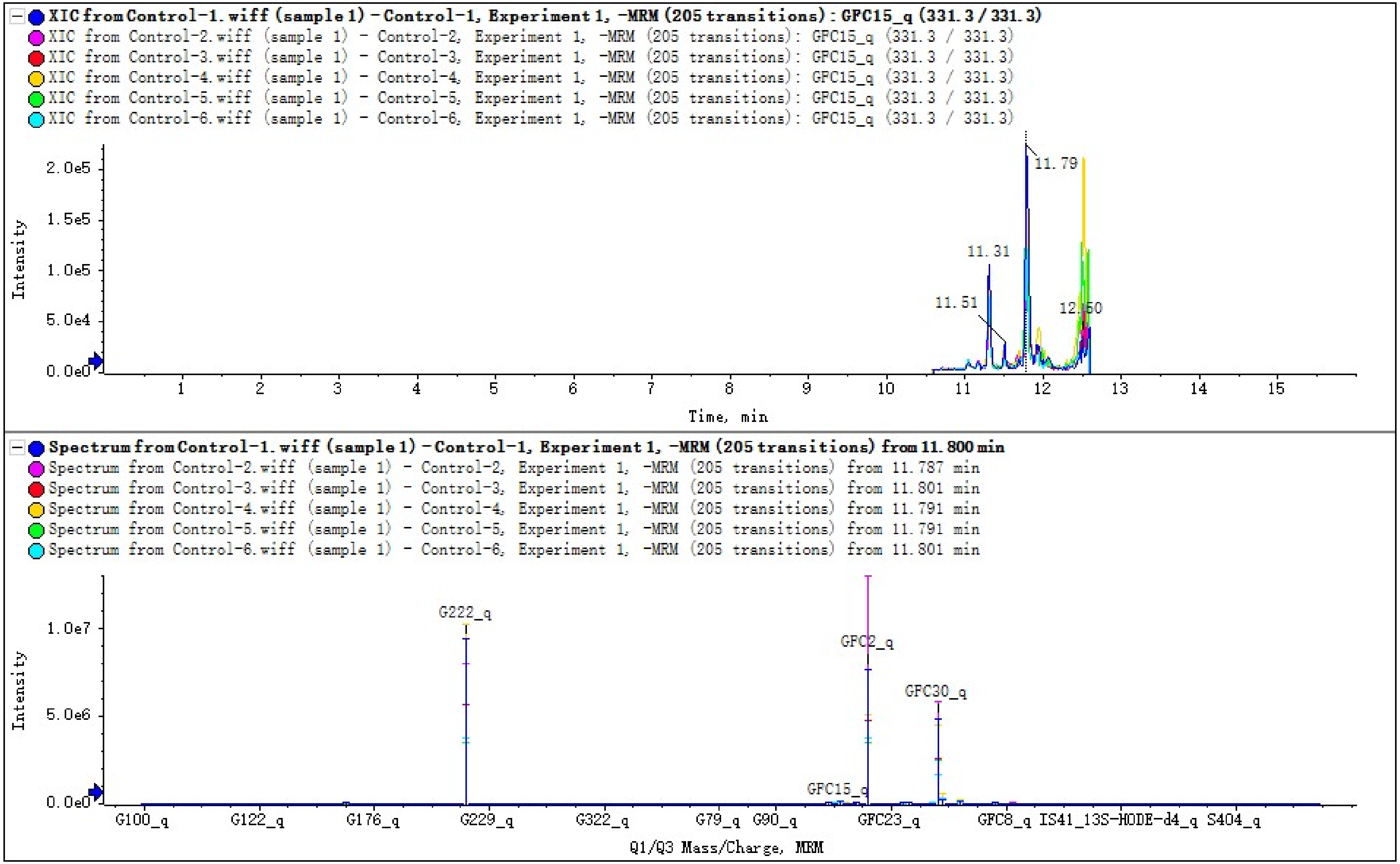

2024/7/19 0:11:33

6.Adrenic acid: Targeted metabolomics: Extracted ion chromatogram and mass spectrogram (treat group-65μM) (C18 column)

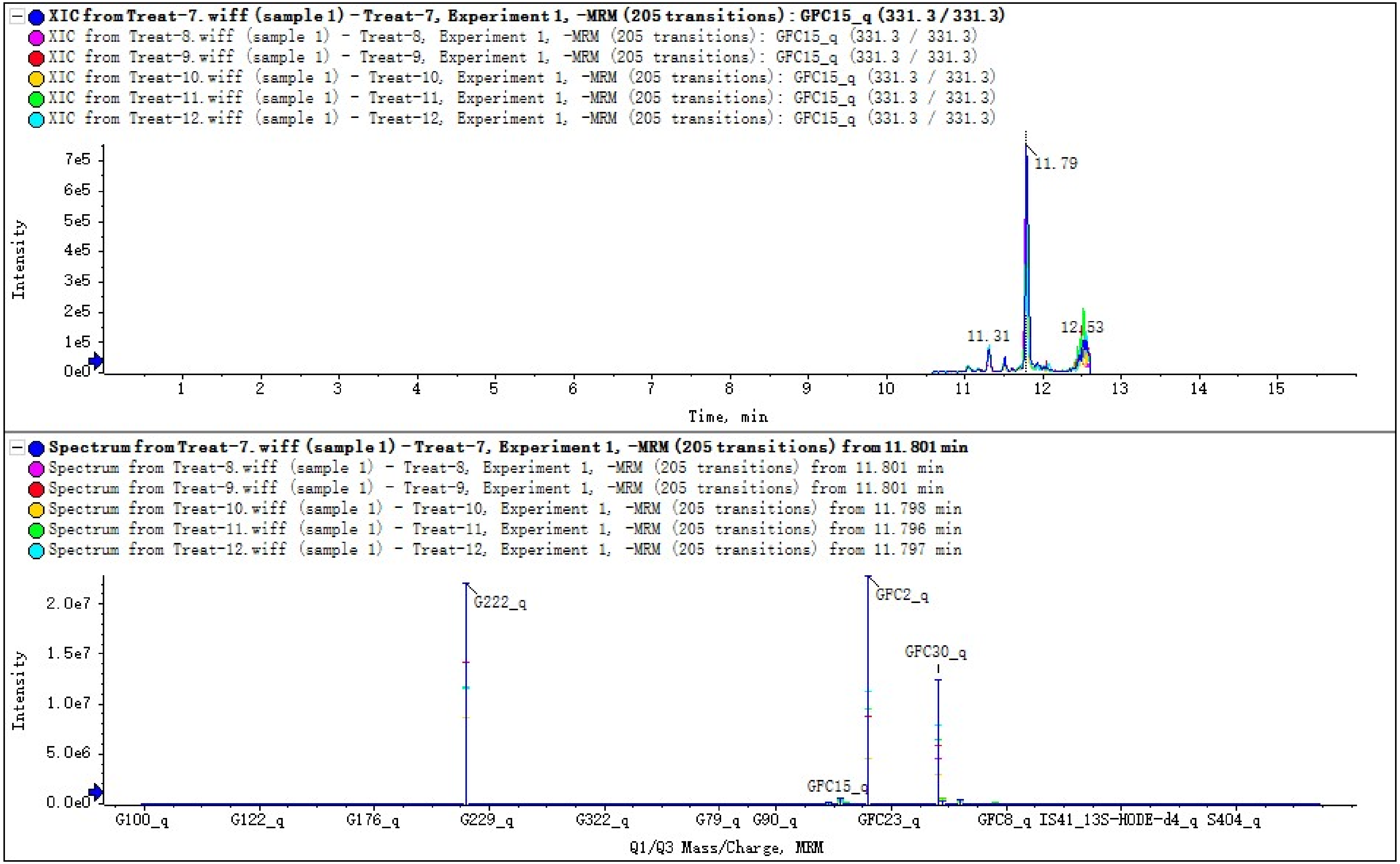

2024/7/19 0:14:54

7.Cystine: Targeted metabolomics：Extracted ion chromatogram and mass spectrogram (control group-0.16%DMSO) (Amide column)

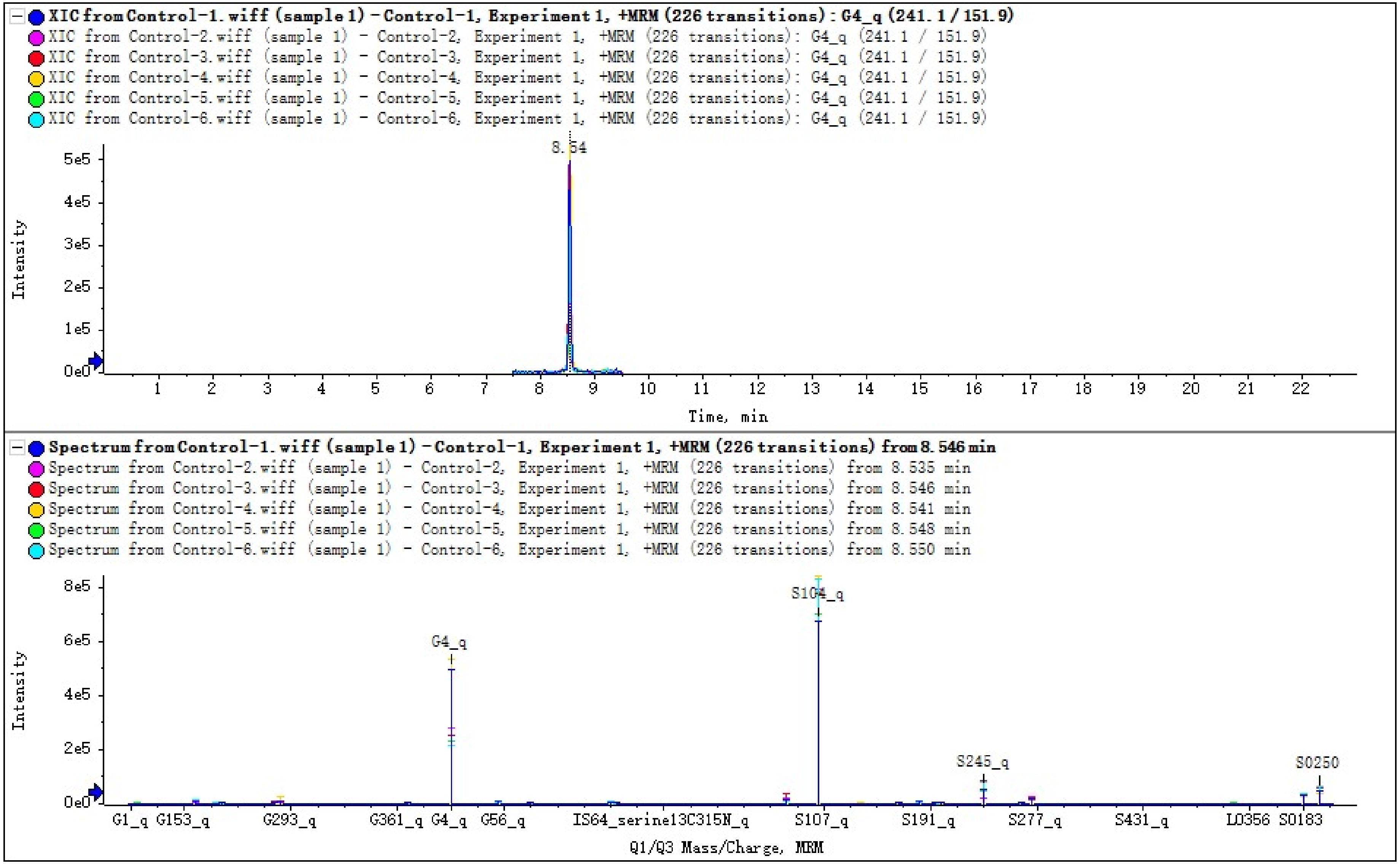

2024/7/19 0:20:43

8.Cystine: Targeted metabolomics：Extracted ion chromatogram and mass spectrogram (treat group-65μM) (Amide column)

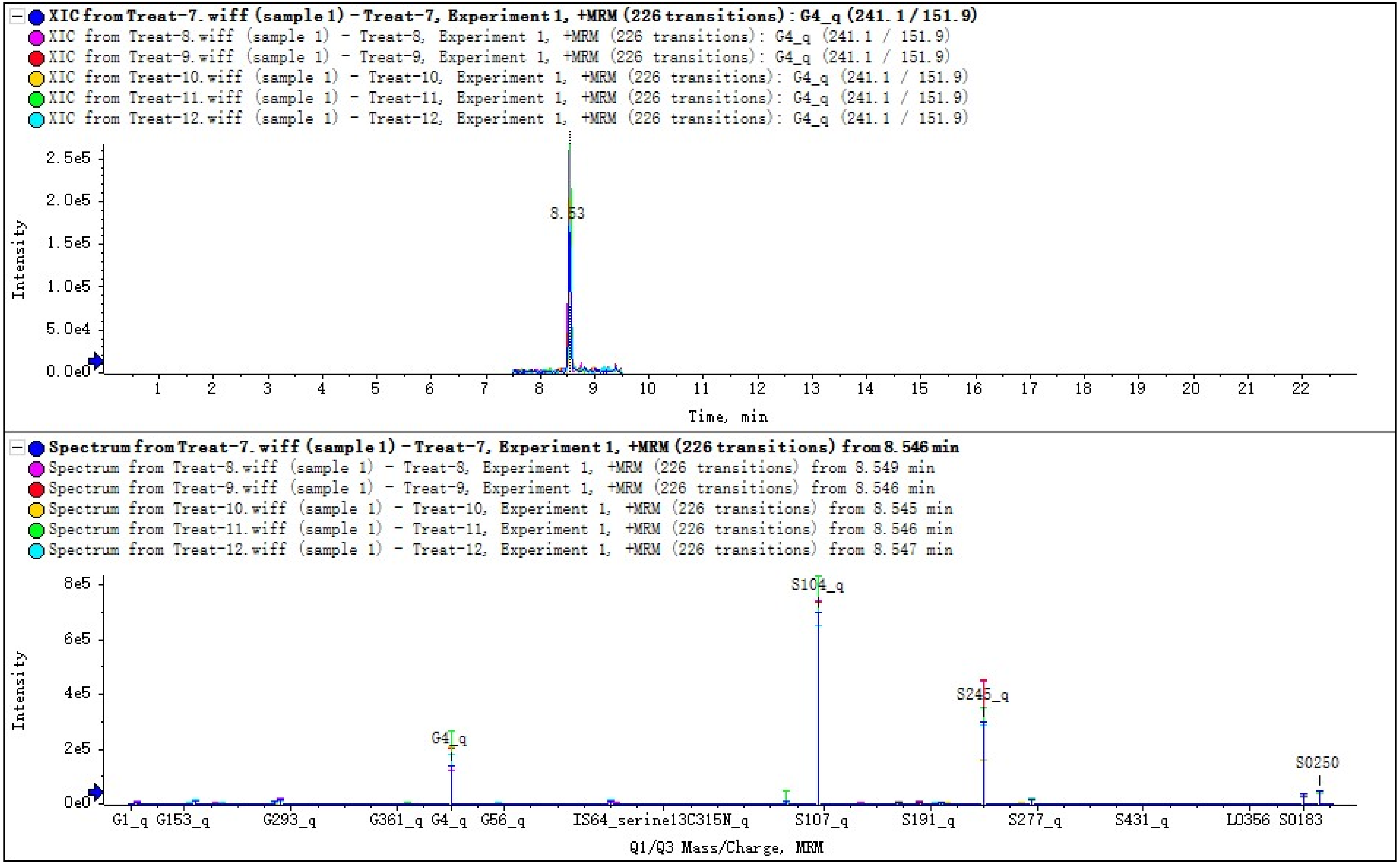

2024/7/19 0:22:20

9.Glutamine: Targeted metabolomics: Extracted ion chromatogram and mass spectrogram (control group-0.16%DMSO) (Amide column)

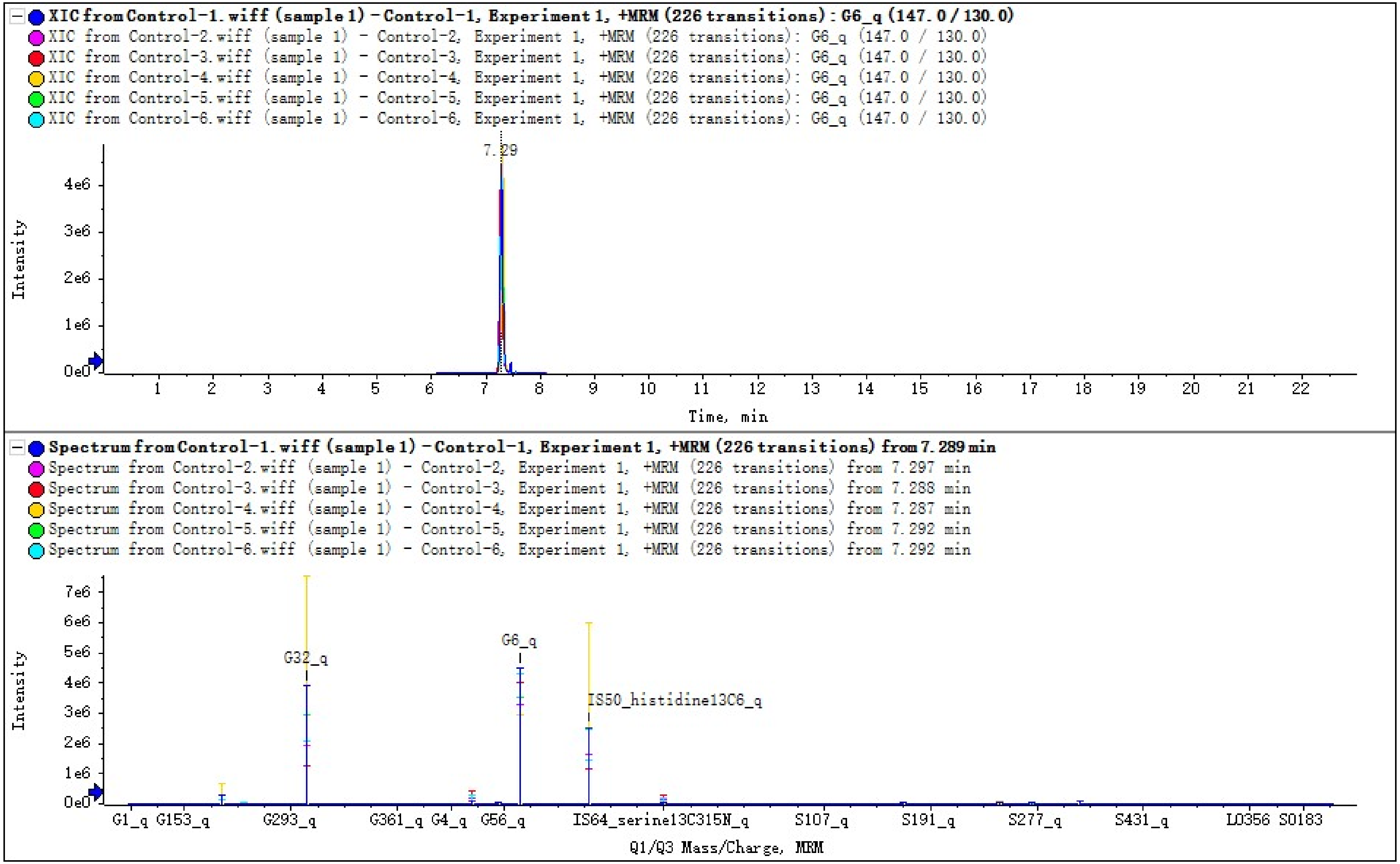

2024/7/19 8:18:11

10.Glutamine: Targeted metabolomics: Extracted ion chromatogram and mass spectrogram (treat group-65μM) (Amide column)

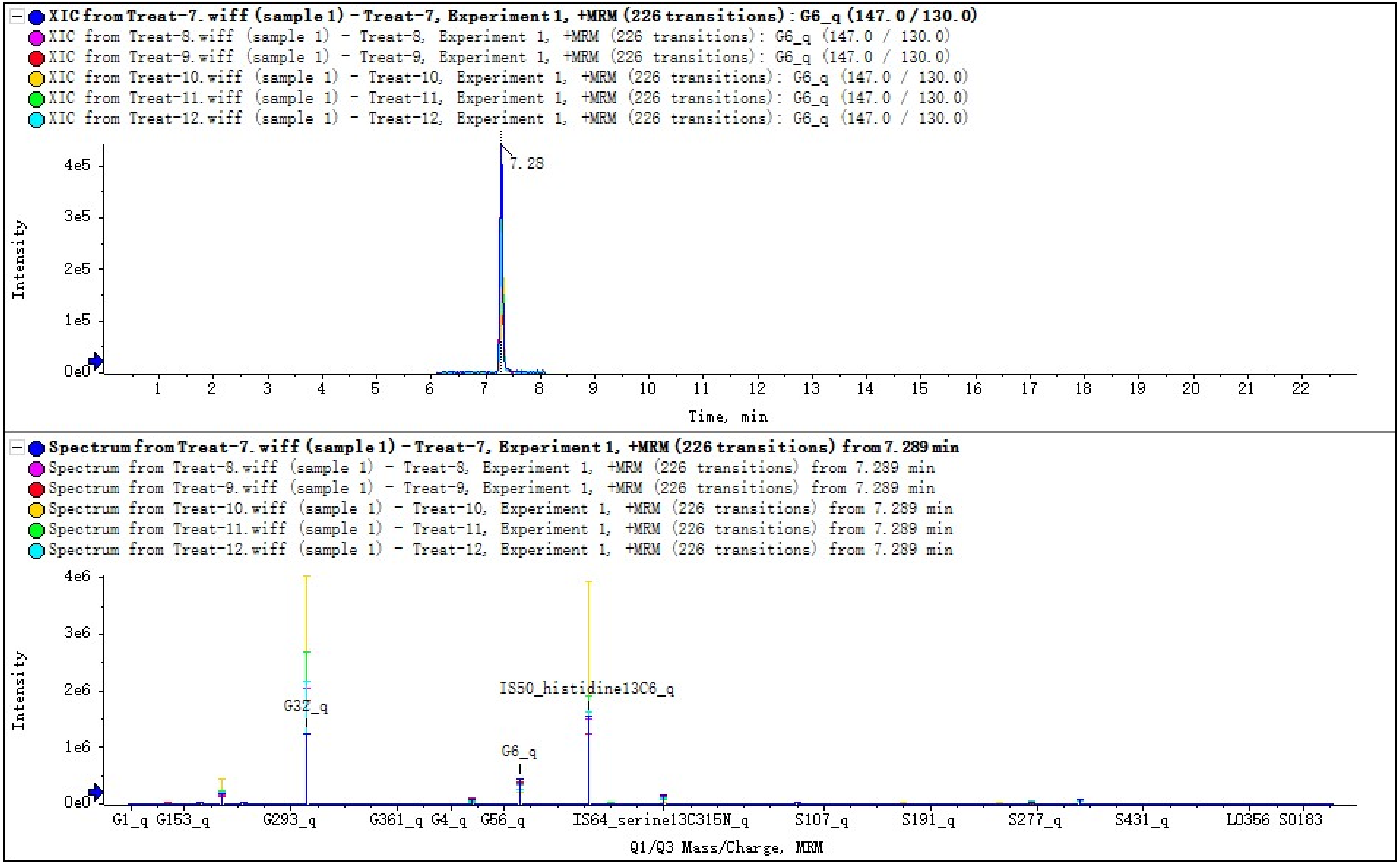

2024/7/19 8:19:44
