# Supplementary material for: 6-Methoxyflavone targets SLC1A5 to induce ferroptosis in HeLa cells
Source: PLoS One. 2025 Dec 29;20(12):e0339578. doi: 10.1371/journal.pone.0339578 (PMC12747331; doi:10.1371/journal.pone.0339578)

D:\1\CONTROL1 08/14/23 04:54:27

1. ASNS(VDGEIILHLYDK):Chromatogram and mass spectrogram of PRM quantitative detection:control group(0.16%DMSO)

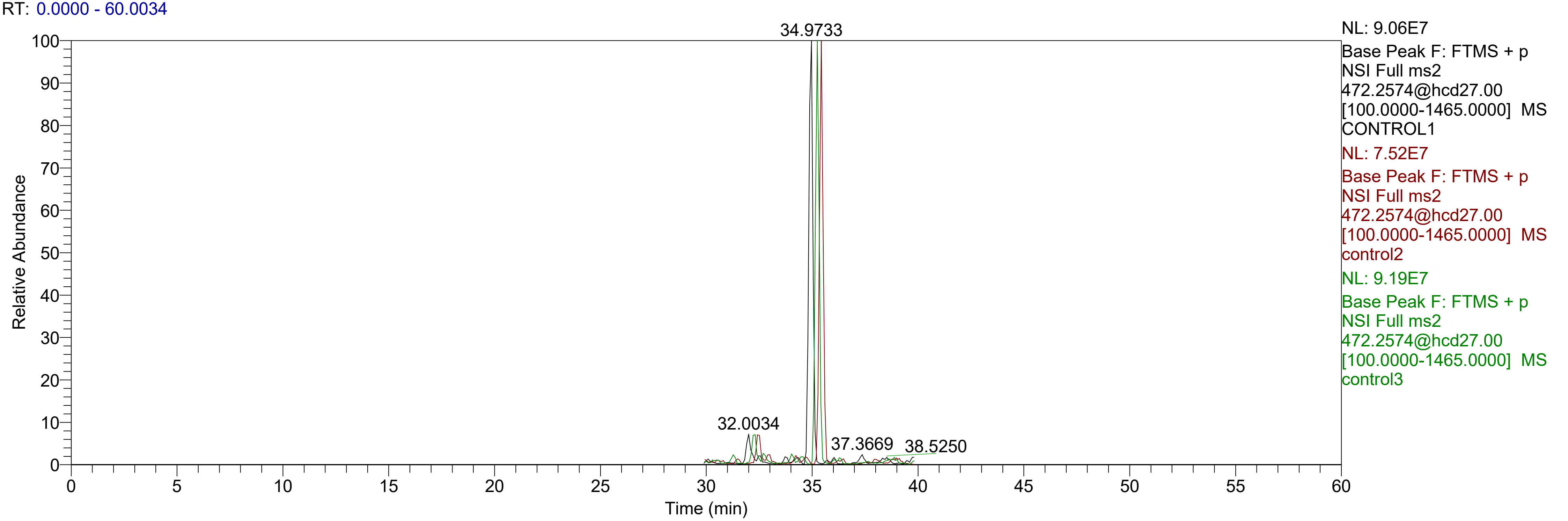


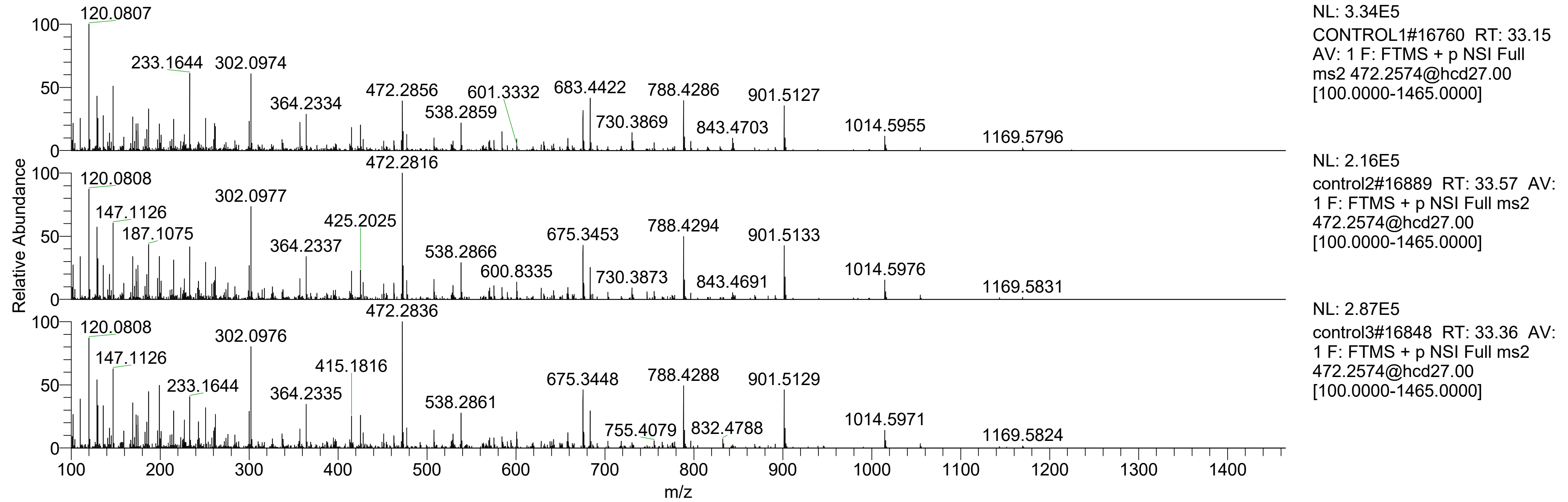

D:\1\TREAT4 08/14/23 09:36:13

2.ASNS(VDGEIILHLYDK):Chromatogram and mass spectrogram of PRM quantitative detection:treat group(65μM)

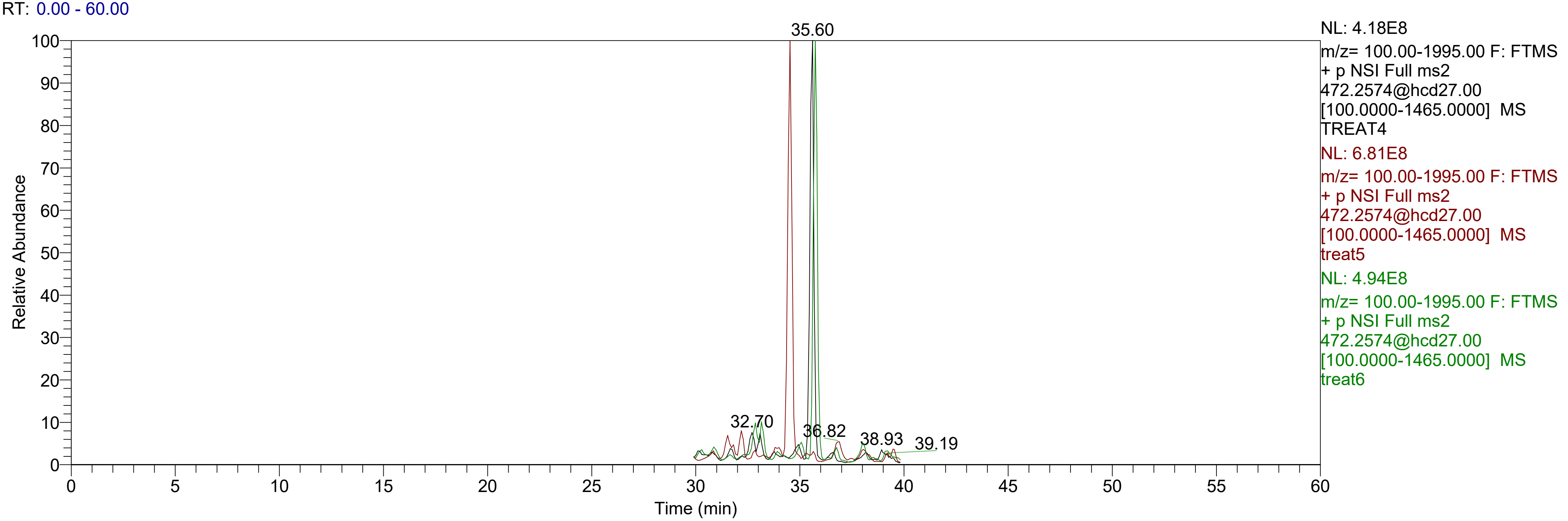


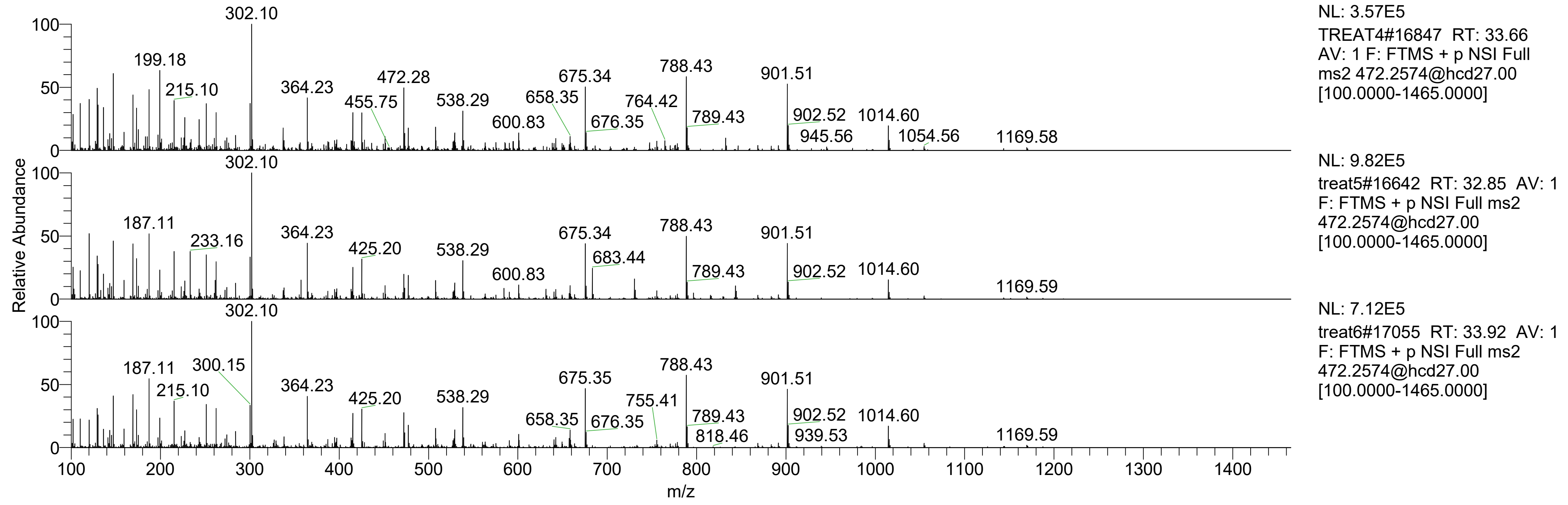

D:\1\CONTROL1 08/14/23 04:54:27

3.SLC1A5(EVLDSFLDLAR):Chromatogram and mass spectrogram of PRM quantitative detection:control group(0.16%DMSO)

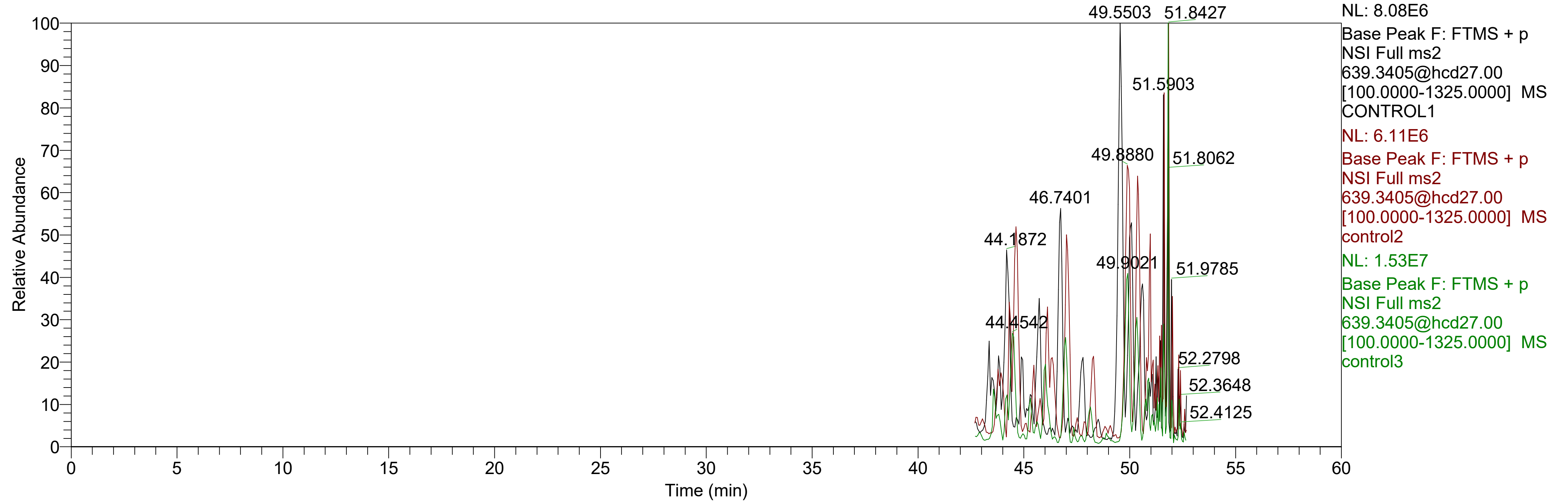


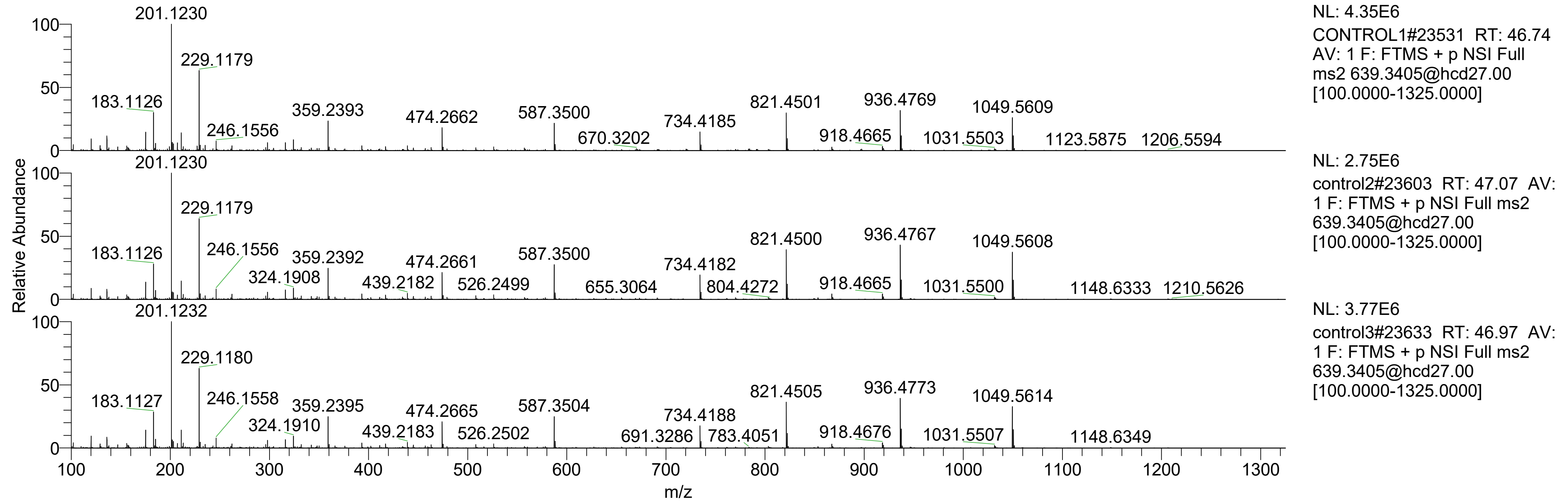

D:\1\TREAT4 08/14/23 09:36:13

4.SLC1A5(EVLDSFLDLAR):Chromatogram and mass spectrogram of PRM quantitative detection:treat group(65μM)

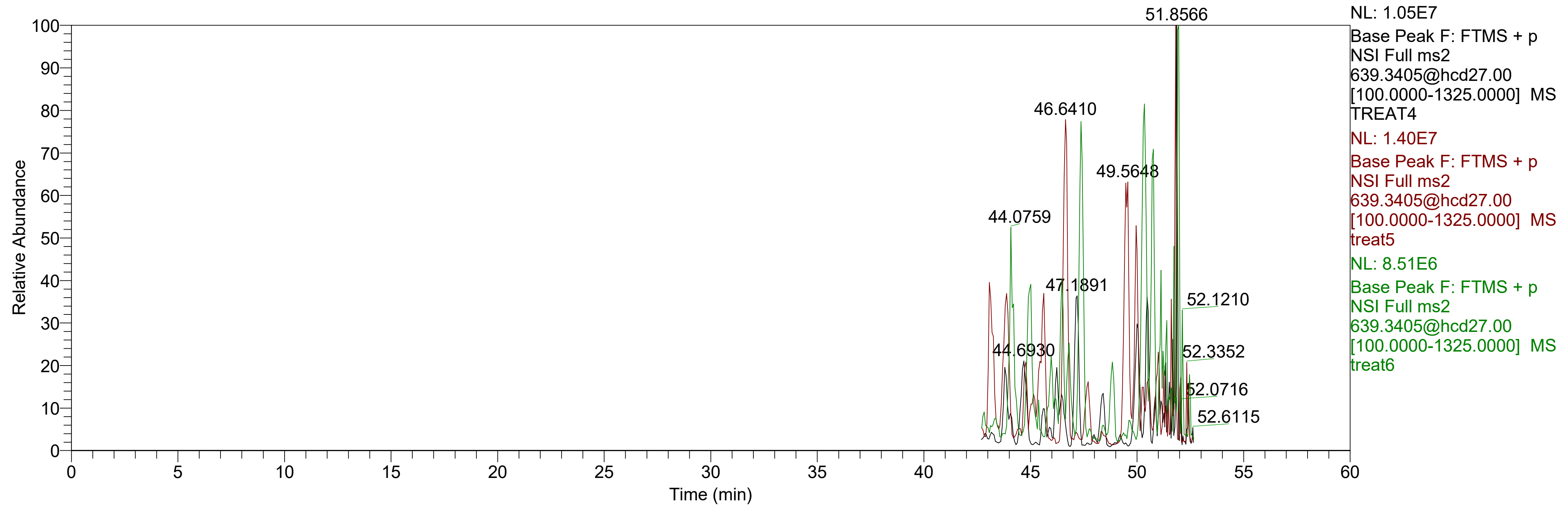


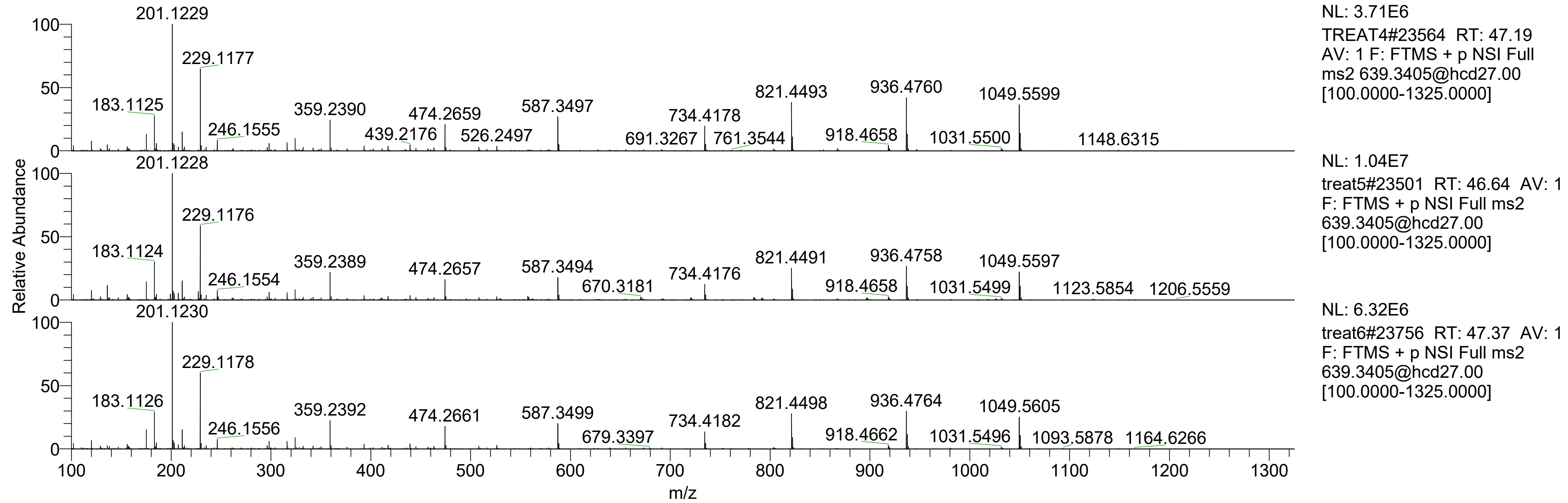

Supplement: S5 File — Page 1. The chromatogram and mass spectrogram of ASNS(VDGEIILHLYDK) in PRM quantitative detection in the control group (0.16% DMSO). Page 2. The chromatogram and mass spectrogram of ASNS(VDGEIILHLYDK) in PRM quantitative detection in the treat group (65 μM). Page 3. The chromatogram and mass spectrogram of SLC1A5(EVLDSFLDLAR) in PRM quantitative detection in the control group (0.16% DMSO). Page 4. The chromatogram and mass spectrogram of SLC1A5(EVLDSFLDLAR) in PRM quantitative detection in the treat group (65 μM). (PDF) [file pone.0339578.s005.pdf]
