# Supplementary material for: 6-Methoxyflavone targets SLC1A5 to induce ferroptosis in HeLa cells
Source: PLoS One. 2025 Dec 29;20(12):e0339578. doi: 10.1371/journal.pone.0339578 (PMC12747331; doi:10.1371/journal.pone.0339578)
Supplement: S7 File — Page 1. New transcript sequence file of SLC1A5. Page 2. New transcript sequence file of ASNS. (PDF) [file pone.0339578.s007.pdf]

>MSTRG.6946.4 gene=SLC1A5
AGAGCCACGCGGCACGCCCGGGAGGCTTTCTCTGGCTGGTAACCGCTACTCCCGGACACCAGACCACCGC
CTTCCGTACACAGGGGCCCGCATCCCACCCTCCCGGACCTAAGAGCCTGGGTCCCCTGTTTCCGGAGGTC
CGCTTCCCGGCCCCCAGATTCTGGCATCCCAGCCCTCAGTGTCCAAGACCCAGGCAGCCCGGGTCCCCGC
CTCCCGGATCCAGGCGTCCGGGATCTGCGCCACCAGAACCTAGCCTCCTGCAGACCTCCGCCATCTGGGG
GCACTCAACCTCCTGGAGCCAAGGGCCCCACGTCCCACCCAGAGAAACTCTCGTATTCCCAGCTCCTAGG
GCCAAGGAACCCGGGCGCTCCGAACTCCCAGCTTTCGGACATCTGGCACACGGGGCAGAGCAGAGAAGCC
TCAGCGCCCAGCCTGGGGAATTTAAACACTCCAGCTTCCAAGAGCCAAGGAACTTCAGTGCTGTGAACTC
ACAACTCTAAGGAGCCCTCCAAAGTTCCAGTCTCCAGGTGCTGTTACTCAACTCAGTCCTAGGAACGTCG
GGTCCTGGGAAGGAGCCCAAGCGCTCCCAGCCAGCTTCCAGGCGCTAAGAAACCCCGGTGCTTCCCATCA
TGGTGGCCGATCCTCCTCGAGACTCCAAGGGGCTCGCAGCGGCGGAGCCCACCGCCAACGGGGGCCTGGC
GCTGGCCTCCATCGAGGACCAAGGCGCGGCAGCAGGCGGCTACTGCGGTTCCCGGGACCAGGTGCGCCGC
TGCCTTCGAGCCAACCTGCTTGTGCTGCTGACAGTGGTGGCCGTGGTGGCCGGCGTGGCGCTGGGACTGG
GGGTGTCGGGGGCCGGGGGTGCGCTGGCGTTGGGCCCGGAGCGCTTGAGCGCCTTCGTCTTCCCGGGCGA
GCTGCTGCTGCGTCTGCTGCGGATGATCATCTTGCCGCTGGTGGTGTGCAGCTTGATCGGCGGCGCCGCC
AGCCTGGACCCCGGCGCGCTCGGCCGTCTGGGCGCCTGGGCGCTGCTCTTTTTCCTGGTCACCACGCTGC
TGGCGTCGGCGCTCGGAGTGGGCTTGGCGCTGGCTCTGCAGCCGGGCGCCGCCTCCGCCGCCATCAACGC
CTCCGTGGGAGCCGCGGGCAGTGCCGAAAATGCCCCCAGCAAGGAGGTGCTCGATTCGTTCCTGGATCTT
GCGAGAAATATCTTCCCTTCCAACCTGGTGTCAGCAGCCTTTCGCTCATACTCTACCACCTATGAAGAGA
GGAATATCACCGGAACCAGGGTGAAGGTGCCCGTGGGGCAGGAGGTGGAGGGGATGAACATCCTGGGCTT
GGTAGTGTTTGCCATCGTCTTTGGTGTGGCGCTGCGGAAGCTGGGGCCTGAAGGGGAGCTGCTTATCCGC
TTCTTCAACTCCTTCAATGAGGCCACCATGGTTCTGGTCTCCTGGATCATGTGATGGAGTTTCACTCTTG
TTGCCCAGGCTGGAGTGCAATGGCATGATCTTGGCTCACAGCAACCTCTGCCTCCCTGATTCAAGCAATT
CTCCTGCCTGAGCCTCCCAAGTAGCTGGGATTGCAGGCATGCGCCACCACGCCTGGTTAATTTTGTATTT
TTAGTAGAGACGGGGTTTCTCCATGTTGGTCAGGCTGGTCTTGAACTCCCGACCTCAGGTGATCCACCCA
CCTCAGCCTCCCAAAGTGCTGGGATTACAGGCATGAGCCACCGTGCCCGGCCTGCACGGAGAGTTTTATC
TCCGGGCTGCTCTACCTTTGAGGGGAAAGAGAGCATGAGATTGGCATGGGGGCTTCAGGGGCCAGGCCTG
GGAGTGGTGCCCATGCTGAAGCACACATTGCTGGGGCTTCTCCTGACCTCAGGAAGGCAGGAAGATGTGG
GTAGGGTGCTGAGCCTCACGCCTGGCCACTGGGCCCGTGGAAGAGGCAACAAGCTGTCTCCTGTGTTACT
GATCAGGTACGCCCCTGTGGGCATCATGTTCCTGGTGGCTGGCAAGATCGTGGAGATGGAGGATGTGGGT
TTACTCTTTGCCCGCCTTGGCAAGTACATTCTGTGCTGCCTGCTGGGTCACGCCATCCATGGGCTCCTGG
TACTGCCCCTCATCTACTTCCTCTTCACCCGCAAAAACCCCTACCGCTTCCTGTGGGGCATCGTGACGCC
GCTGGCCACTGCCTTTGGGACCTCTTCCAGTTCCGCCACGCTGCCGCTGATGATGAAGTGCGTGGAGGAG
AATAATGGCGTGGCCAAGCACATCAGCCGTTTCATCCTGCCCATCGGCGCCACCGTCAACATGGACGGTG
CCGCGCTCTTCCAGTGCGTGGCCGCAGTGTTCATTGCACAGCTCAGCCAGCAGTCCTTGGACTTCGTAAA
GATCATCACCATCCT

>MSTRG.11960.3 gene=ASNS
GATTGTTACGCACCTGACAGCTAGCGGAAGGACCTTTCTGTCCTGGTTCCCTGAAGCATCGAGAGGGAAC
CGCGGCCGGCAGGCCGGACCGCTCCGGAGAGGAGGCAGCGGCAGTTTGAGCCCCTATTTTCTTCAATCAC
ATCTGAATAAATCACTTGAAGAAAGCTTATAGCTTCATTGCACCATGTGTGGCATTTGGGCGCTGTTTGG
CAGTGATGATTGCCTTTCTGTTCAGTGTCTGAGTGCTATGAAGATTGCACACAGAGGTCCAGATGCATTC
CGTTTTGAGAATGTCAATGGATACACCAACTGCTGCTTTGGATTTCACCGGTTGGCGGTAGTTGACCCGC
TGTTTGGAATGCAGCCAATTCGAGTGAAGAAATATCCGTATTTGTGGCTCTGTTACAATGGTGAAATCTA
CAACCATAAGAAGATGCAACAGCATTTTGAATTTGAATACCAGACCAAAGTGGATGGTGAGATAATCCTT
CATCTTTATGACAAAGGAGGAATTGAGCAAACAATTTGTATGTTGGATGGTGTGTTTGCATTTGTTTTAC
TGGATACTGCCAATAAGAAAGTGTTCCTGGGTAGAGATACATATGGAGTCAGACCTTTGTTTAAAGCAAT
GACAGAAGATGGATTTTTGGCTGTATGTTCAGAAGCTAAAGGTCTTGTTACATTGAAGCACTCCGCGACT
CCCTTTTTAAAAGTGGAGCCTTTTCTTCCTGGACACTATGAAGTTTTGGATTTAAAGCCAAATGGCAAAG
TTGCATCCGTGGAAATGGTTAAATATCATCACTGTCGGGATGTACCCCTGCACGCCCTCTATGACAATGT
GGAGAAACTCTTTCCAGGTTTTGAGATAGAAACTGTGAAGAACAACCTCAGGATCCTTTTTAATAATGCT
GTAAAGAAACGTTTGATGACAGACAGAAGGATTGGCTGCCTTTTATCAGGGGGCTTGGACTCCAGCTTGG
TTGCTGCCACTCTGTTGAAGCAGCTGAAAGAAGCCCAAGTACAGTATCCTCTCCAGACATTTGCAATTGG
CATGGAAGACAGCCCCGATTTACTGGCTGCTAGAAAGGTGGCAGATCATATTGGAAGTGAACATTATGAA
GTCCTTTTTAACTCTGAGGAAGGCATTCAGGCTCTGGATGAAGTCATATTTTCCTTGGAAACTTATGACA
TTACAACAGTTCGTGCTTCAGTAGGTATGTATTTAATTTCCAAGTATATTCGGAAGAACACAGATAGCGT
GGTGATCTTCTCTGGAGAAGGATCAGATGAACTTACGCAGGGTTACATATATTTTCACAAGGATTGGAGA
GGGAGAAAGAAAAACTGCTTTGTGTGCCAAAAGCAAAACTCTTGGTGTTTTTGTTTGTGAAATAGGCTCC
TTCTCCTGAAAAAGCCGAGGAGGAGAGTGAGAGGCTTCTGAGGGAACTCTATTTGTTTGATGTTCTCCGC
GCAGATCGAACTACTGCTGCCCATGGTCTTGAACTGAGAGTCCCATTTCTAGATCATCGATTTTCTTCCT
ATTACTTGTCTCTGCCACCAGAAATGAGAATTCCAAAGAATGGGATAGAAAAACATCTCCTGAGAGAGAC
GTTTGAGGATTCCAATCTGATACCCAAAGAGATTCTCTGGCGACCAAAAGAAGCCTTCAGTGATGGAATA
ACTTCAGTTAAGAATTCCTGGTTTAAGATTTTACAGGAATACGTTGAACATCAGGTTGATGATGCAATGA
TGGCAAATGCAGCCCAGAAATTTCCCTTCAATACTCCTAAAACCAAAGAAGGATATTACTACCGTCAAGT
CTTTGAACGCCATTACCCAGGCCGGGCTGACTGGCTGAGCCATTACTGGATGCCCAAGTGGATCAATGCC
ACTGACCCTTCTGCCCGCACGCTGACCCACTACAAGTCAGCTGTCAAAGCTTAGGTGGTCTTTATGCTGT
AATGTGAAAGCAAATATTTCTTCGTGTTGGATGGGGACTGTGGGTAGATAGGGGAACAATGAGAGTCAAC
TCAGGCTAACTTGGGTGTGAAAAAAATAAAAGTCCTAAATCT
