# Supplementary material for: 6-Methoxyflavone targets SLC1A5 to induce ferroptosis in HeLa cells
Source: PLoS One. 2025 Dec 29;20(12):e0339578. doi: 10.1371/journal.pone.0339578 (PMC12747331; doi:10.1371/journal.pone.0339578)
Supplement: S8 File — The original, uncropped, and unadjusted images underlying all blot results. Page 1. The original, uncropped, and unadjusted blot image of GAPDH in the first batch of samples. Page 2. The original, uncropped, and unadjusted blot image of ASNS in the first batch of samples. Page 3. The original, uncropped, and unadjusted blot image of PEX6 in the first batch of samples. Page 4. The original, uncropped, and unadjusted blot image of SCD in the first batch of samples. Page 5. The original, uncropped, and unadjusted blot image of SLC1A5 in the first batch of samples. Page 6. The original, uncropped, and unadjusted blot image of GAPDH in the second batch of samples. Page 7. The original, uncropped, and unadjusted blot image of GPT2 in the second batch of samples. Page 8. The original, uncropped, and unadjusted blot image of RRM2 in the second batch of samples. Page 9. The original, uncropped, and unadjusted blot image of TMSB4X in the second batch of samples. Page 10. The original, uncropped, and unadjusted blot image of VCP in the second batch of samples. Page 11. The original, uncropped, and unadjusted blot image of VDAC3 in the second batch of samples. Page 12. Prestained & Western Blot Marker. (PDF) [file pone.0339578.s008.pdf]

1. GAPDH1

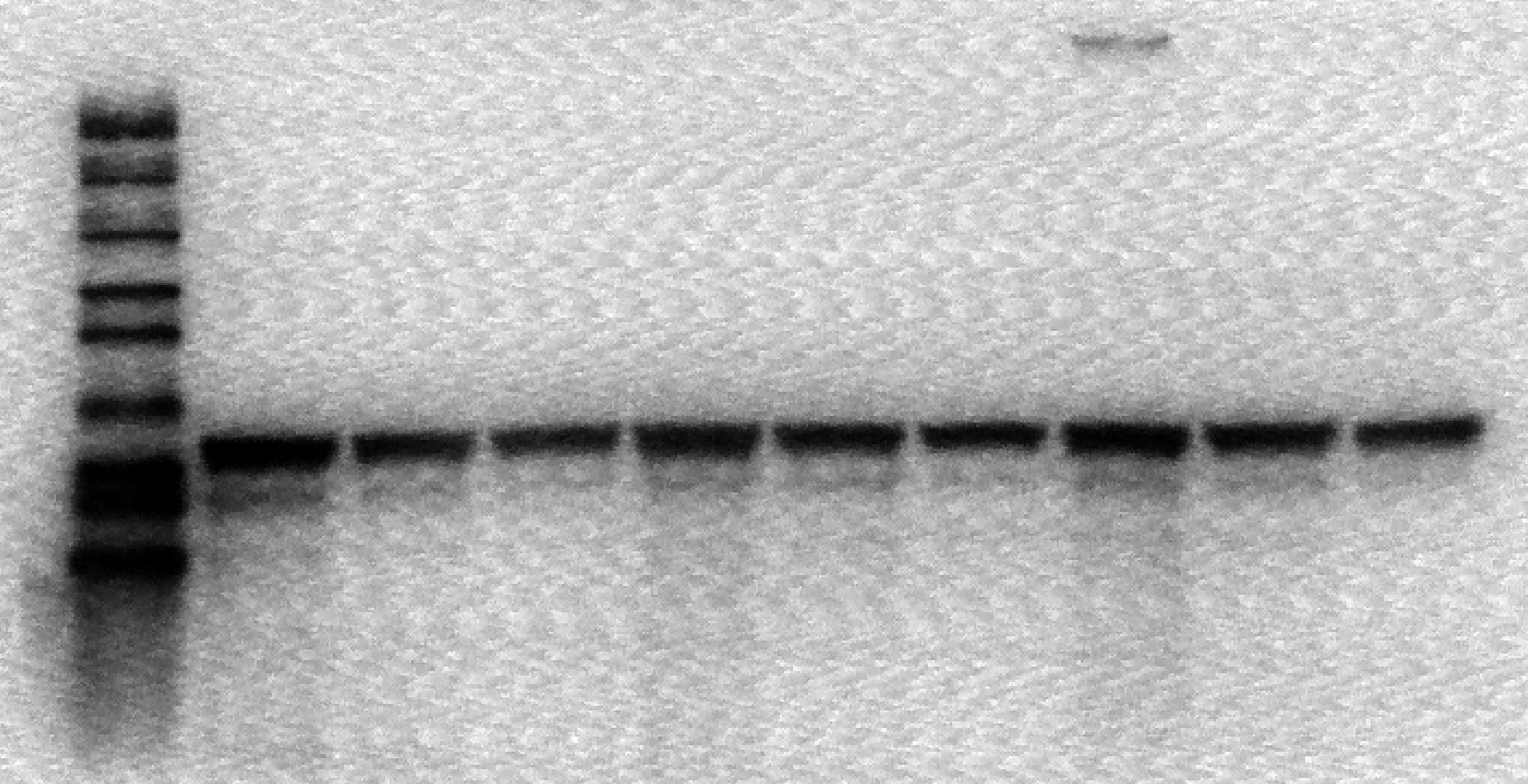

2. ASNS

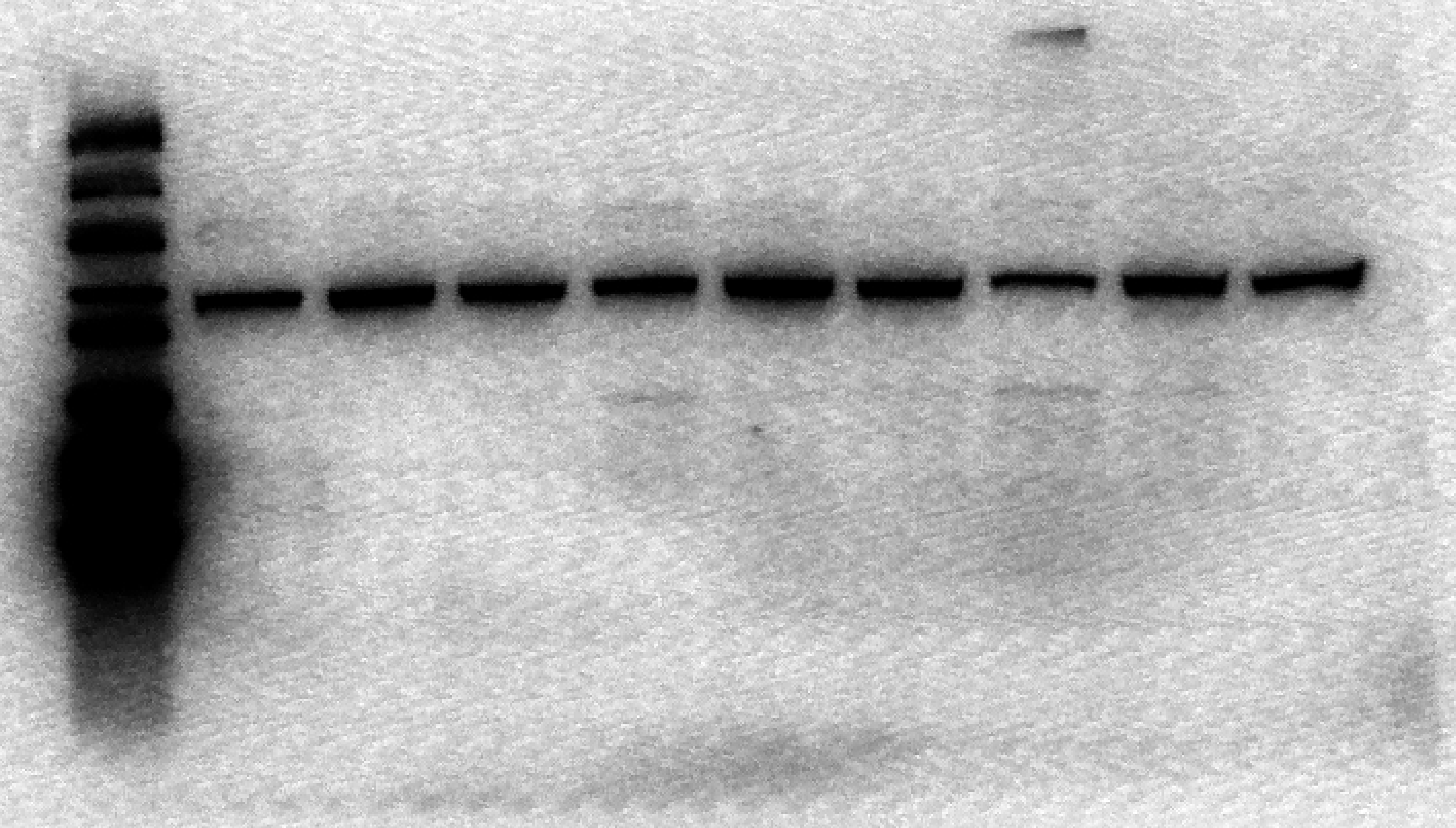

3. PEX6

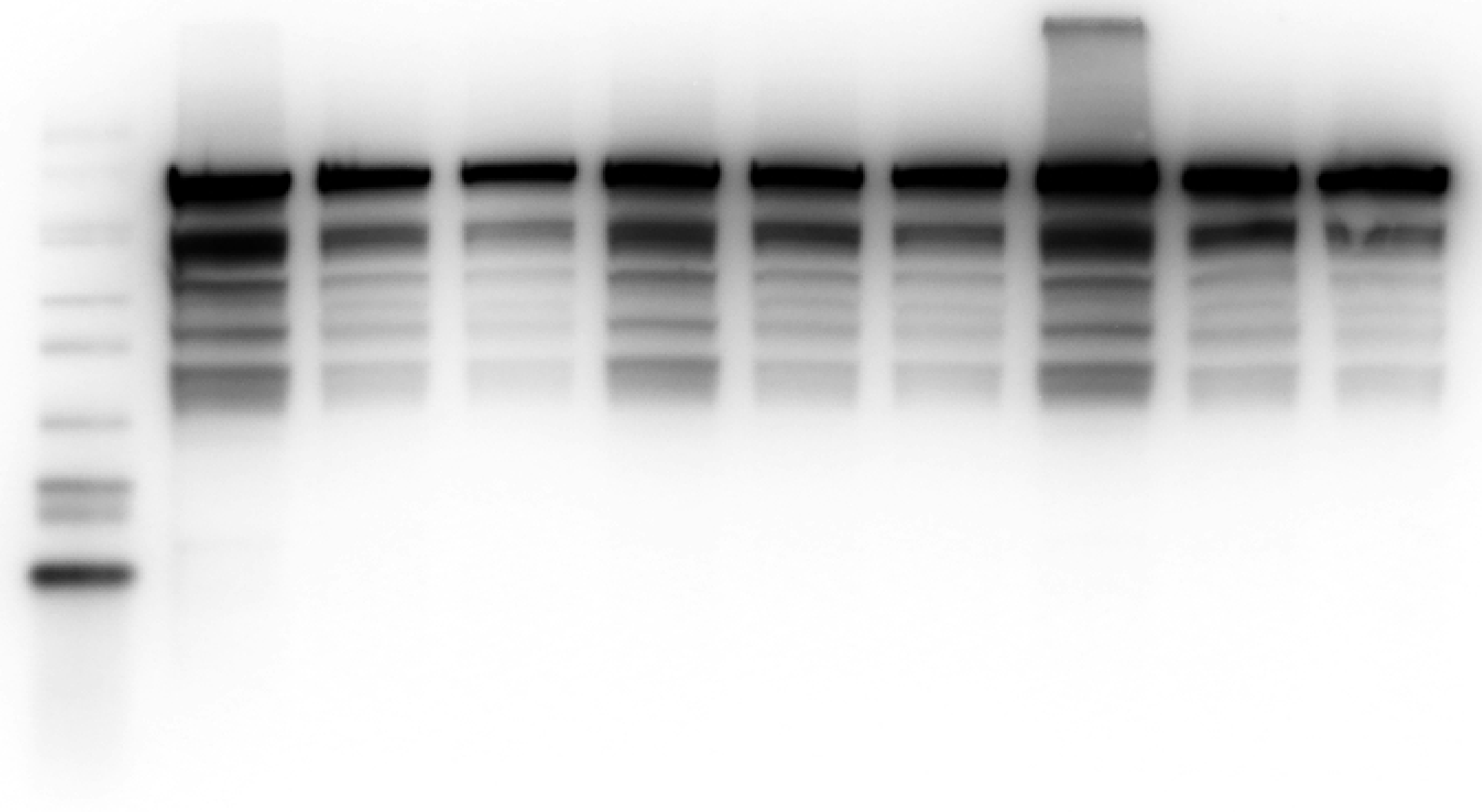

4. SCD

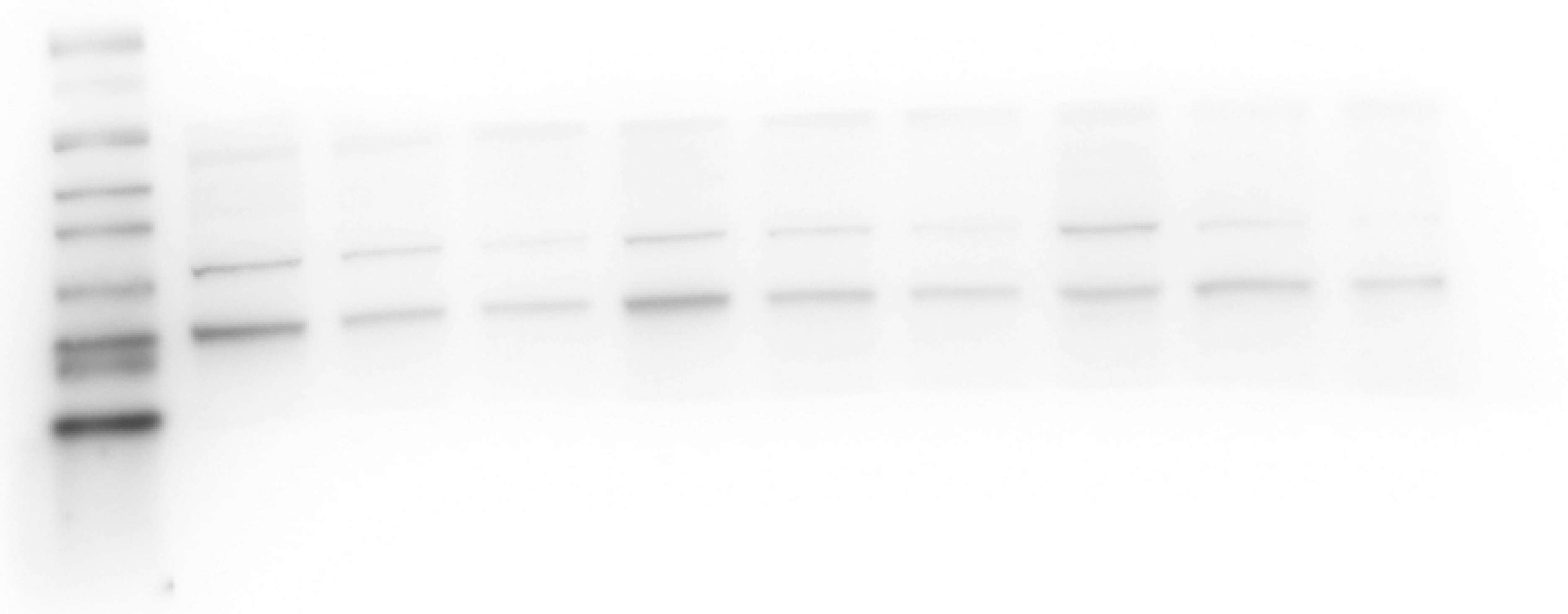

5. SLC1A5

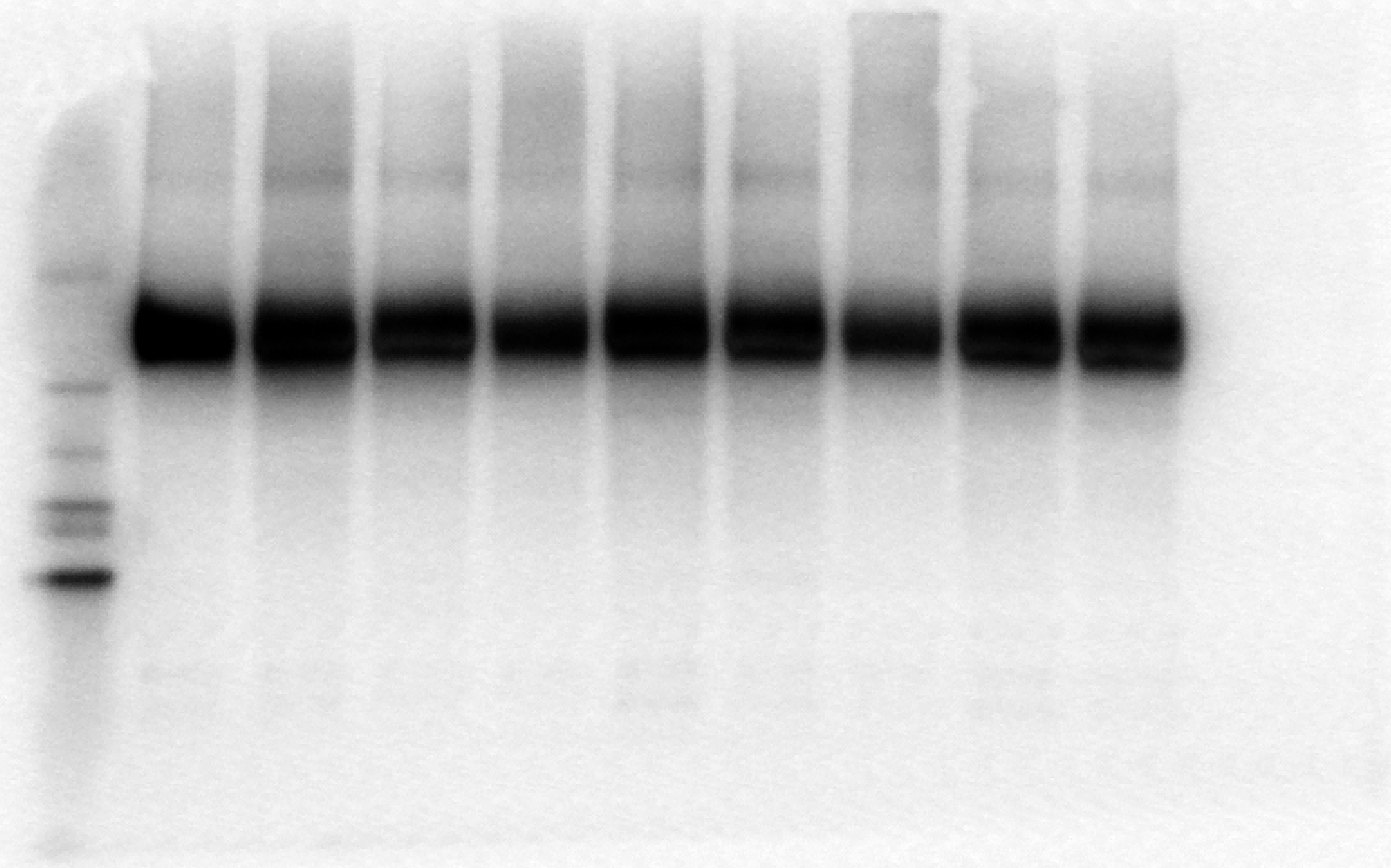

6. GAPDH2

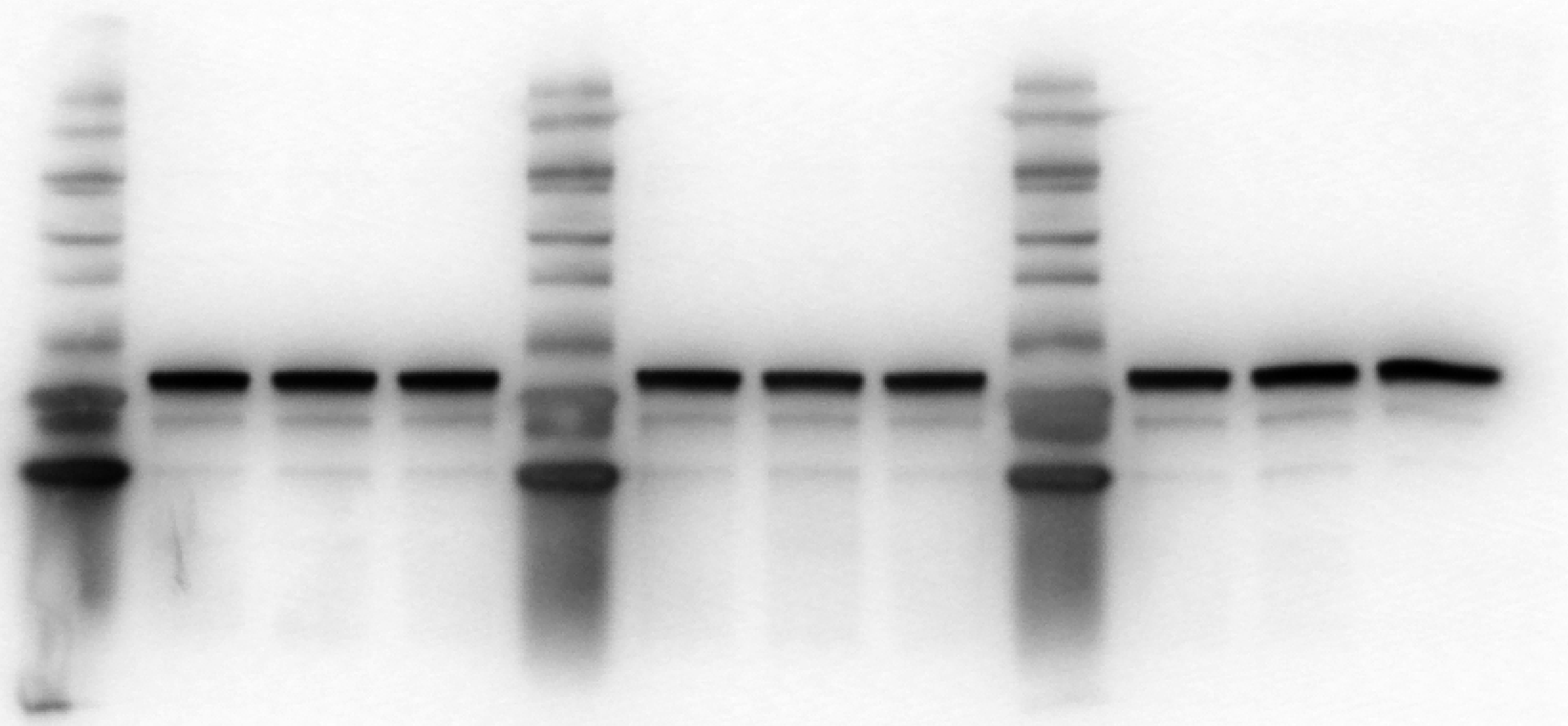

7. GPT2

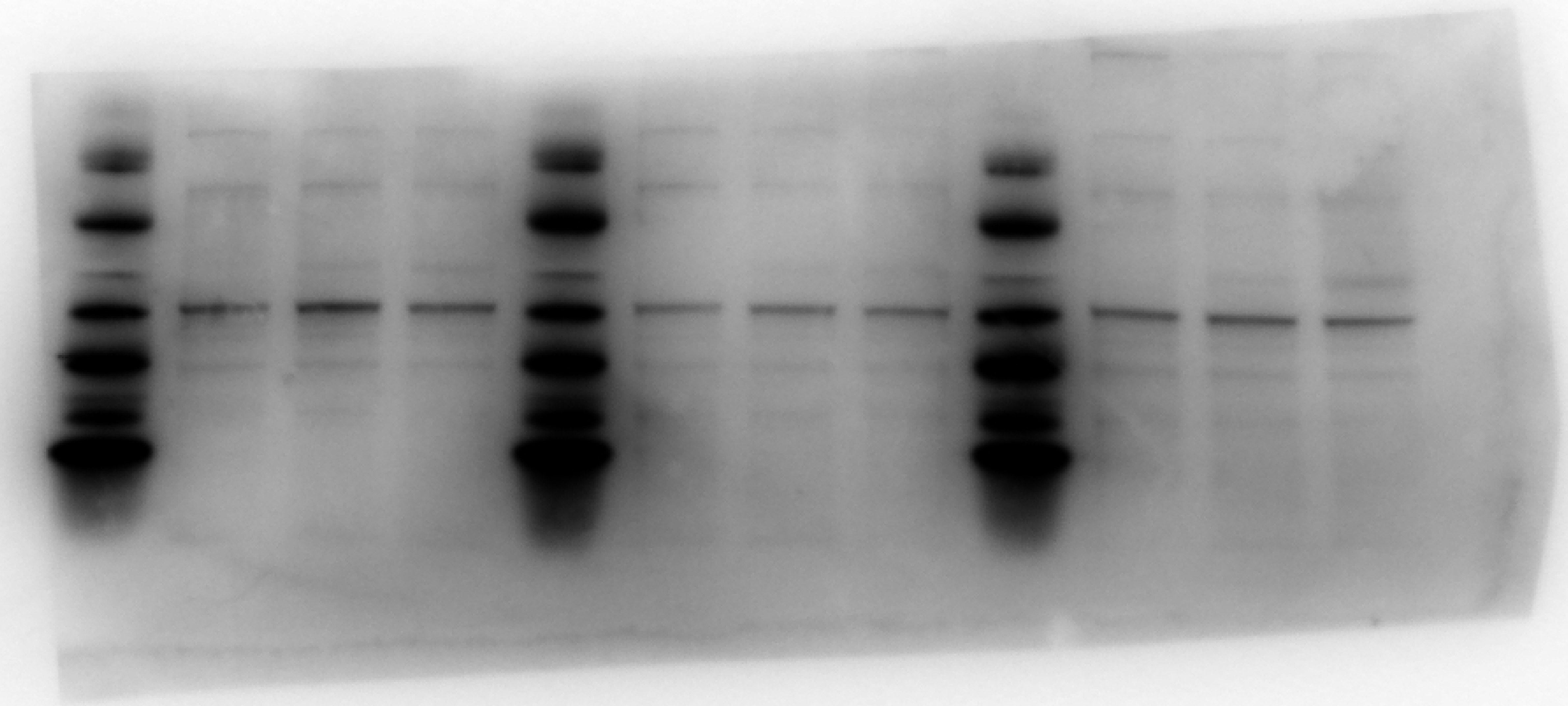

8. RRM2

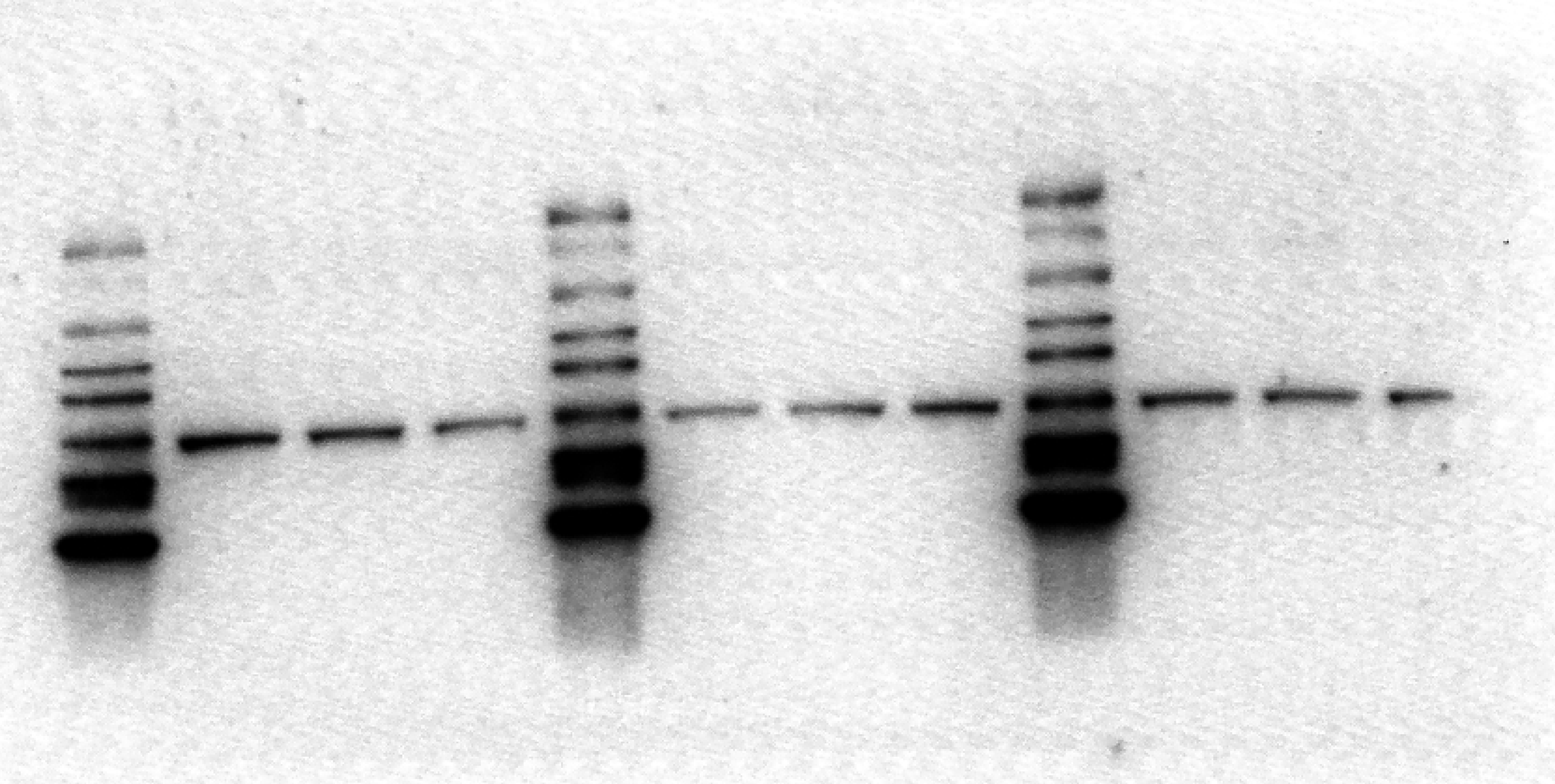

9. TMSB4X

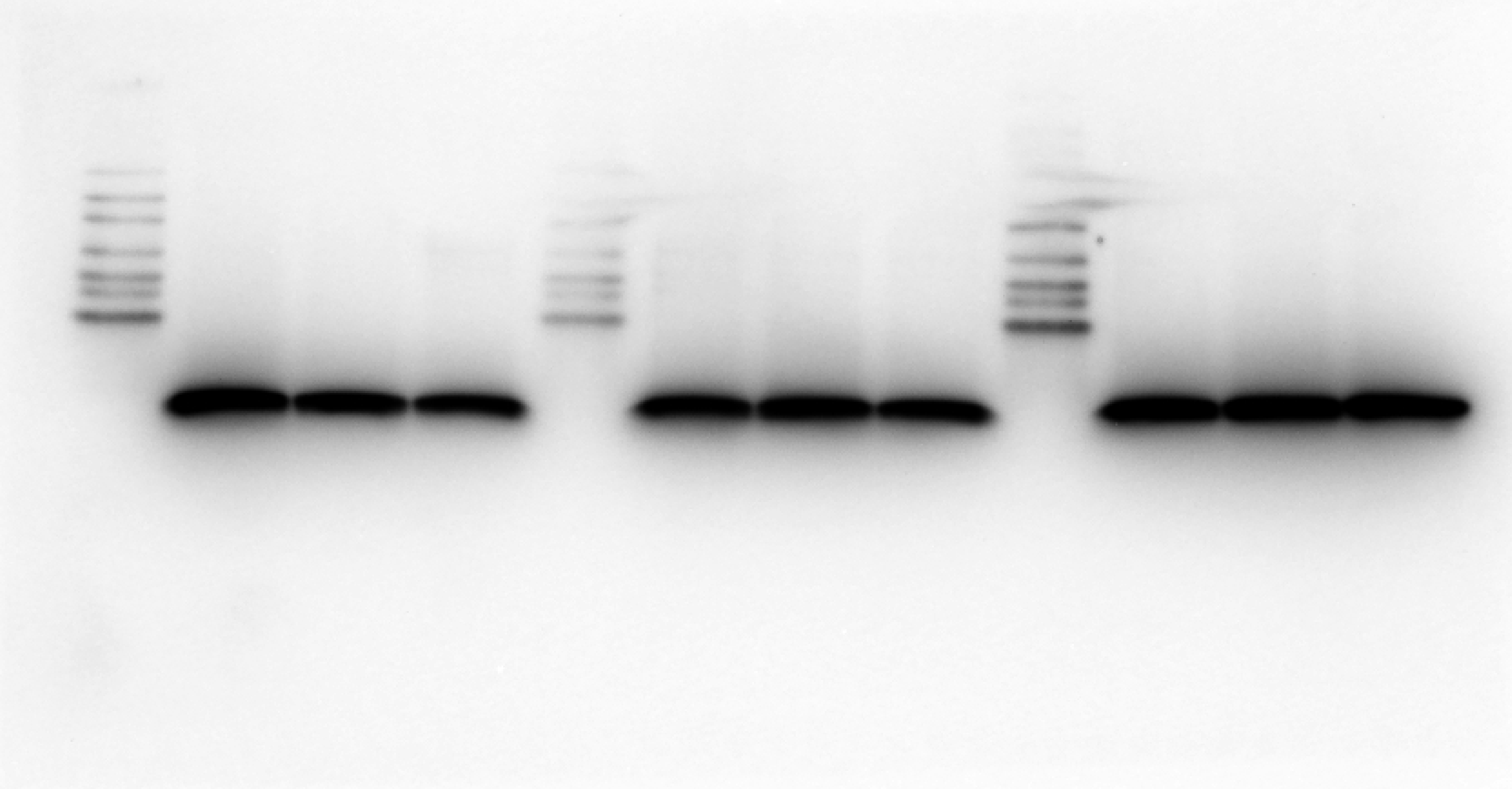

10. VCP

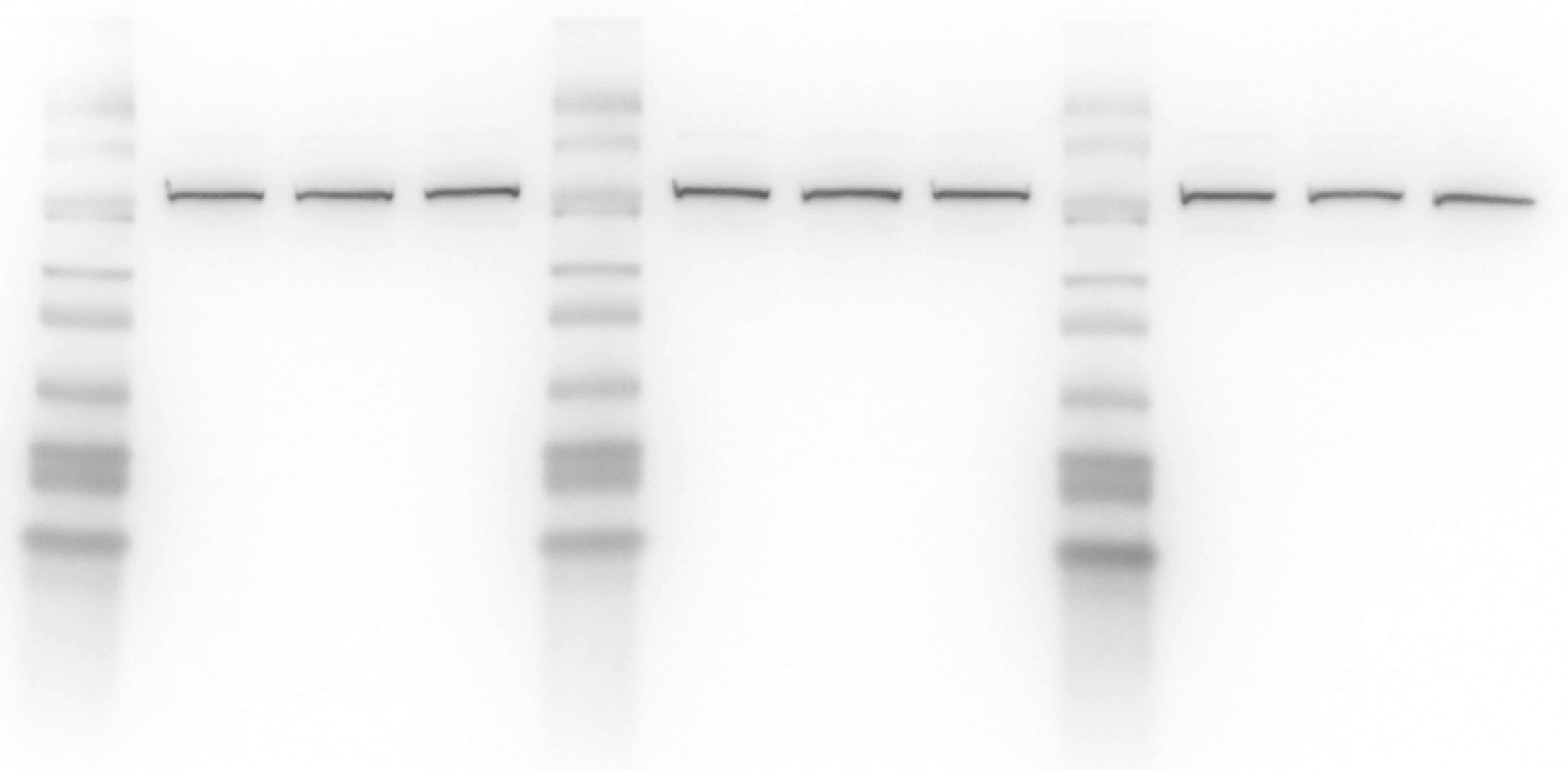

11. VDAC3

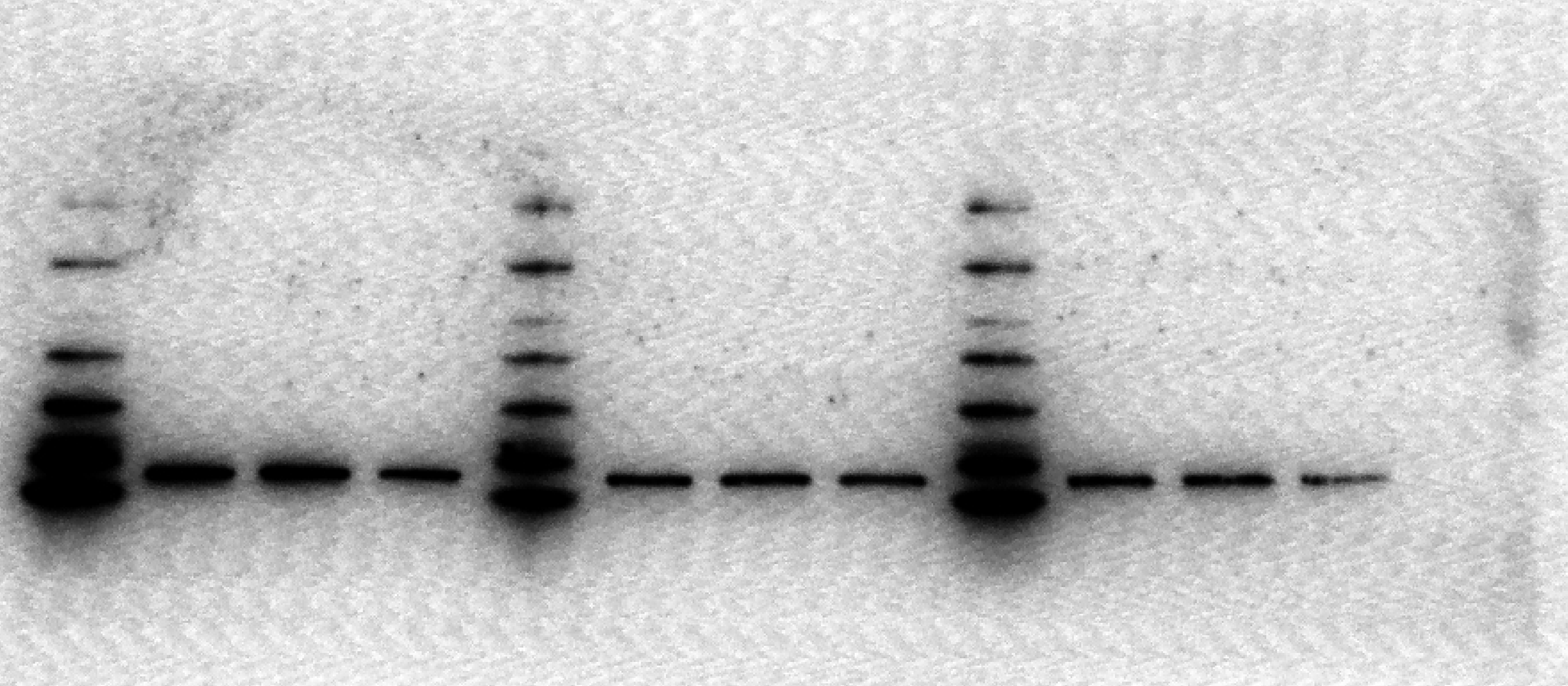

Prestained & Western Blot Marker

Cat.No.: C0302　Size: 100 μl

描述：传统的 Western Blot 试验需要使用预染蛋白 Marker 来跟踪检测阳性抗原信号和转膜效率，但预染蛋白 Marker 无法在胶片上曝光，曝光信号需要根据膜上的预染蛋白 Marker 来判断。HaiGene 全新研发的 Prestained & Western Blot Marker 是专门为 Western Blot 试验设计的分子量标准，由 9 种发光蛋白和 9 种预染蛋白组成；9 中发光蛋白分别为 23、30、36、44、50、62、90、120 和 160KDa，这些蛋白均可与抗体结合，因此能与抗原在同一张膜上曝出信号，阳性信号可以直接通过 Western Marker 的位置来判断；9 种预染蛋白分别为 15、25、35、40、55、70、100、130 和 180KDa，其中 70KDa 为红色，其他为蓝色，转膜完毕后在膜上肉眼可见。

主要特征

- 可与抗原同时检测信号，使 Western blot 结果判断更加简便
- 发光 marker 没有偶联和标记其他分子，分子量更加准确
- 综合发光 marker 和预染 marker 的优势，既能监测转膜又能与抗原同时检测

储存：-20°C 保存，有效期 2 年。

使用方法

待 Prestained & Western Blot Marker 至室温完全融化后，取 2~10 μl（通常 5μl）至上样孔中，进行 SDS-PAGE 电泳；电泳完毕后转至 PVDF 膜或 NC 膜，与一抗二抗孵育；孵育完毕后，将本品与抗原一起通过 ECL 曝光至胶片或荧光显色。

注意事项：

1. 本产品可直接使用，不需要加热。
2. 可根据抗体的效价或曝光时间的长短调整 Prestained & Western Blot Marker 的上样量。
3. 使用新配制的凝胶，及时更换电泳缓冲液和转膜液，以免影响实验结果。

实例

1、HRP 标记的 Goat anti mouse IgG 二抗（一抗：mouse anti p38）ECL 化学发光检测

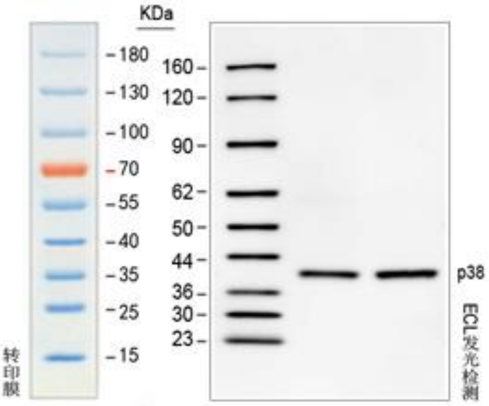

2、多重荧光检测

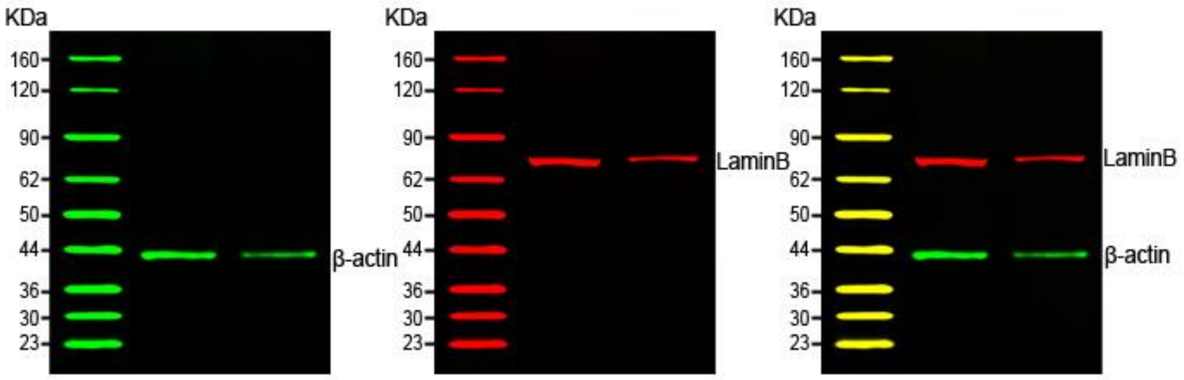

Qdot525 标记的 Goat anti mouse IgG 二抗（一抗： mouse anti β-actin）

Qdot605 标记的 Goat anti rabbit IgG 二抗（一抗：rabbit anti LaminB）

Merged

注意：本产品仅供研究用，请勿用于人体及动物的治疗、临床诊断或作为食品、化妆品、家庭用品的添加剂等用途。
